# Health and Economic Impacts of an Early Labor Induction Policy for High‐BMI Mothers

**DOI:** 10.1002/hec.70143

**Published:** 2026-08-05

**Authors:** Louis Freget, Maria Koch Gregersen

**Affiliations:** ^1^ Université Paris Dauphine‐PSL—LEDa Paris France; ^2^ Department of Economics and Business Economics Aarhus University Aarhus Denmark

## Abstract

We expand the literature on marginal returns to birth interventions by studying a common intervention: early induction of labor for a growing share of pregnancies, high‐Body Mass Index (BMI) women. We exploit Danish guidelines which recommend routine induction at 7 days after the expected due date instead of 10–13 days after for mothers with a pre‐pregnancy BMI of at least 35. Our results show that early labor induction improves immediate maternal and neonatal health, reduces universal nurse visits during the child's first year of life, as well as maternal postpartum depression risks (suggestive).

## 1 Introduction

Following Almond et al. (2010), a dense health economics literature documents the returns of birth interventions and their longer‐run consequences. Still, it is almost nonexistent on a fairly common^1^ intervention: medically initiating labor to prevent the health risks of a pregnancy lasting too long, such as an increased likelihood of stillbirth. At the same time, concerns have been raised about potential side effects of these policies (Maimburg 2020; Spillane 2020; Rydahl, Declercq, et al. 2019).^2^ Because such policies entail both benefits and risks, the key question is not whether they are effective on average, but rather which subgroups may benefit most from earlier induction.

Existing causal evidence is primarily based on samples of low‐risk pregnancies. Yet treatment effects may differ substantially for pregnancies with common risk factors such as maternal obesity, limiting what can be inferred from this evidence. Moreover, the existing literature concentrates almost exclusively on immediate health outcomes, largely overlooking other policy‐relevant dimensions such as health beyond birth, maternal mental health, and parental labor supply and fertility.

This paper provides, to our knowledge, the first piece of causal evidence on a policy which moves the time of routine induction forward based solely on having a high‐Body Mass Index (BMI) prior to pregnancy. This setting allows us to assess whether such a targeted intervention is warranted. We additionally examine broader outcomes, including maternal mental health, fertility, and labor supply. In Denmark, women with a pre‐pregnancy BMI ≥ 35 are recommended routine induction of labor at 7 days after the EDD instead of at 10–13 days after (DSOG 2011). Throughout this paper, we will refer to the policy of routinely inducing women 7 days after the expected due date (EDD) as early induction. We exploit this institutional setting and implement a regression discontinuity (RD) design to causally evaluate the intention‐to‐treat (ITT) effect and the local‐average‐treatment‐effect (LATE) of early routine induction for women who have a pre‐pregnancy BMI close to 35. Since the mother's BMI is recorded in the very *beginning of the pregnancy*, we are less concerned about women manipulating their BMI in response to early induction guidelines (which take effect and are discussed *in the end of the pregnancy*).

Leveraging rich Danish administrative data, we consider all pregnancies going at least 7 days past their EDD in Denmark from 2011 to 2017. As pregnancies with known complications are generally recommended to give birth no later than their EDD, our design allows us to isolate the effect of offering early induction for mothers with no other severe risk factors than having a high pre‐pregnancy BMI. This helps determine if high‐BMI in itself provides a sufficient indication for early induction, a question that has been raised in the medical literature but for which no causal evidence exists (Coates et al. 2020).

This is an important policy question. Routine induction entails both potential benefits and risks, so its desirability may depend on whether it can be targeted toward subgroups with larger expected health gains. High‐BMI mothers face elevated health risks and may therefore benefit more from earlier induction, yet there is no causal evidence on its net effects for this group (Coates et al. 2020). This policy question is of increasing relevance: tens of millions of expectant mothers are overweight or obese in high‐ and middle‐income countries, with prevalence continuing to rise (Chen et al. 2018).^3^

Our first stage is strong: having a BMI just above the threshold yields around 15 percentage‐point (p.p) increase in the risk of being induced early (7–9 days after the EDD)—corresponding to a 187% increase relative to our control group.^4^ The ITT‐reduction in the gestational length (length of pregnancy) corresponds to 0.7 days, but scaling it with the increase in the early induction rate corresponds to around 4 days. This estimate is close to those found in RCTs assessing the effect of labor induction for a general population of low‐risk pregnancies.

First, we find evidence that offering early induction because of high‐BMI improves certain dimensions of immediate health but increases epidural use. There are two potential channels through which early induction could improve birth outcomes: early induction may help ensure birth occurs before the fetus grows excessively large and early induction may avert rare but serious complications, such as stillbirth, that can arise from overly long pregnancy lengths. Indeed, we find that early induction significantly reduces the likelihood of delivering a newborn weighing 4.5 kg or more. While a high birth weight is generally considered a beneficial outcome in the health economics literature (see e.g. Behrman and Rosenzweig (2004); Black et al. (2007); Figlio et al. (2014); Kreiner and Sievertsen (2020)), an excessively large birth weight can make the delivery more difficult and lead to increased risks of complications. Consistently, we find a decrease in complications potentially affected by having a large newborn (birth weight ≥4.5 kg): lacerations (broad definition), hemorrhage (broad definition) and shoulder dystocia (suggestive)—when the infant's shoulders get stuck during delivery. Effects on these outcomes are rarely found in existing studies on moving routine induction forward to a similar point in time for the general population of low‐risk pregnancies (see Supporting Information S1: Appendix A for a detailed review). While we also find that the intervention reduces a severe complication index (a joint measure of stillbirth, infant death and having an Apgar score < 5 at 5 minutes), this effect is not statistically significant. It is hard to draw statistically sound conclusions on complications so rare.^5^

Second, we find evidence that the intervention reduces certain dimensions of healthcare usage after birth. In particular, the number of universal nurse visits during the first year is lower for women who are just above the BMI threshold. Although the estimated effect on GP contacts frequency is not statistically significant, the negative coefficient still points toward a potential reduction speaking against a substitution effect. This is further supported by no evidence of any impact of hospital contacts.

Third, we study the consequences of the policy on maternal mental health during the child's first year of life. Leveraging unique psychometric data from a universal nurse‐visiting program after birth, our ITT estimates indicate that early induction significantly lowers the score used to screen for postpartum depression risk by around 20% of a standard deviation indicating improved maternal mental health. To our knowledge, this is the only causal evidence of a mental health effect of early labor induction. This result is however more suggestive, as it is marginally significant in many specifications and not robust to multiple testing.

Maternal mental health could affect many dimensions of family life including maternal decision‐making power within the family (Baranov et al. 2020), the cognitive and non‐cognitive skills of the children in early childhood (Baranov et al. 2020; Frank and Meara 2009), maternal educational investments (von Hinke et al. 2022), or maternal labor supply decisions (Bryan et al. 2022). Besides improved maternal mental health, birth interventions themselves could also affect labor market decisions through fertility. For instance, Halla et al. (2020) show that avoidable C‐sections may decrease fertility and thereby increase labor market supply in the short term. However, we find no effect on labor supply in our setting 2 years after birth, nor on the probability of having another child within 2 years after birth. Similarly, Gregersen (2024) finds no effect of labor induction for the broader population of low‐risk pregnancies up to 3 years after birth, but she also did not find evidence of any immediate health effects.

We probe the robustness of these findings through multiple checks, including balance of observables, model‐specification checks, multiple hypothesis testing, and two placebo analyses: one restricting on births between 2004 and 2009 (before the issuance of the pre‐pregnancy BMI‐based early induction guidelines) and another one in which we restrict the sample to women who gave birth before seven days past their EDD, who thus were unaffected by the BMI‐based early induction policy but still exposed to other BMI‐based guidelines.

We contribute to the literature by offering causal evidence on one targeted birth intervention directly relevant for clinicians' decision making: early routine induction for high‐BMI women. While we are the first to provide causal evidence on this specific policy, we also expand the broader literature on labor induction by examining health outcomes beyond immediate health, including postpartum mental health. Furthermore, our findings also speak to a large body of literature in health economics studying the marginal returns of medical care, following the seminal work of Almond et al. (2010). A strand of this literature has focused on birth interventions including C‐sections (Jensen and Wüst 2015; Costa‐Ramón et al. 2018; Halla et al. 2020; Mühlrad 2022; Card et al. 2023), interventions at very low birth weight cutoffs (Almond et al. 2010), home births (Daysal et al. 2015), being discharged from the hospital (Sievertsen and Wüst 2017; Card et al. 2023), and hospital crowding (Hoe 2022). Closely related are also studies investigating the timing of birth (either through C‐sections or inductions) for different reasons and at different margins. For example, Buckles and Guldi (2017) study how reducing planned deliveries two to three weeks *prior* to the EDD improved mother and child health outcomes, while multiple studies investigate how economic incentives may affect the timing of birth which in turn may affect mother and child health (Gans and Leigh 2009; Neugart and Ohlsson 2013; Schulkind and Shapiro 2014; Borra et al. 2019; Jürges 2017).

However, to our knowledge, the only economic paper that analyzes a specific labor induction policy is Gregersen (2024). Both her study and ours are set in the context of 2011 Danish recommendations, but she exploits a different variation caused by the *introduction* of the new guidelines for low risk pregnancies. Despite a large increase in the induction risk, she finds no severe consequences of introducing additional surveillance and moving routine induction forward to 10–13 days after the EDD instead of at 14 days after for low‐risk first‐time pregnancies. By contrast, we rely on a within‐period variation (post‐2011) stemming from separate guidelines on induction of high‐BMI women. We therefore study not only a higher‐risk population, but also a different policy margin: offering routine induction at 7 days after the EDD rather than at 10–13 days. Our findings indicate beneficial effects for high‐BMI women, highlighting that the optimal timing of induction depends on maternal risk profiles and that targeted induction policies may improve outcomes relative to uniform guidelines.

We also enrich the scarce economic literature on the effect of birth interventions on postpartum depression. While Tonei (2019) finds increased risks of maternal postpartum depression when having an unplanned C‐section, our findings suggest that other (milder) types of birth interventions may also impact maternal postpartum depression risks. Thus, our findings highlight the importance of evaluating potential mental health effects of birth interventions.

## 2 Institutional Setting

Since 2011, Danish women with a pre‐pregnancy BMI ≥ 35 have been recommended induction at 7 days after the EDD^6^ even in the absence of any other pregnancy risks (DSOG 2011). Similar women with a pre‐pregnancy BMI < 35 were generally recommended induction at 10–13 days after the EDD. Importantly, this BMI cutoff for early routine induction of labor applies only to relatively uncomplicated high‐BMI pregnancies that do not have other complications warranting an even earlier induction or a planned C‐section.^7^

In Figure 1, we empirically show that the rate of births with induced labor rises from 7 days after the EDD for women above the BMI≥35 cutoff relative to women with a BMI < 35. At 10 days after the EDD, the induction rate for women with a BMI < 35 begins to rise too, and at around 12 days after the EDD the induction rates for both groups have converged.

**FIGURE 1:**
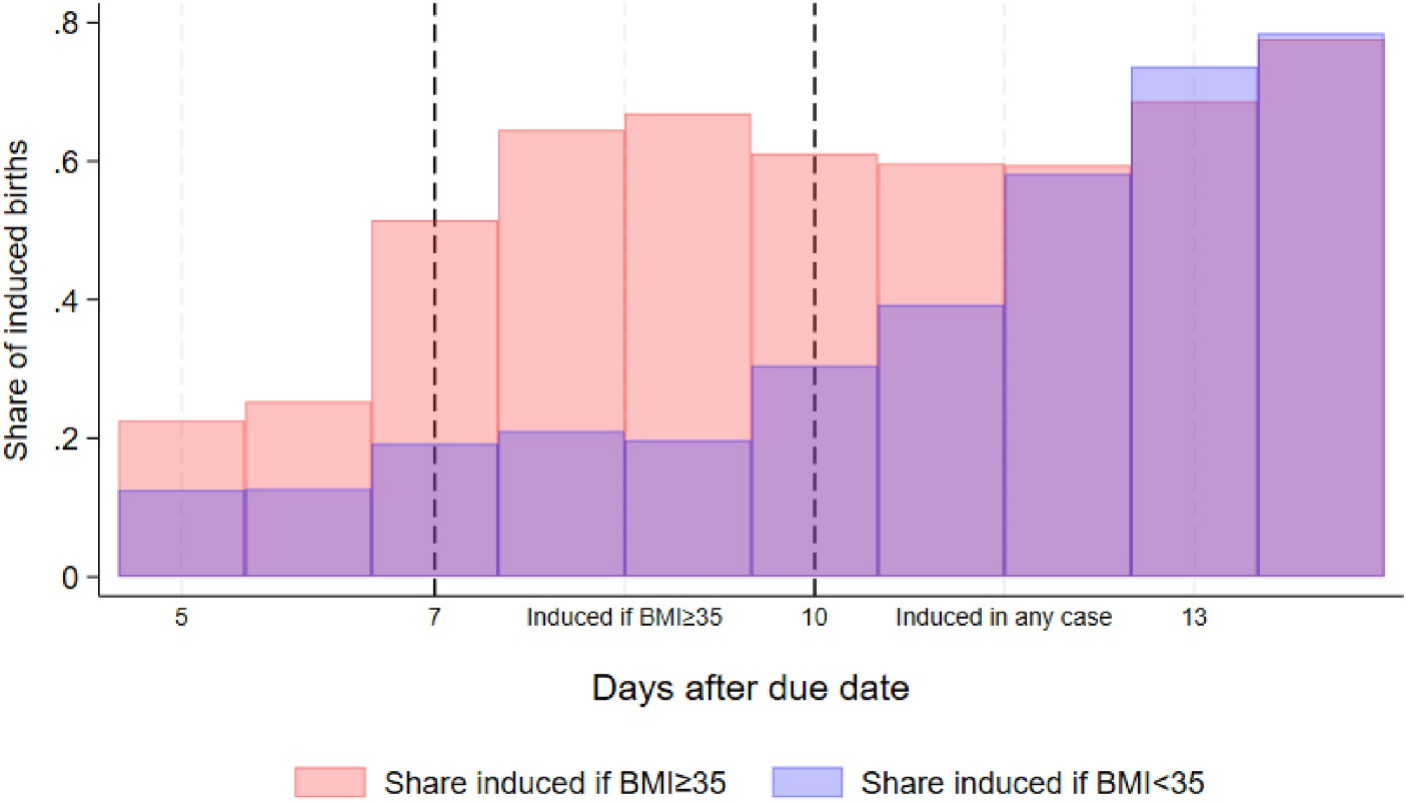
Average induction rates for ongoing births by days after the due date. The figure shows the share of births with induced labor by day after the estimated due date (EDD). The sample includes all births in Denmark between 2011 and 2017 with a gestational length of more than four days past the EDD and with information on maternal BMI.

Since we show the rate of induced labor by the time of birth (and not the time of induction), it is to be expected that the relative difference continues to rise during 7–9 days after the EDD as depicted in Figure 1. First, a woman may not necessarily go into labor on the day of induction.^8^ Second, hospitals tend to postpone induction of labor on busy days (Maibom et al. 2021). Third, even when labor is successfully initiated at the day of induction, the duration of labor may in many cases last more than one calendar day.

Thus, a woman with a pre‐pregnancy BMI just above the cutoff and an otherwise uncomplicated pregnancy will potentially be affected by the guidelines in two ways relative to a similar woman just below the cutoff. First, she is more likely to give birth earlier (reduced gestational length). Second, she may have a different birth mode. Had she not been induced at 7 days after the EDD, she might go into spontaneous labor before the general time of routine induction of labor at 10–13 days after the EDD or she would be recommended labor induction at that point in time.^9^ When complying with the guidelines, mothers are thus exposed to *early* induction instead of the alternative which may involve later induction of labor or later spontaneous onset of labor.

The guidelines we exploit were introduced in 2011 as a part of a broader reform aimed at reducing the risk of stillbirth by moving routine induction forward for all women, while balancing the risks of induction. Women with a high BMI face a higher baseline risk of both prolonged pregnancy and stillbirth. As a precautionary measure, the guidelines recommended earlier induction for this group of women. However, at the time, there was no causal evidence on whether such a policy would improve outcomes for high‐BMI women, nor clear evidence supporting the specific threshold at BMI 35. Our study therefore provides evidence on whether high‐BMI women benefit from early induction based solely on their BMI.

As for the target population, it is important to note that this BMI cutoff for early routine induction of labor applies only to relatively uncomplicated high‐BMI pregnancies that do not have other complications warranting an even earlier induction or a planned C‐section. Indeed, pregnancies with obstetrical complications are typically recommended induction or elective C‐section no later than their EDD (DSOG 2011; Hedegaard et al. 2014). Those types of pregnancies include cases of insulin‐treated (gestational) diabetes, intrauterine growth restriction, preeclampsia, a history of previous intrauterine death, or a multiple pregnancy. Only mild cases of gestational diabetes (GDM) with no need for insulin treatment^10^ and with no suspicion of a large baby^11^ are allowed to pass their EDD together with some type of low‐risk pregnancies with a previous C‐section. Moreover, pregnancies may be induced even earlier if any problems are detected during surveillance or any type of complication occurs. Thus, we estimate the effect of early induction for high‐BMI mothers without any other major complications.

This policy setting allows us to address a central question in the medical and birth policy literature: although early induction is widely recommended when having a high BMI leads to conditions like preeclampsia, the net benefit of early induction solely because of high BMI is currently uncertain.

## 3 Data

This paper combines data from several administrative sources. Most of our health outcomes and control variables are extracted from the Danish Medical Birth Register (MFR), the National Patient Register (LPR), Danish National Health Service Register for Primary Care (SYSI), and the Death Records. Additional sources of administrative data from Statistics Denmark allow us to get family background information. Nurse records from Danish municipalities provide us with mental health data and nurse visits data. Our unit of observation is the birth for maternal outcomes, but when evaluating children's outcomes the number is slightly larger because of a small number of multiple births. For details on our control variables see Supporting Information S1: Appendix C.

### 3.1 Pre‐Pregnancy BMI

The MFR includes a measure of mothers' pre‐pregnancy BMI. It is calculated based on reported weight and height at the mother's first pregnancy consultation taking place at her family GP around pregnancy week 6–10. The available measure disregards extreme cases where the mother's weight is either lower than 30 kg or higher than 249 kg or her height is either less than 50 cm or taller than 210 cm. We additionally put BMI values > 44 to missing (0.05%). We can observe the mother's pre‐pregnancy BMI for 92% of all pregnancies recorded in the MFR during the years 2011–2017 (our main sample period) and which passed the EDD with at least 7 days. Since the available measure of pre‐pregnancy BMI is 3‐digit, we treat it as a continuous measure.

### 3.2 Instrument for Early Induction

To obtain a measure of the intended treatment when complying with the guidelines, we construct a measure of *early* induction. The medical birth register contains information on time of birth and whether labor was induced, but not the *time* of labor induction. Instead we use information from LPR and follow Hedegaard et al. (2015)'s Danish medical study in defining (medically) induced labor as any of the following procedure codes: BKHD2, BKHD20, BKHD20A, and BKHD21.^12^ Our indicator of early induction is equal to one when any of these procedure codes is registered between 7 and 9 days after the EDD. We allow for the fact that inductions tend to be postponed on busy days (Maibom et al. 2021) but require that the registered inductions take place prior to the point in time where mothers below the BMI threshold potentially face recommendations of routine induction too.

### 3.3 Immediate Health & Birth Outcomes

We assess the impact of the intervention on available outcomes commonly found in the medical literature on induction (see Supporting Information S1: Appendix A for a review). From the Danish Medical Birth Register, we extract information on pregnancy length, whether labor was induced, the use of epidural, vacuum extraction, birth weight, birth weight ≥4.5 kg (macrosomia), and general measures of the child's health (Apgar score < 7 at 5 minutes, admission to the Neonatal Intensive Care Unit (NICU), and uterus rupture). Note that while a higher birth weight may be linked to beneficial longer‐term outcomes (Behrman and Rosenzweig 2004; Black et al. 2007; Figlio et al. 2014; Kreiner and Sievertsen 2020), a birth weight ≥4.5 kg includes risks for both the mother and child and is thus not considered beneficial.^13^

We supplement with additional information from LPR. First, we use information on emergency C‐section (ICD‐10 code: O821). Second, we add information on shoulder dystocia (ICD‐10 code: O660), lacerations (ICD‐10 codes: 070), severe lacerations (3rd, fourth) as well as high obstetric lacerations (ICD‐10 codes: O702, O703 and O714),^14^ and postpartum hemorrhage (O72). The latter code records any kind of hemorrhage since 2012 (Rydahl et al. 2019). We interpret it as a proxy of relatively severe bleeding assuming that it is more likely to be recorded the larger the bleeding.

To study rare but severe outcomes, we pool Apgar score at 5 minutes less than 5 with child mortality (encompassing stillbirth and infant death which is defined as death up to a year of age).^15^ Still, the index is positive in fewer than 10 observations on each side of the cutoff. Caution is warranted when analyzing effects on such a rare outcome.

### 3.4 Short‐Term Health Outcomes

We study different type of measures of health care usage during the first year of life. From the Danish National Health Service Register for Primary Care (SYSI), we extract the number of GP contacts for the child during the first year of life. From the LPR, we construct two separate indicators for whether the mother and child have been in contact with the hospital during the first year of life (but after they have been discharged from birth). To obtain the number of nurse‐visits during the first year of life as well as a detailed measure of mother's mental health, we leverage data from the Danish universal nurse home visiting program (NHV). The goal of these Nurse visits is to monitor family health including parental mental health, support infant development, and refer families with needs to other specialists. The program proposes up to five universal nurse visits within the first year after childbirth. Additional visits may be scheduled by the nurse when needed. In the spirit of Hirani et al. (2020), we use three outcomes to measure nurse visits: universal visits (out of five), additional nurse visits, and a total measure summing both universal and additional nurse visits.

During nurse visits, mothers are offered a mental health screening by a standard home visit by the nurse around 2 months after birth. The most frequently used instrument for screening is the Edinburgh Postnatal Depression Scale (EPDS) (Cox and Holden 2003; Smith‐Nielsen et al. 2018) ranging from 0 to 30 with 30 being the most adverse outcome. We create an indicator for the score being strictly above 11 (12 or more) which is the cutoff for detecting moderate to severe postpartum depression (in the sense of ICD‐10) in the Danish version of the scale (Smith‐Nielsen et al. 2018). We additionally consider the total EPDS score. We are only able to obtain data on nurse visits from 62 out of 98 Danish municipalities. For the EPDS scores, we further need to restrict the sample to the 58 municipalities that systematically register their screening for maternal mental health problems.^16^

### 3.5 Sample Selection and Size

Our initial sample is the universe of 472,051 births between 2011 and 2017 in Denmark. Restricting the sample to births with information on gestational length and maternal BMI yields a sample of 456,321 births. Next, we restrict to all births taking place 7 days after the EDD or later (around 20% of the initial sample). Finally, we also restrict on the mother being less than 40 years old and without a GDM diagnosis^17^ before the due date, as these mothers are recommended early induction regardless of their pre‐pregnancy BMI. Our final sample hence contains 97,604 births.^18^ Descriptives on our final sample as well as data on how risk factors vary across the BMI cutoff are presented in Supporting Information S1: Appendix Table C1. We also build two placebo samples using the same restrictions. The first sample includes births from 2004 to 2009, before high‐BMI mothers were recommended for early induction.^19^ The second sample includes births from 2011 to 2017 but is restricted to cases with a gestational length of less than 7 days past the EDD.

## 4 Methodology

### 4.1 Empirical Methodology and Interpretation

^20^Our identification strategy relies on the continuity of potential outcomes for women just below and just above the BMI threshold. In particular, we exploit that national guidelines discontinuously change at the cutoff of having a BMI of at least 35 using a RD design. Our reduced‐form equation of interest models the maternal and neonatal outcomes as a flexible function of the forcing variable (BMI of mother i) and a “jump” at the guideline‐induced threshold:

(1) $$y_{\mathrm{ihtj}}=\gamma_{0}+\gamma_{1}\left(BMI_{\mathrm{iht}}\geq 35\right)+g\left(BMI_{\mathrm{iht}}\right)+\Phi^{\prime }X_{\mathrm{iht}}+\phi_{h}+\tau_{t}+\psi_{m}+\mu_{d}+\epsilon_{\mathrm{iht}}$$

Where $y_{\mathrm{ihtj}}$ is the considered outcome observed in calendar year j for mother or child i, giving birth/being born at hospital h in calendar year t, month‐of‐birth m, and day‐of‐week d. $X_{\mathrm{iht}}$ is a vector of control variables and g() is a smooth function of the mother's pre‐pregnancy BMI which is allowed to differ at each side of the cutoff. Our main sample includes all pregnancies going at least 7 days after the EDD to restrict attention to those affected by the guidelines. In the same spirit, we exclude mothers with GDM or who are older than 40. As described in Section 3.5, these conditions are in themselves advised to be induced at 7 days after the EDD regardless of BMI. Moreover, we control for the following pregnancy variables: having a multiple birth, previously having given birth to a large child (birth weight ≥4.5 kg), previously having had a C‐section, first‐time mother, insufficient fetal growth, and pre‐eclampsia. Most women with these conditions are recommended giving birth prior to 7 days after the EDD, so they should not affect our identifying variation but may still impact the time and method of delivery. We measure all our pregnancy control variables prior to the EDD to avoid having bad controls (some of these conditions may be discovered at the surveillance taking place around 7 days after the EDD). As specified in Supporting Information S1: Appendix C, to increase precision, we additionally control for parental background characteristics: a dummy if either parent graduated from college, monthly joint parental income in 2024 US dollars, maternal age, a dummy which equals one if either parent was born in Denmark, a dummy for whether parents cohabit, as well dummies for missing information on any of the control variables. Our set of controls also includes hospital fixed effects, $\phi_{h}$, to capture local variation in practices and year fixed effects, $\tau_{t}$, to capture general time trends. Moreover, we include month‐of‐birth fixed effects, $\psi_{m}$, to capture seasonal variation and day of the week of birth fixed‐effects, $\mu_{d}$ to account for the fact that certain birth interventions are more likely to be scheduled at certain days of the week to add precision to our results.^21^

Our reduced form estimate of $\gamma_{1}$ captures the average treatment effect (ATE) effect of *offering* routine induction at 7 days after the EDD instead at 10–13 days later for mothers with a BMI just above 35. Notice that compliance is not perfect, so alternatively it can be interpreted as the intention‐to‐treat (ITT) effect of being induced early (7–9 days after the EDD). This is a policy‐relevant parameter for policy‐makers considering moving the time for routine induction early for high‐BMI women.

To account for non‐compliance and get at the causal impact of being induced early when having a BMI close to 35, we turn to a fuzzy RD design which re‐scales potential jumps at the cutoff in our key outcomes by the jump at the cutoff in the probability of being induced early (defined between 7 and 9 days after the EDD as specified in Sub‐Section 3.2). Formally, this is equivalent to instrumenting a dummy for “Early Induced Labor” by the jump in the guideline‐induced BMI threshold:

(2) $$y_{\mathrm{ihtj}}=\gamma_{0}+\beta_{1}\text{Early}\;\text{Induced}\;\text{Labor}+g\left(BMI_{\mathrm{iht}}\right)+\Phi^{\prime }X_{\mathrm{iht}}+\phi_{h}+\tau_{t}+\psi_{m}+\mu_{d}+\epsilon_{\mathrm{iht}}$$

Here, “Early Induced Labor” is a dummy which equals 1 if the mother giving birth was induced between 7 and 9 days after the EDD. The fuzzy RD estimates are IV estimates of $\beta_{1}$ from Equation (2). It captures the local average treatment effect (LATE) at the threshold: the effect stemming from being induced at 7–9 days after the EDD because the mother's pre‐pregnancy BMI is just above the 35 cutoff.

Among those who are induced early because of mother's pre‐pregnancy BMI, some will have a changed mode of labor (induced instead of spontaneous) as well as a reduced gestational length. Others will only have a reduced gestational length as they would have been induced anyway, but at a later point in time had their BMI been just below the cutoff. Any impact of treatment (early induction) may run either through reduced gestational length or a combination of changed mode of labor and reduced gestational length.

Formally, we estimate our empirical models using local polynomial regression using Calonico et al. (2017)'s ‐rdrobust‐ command. In all of the analyses, we display bias‐corrected estimates with the nonparametric robust standard errors clustered at the yearly hospital level. In Supporting Information S1: Appendix D.3, we show that for many of our outcomes we do not have enough power to use data‐driven bandwidth methods (see Supporting Information S1: Appendix Table D5). One key reason is that there are far fewer births above the cutoff than below it (see Supporting Information S1: Appendix Figure D6). While the data‐driven bandwidth is usually between 3 and 5, we opt for a slightly larger manual bandwidth of 7 and a polynomial order of one. Besides powering reasons, there is a theoretical rationale for the choice of a bandwidth of specifically 7: it ensures that all women in our sample undergo at least one glucose test to detect cases of GDM during pregnancy (see Supporting Information S1: Appendix B for details on high‐BMI pregnancies in Denmark). Note that these specification choices are only relevant in our main specification: we indeed check that our results are robust to changes in bandwidth, kernel and polynomial order in Supporting Information S1: Appendix Figures D10 and D11. Supporting Information S1: Appendix Tables D6–D7 further shows stable results under alternative covariate sets, including specifications without controls, with fixed effects only, with medical controls only, as well as with heteroskedastic standard errors instead of clustering at the hospital‐year level.^22^ We also compute *p*‐values adjusted for multiple hypothesis testing using Romano‐Wolf's procedure available through the package created by Clarke et al. (2020).

Validity of our ITT estimates requires that the continuity assumption is met which ensures local variation across the 35 cutoff. This requires that in absence of the treatment, the potential outcomes would have evolved smoothly around mother's pre‐pregnancy BMI equal to 35. Our Fuzzy RD design additionally requires that: (a) the BMI ≥ 35 cutoff implies a substantial increase in labor induction between 7 and 9 days after the EDD (strong first‐stage), (b) the monotonicity assumption holds, and (c) the exclusion restriction holds. We are not particularly worried about the monotonicity assumption in our context (we do not find it likely that a mother who refuses labor induction at 7–9 days after the EDD because her pre‐pregnancy BMI is at least 35 would have accepted that had her BMI been lower than 35). Instead, the exclusion restriction provides a bigger challenge by requiring that the 35 cutoff may only impact mothers through their changed early induction status. The following sections are devoted to addressing the main threats to the key identifying assumptions in our setting.

### 4.2 Threats to Identification

Our key assumption for obtaining valid ITT estimates is the continuity around the cutoff assumption. First, it excludes manipulation of the running variable. In theory, a mother might try to manipulate her weight to be either above or below the BMI threshold in which she is more likely to be early induced. In practice, such manipulation is not likely since the mother's pre‐pregnancy BMI is reported in the beginning of the pregnancy to the family GP whereas induction guidelines are typically discussed later during pregnancy with the midwife. Empirically, Figure 2 rules out manipulations of that kind.

**FIGURE 2:**
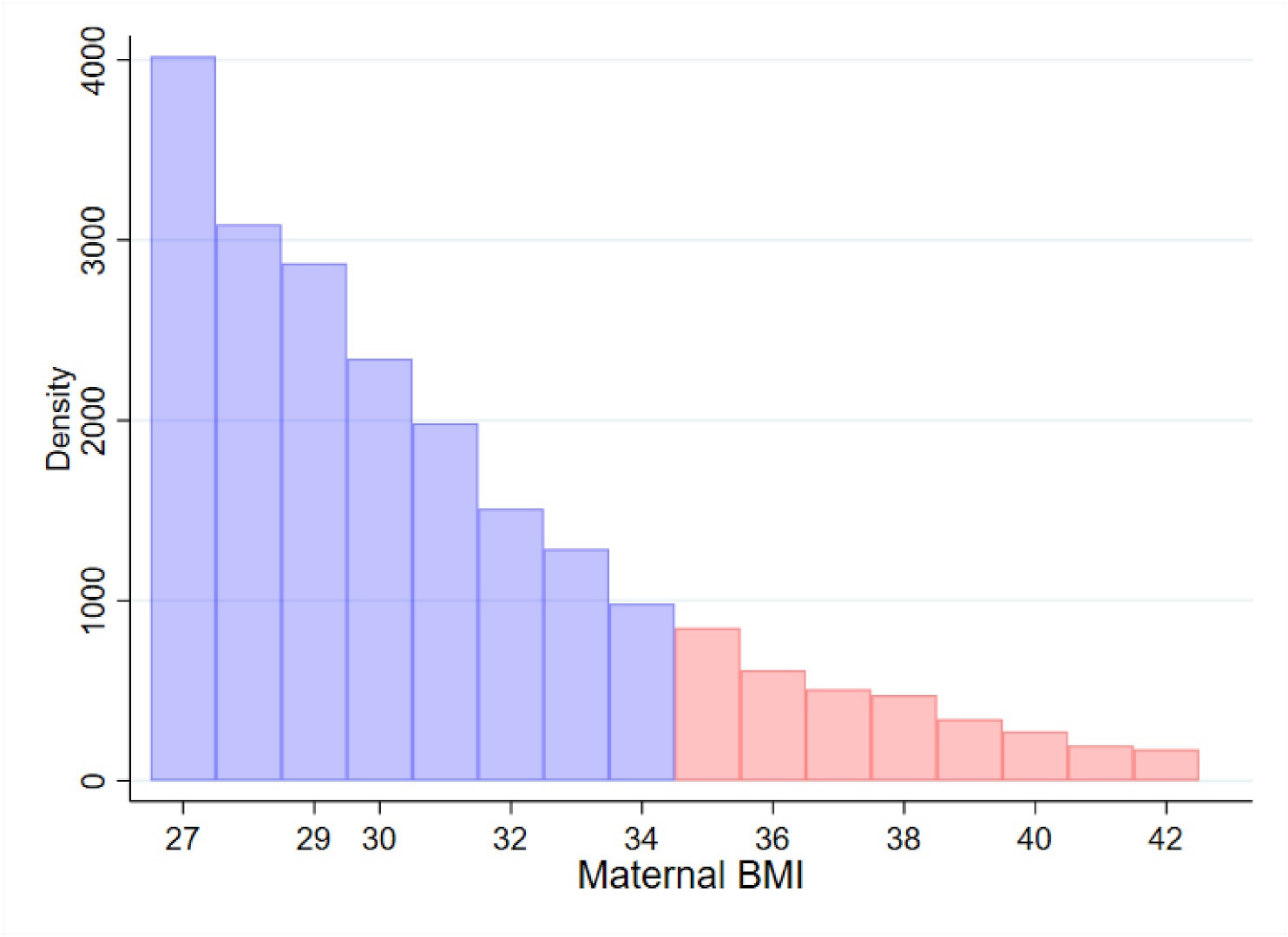
Density of births by maternal BMI. The figure shows the density of births around the 35 maternal BMI threshold. Blue bars indicate that the maternal BMI is below the threshold, red bars that the maternal BMI is above. The sample includes all births in Denmark between 2011 and 2017 that occurred at 7 days after the EDD or later and where the mother had age < 40 and no registry of gestational diabetes. While our maternal BMI data is available up to three digits, we collapsed data at the unity. A more disaggregated figure is presented in Supporting Information S1: Appendix Figure D7. The *p*‐value of the McCrary density test is 0.4554.

Second, the continuity assumption also implies the absence of discontinuity in any other observables or unobservables around the threshold had labor induction at 7 days after the EDD not been implemented. First, we check for any discontinuity in our observable characteristics in Table 1.^23^ This table is overall reassuring for our design. Significant imbalances are rare and overall small.^24^ Despite the large number of observables included in this table, there are no significant imbalances at the 1% level. Nevertheless, being above the 35 threshold may be associated with a higher chance of being a first‐time mother. This is especially pronounced in the EPDS sample, where we also see a slightly reduced risk of having had a previous large child with birth weight ≥4.5 kg (macrosomia). Thus, we could worry that higher parity mothers are less likely to be allowed to await a spontaneous birth until 7 days after the EDD if they faced complications in prior pregnancies (e.g., prior excessive birth weight) while also having a BMI ≥35. If mothers with a BMI ≥35 are treated differently earlier in pregnancy than similar mothers with a BMI just below the cutoff, this threatens our design.

**TABLE 1:** Balance of the observables.

Sample restriction	All	Nurse visit	EPDS	Mean control group (main sample)
	Sharp	Sharp	Sharp	
Any parent went to college	−0.01	−0.02	0.07	0.45
	(0.030)	(0.041)	(0.055)	
Missing education data	−0.02	−0.02	−0.01	0.09
	(0.018)	(0.021)	(0.024)	
Parental income, monthly (US $)	82.29	−138.22	145.48	5821.93
	(182.518)	(231.404)	(291.962)	
Missing income data	−0.02	−0.03	−0.02	0.06
	(0.016)	(0.017)	(0.019)	
Parents are cohabiting	0.00	0.01	0.04	0.84
	(0.021)	(0.025)	(0.032)	
Missing cohabition data	0.01	0.01	−0.00	0.01
	(0.006)	(0.008)	(0.005)	
Any parent Danish	−0.00	−0.01	−0.02	0.88
	(0.018)	(0.021)	(0.020)	
Missing ethnicity data	0.01	0.01	−0.00	0.01
	(0.006)	(0.008)	(0.005)	
Preeclampsia	0.00	−0.00	−0.01	0.01
	(0.007)	(0.010)	(0.007)	
Insufficient growth	−0.00	−0.00	−0.01	0.01
	(0.006)	(0.009)	(0.010)	
Prior C‐section	−0.01	−0.02	0.02	0.09
	(0.016)	(0.020)	(0.033)	
Prior birth weight ≥ 4.5 kg	0.00	−0.01	−0.03^*^	0.02
	(0.008)	(0.009)	(0.011)	
First‐time mother	0.06^*^	0.07	0.12^*^	0.47
	(0.027)	(0.037)	(0.052)	
Parity data missing	0.00	0.00	0.00^*^	< 0.01
	(0.006)	(0.007)	(0.001)	
Maternal age	−0.01	−0.18	0.24	30.17
	(0.273)	(0.346)	(0.530)	
Missing maternal age data	0.00	0.01	0.00	0.01
	(0.004)	(0.006)	(0.006)	
Missing EPDS	0.01	−0.02	—	0.70
	(0.035)	(0.047)	(.)	
Missing nurses visit data	0.04	—	−0.00	0.37
	(0.041)	(.)	(0.004)	

*Note:* Standard errors are robust and clustered at the hospital‐year level.This table displays sharp RDD estimates which are obtained using our favorite specification with polynomial order of one and an epanechnikov kernel. The bandwidth is manually set to 7 for reasons explained in subsection D.3. “Mean in the control group” refers to the average outcome for births on the left‐side of the bandwidth (Maternal BMI 28–35). The set of controls is described in subsection C.1. Each cell displays the coefficient of a single regression with the observable as the outcome, the BMI as a running variable, and the cutoff at 35. In the sample restriction row, “All births” refer to all births in Denmark between 2011 and 2017 that occurred at 7 days after the EDD or later, whereas “Nurse visits” and “EPDS score” refer to the respective subsets of this sample for which nurse visits from the NHV and mental health data are available.

^*^ *p <* 0.05.

**p < 0.01.

***p < 0.001.

In particular, being above the 35 threshold may in some cases trigger a consultation with an obstetrician during pregnancy, additional (but uncertain) weight estimates of the child, and prophylactic epidural use (see Supporting Information S1: Appendix B for details). While the latter health policy does not impose a threat to the continuity assumption, it could make us unable to disentangle the impact of early induction from the impact of increased likelihood of epidural use which instead imposes a threat to the exclusion restriction.

We address whether our results are driven by other health policies at the 35 cutoff (either through selection or as a consequence of epidural use) in Section 6, by exploiting the fact that other relevant health policies are in place for all high‐BMI mothers and not only those who have a pregnancy going at least 7 days after the EDD.

## 5 Results

### 5.1 Treatment and First Stage

We begin by documenting the effect of the guidelines on early induction (7–9 days after the EDD), which is our first stage. Figure 3 shows a notable jump at the BMI threshold: women with BMI ≥35 experience an increase of around 20% points (p.p) in the risk of being induced early, an effect that is absent in the placebo period when the guidelines were not yet issued. Supporting Information S1: Appendix Table D1 further shows the formal first‐stage for our preferred specification across the three different samples: 15 p.p. in our main and nurse visit samples, and almost 20 p.p in the EPDS score sample. Relative to baseline, this corresponds to a 187%–200% increase. In all cases, our estimates are statistically significant at the 1% significance level. Thus, we have strong first stage.

**FIGURE 3:**
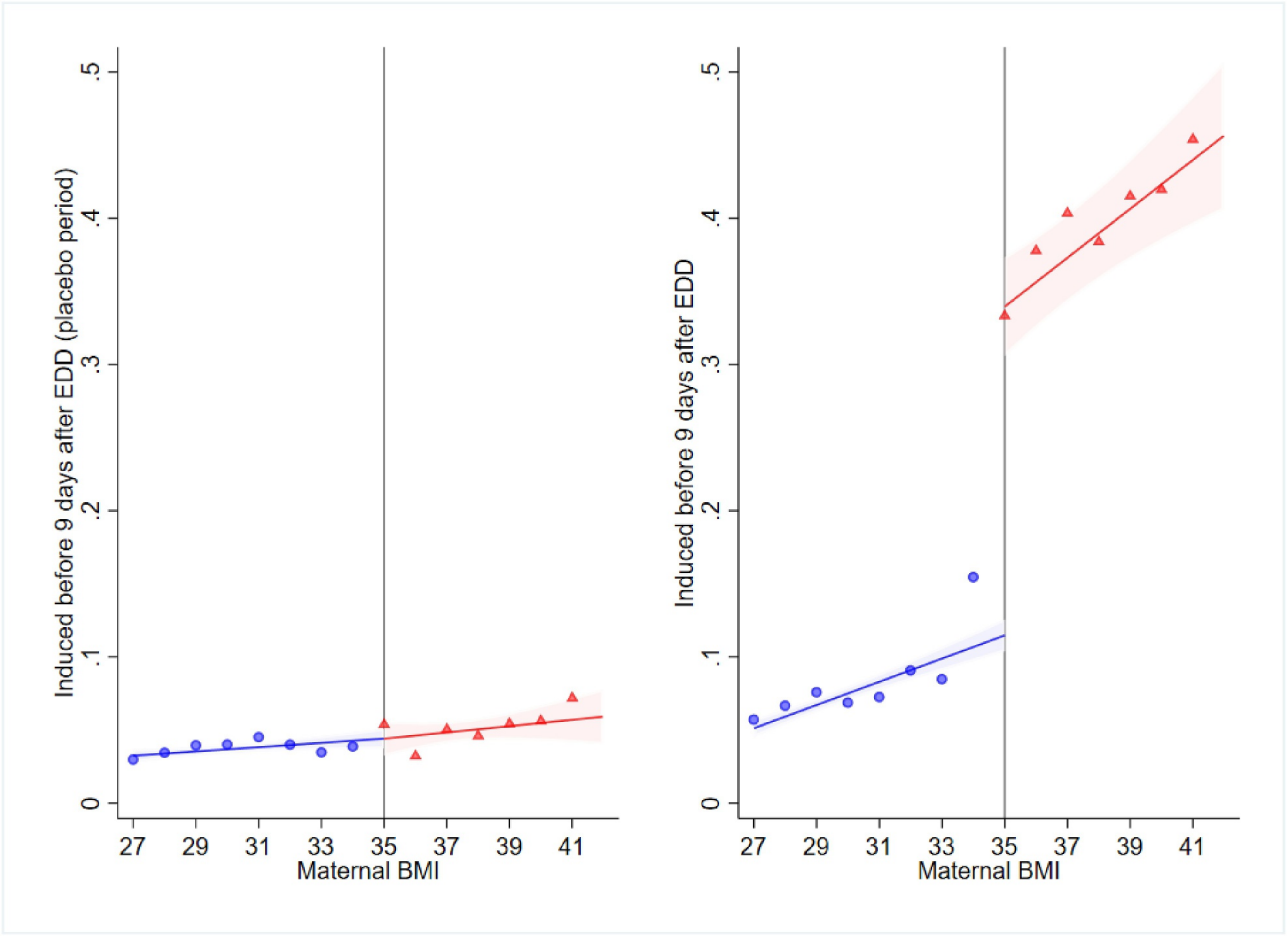
First stage in placebo period (2004–2009) and main sample (2011–2017). The bins are the average outcomes for each integer BMI unit. The lines are fitted lines from an OLS regression on all births where the mother gave birth at least 7 days after the estimated due date (EDD), have an age < 40, and no registry of gestational diabetes. Confidence intervals are at the 90% level and built from heteroskedastic robust SEs clustered at the hospital‐year level. The right panel sample is from the period after the early induction guidelines because of BMI were issued (2011–2017), while the left panel sample is from the placebo period (2004–2009).

Reassuringly, Supporting Information S1: Appendix Figure D1 shows a significant and large first stage estimate across varying bandwidth choices and specifications. Excluding any control variables yields an estimate close to our main results of 15.7 p.p. (results available from the author), and including a second‐order polynomial reduces the estimates to around 11 p.p. For bandwidth choices larger than 7, results on the early induction rate show a tendency to obtain larger estimates that are closer to the 20 p.p increase visualized in Figure 3.

Next, we turn to the two parts of the intervention: a changed mode of labor and a reduced gestational length. Consider a mother with a BMI of 35 following the recommendations of early induction. Had her BMI been below the threshold, she may have had spontaneous onset of labor or she would have been induced anyway, but at a later point in time. In either case, gestational length would likely have been higher.

In Supporting Information S1: Appendix Figure D2, we show that the policy not only shifted the timing of birth but also the overall likelihood of induction. Having a BMI just above the threshold increases the risk of being induced at any time by 8 p.p. (ITT) (see Supporting Information S1: Appendix Table D2). When we address compliance using the fuzzy specification, we find that the probability of ever being induced increased by around 55 p.p. for those who are induced early because they have a BMI close to 35.

Turning to gestational length, Figure 4 shows that early induction reduces pregnancy duration by around 0.7 days (ITT), with no discontinuity in the placebo period. Adjusting for compliance using the fuzzy design implies that pregnancies are shortened by approximately 4 days when they are induced early because their BMI is close to 35 (see Supporting Information S1: Appendix Table D3).

**FIGURE 4:**
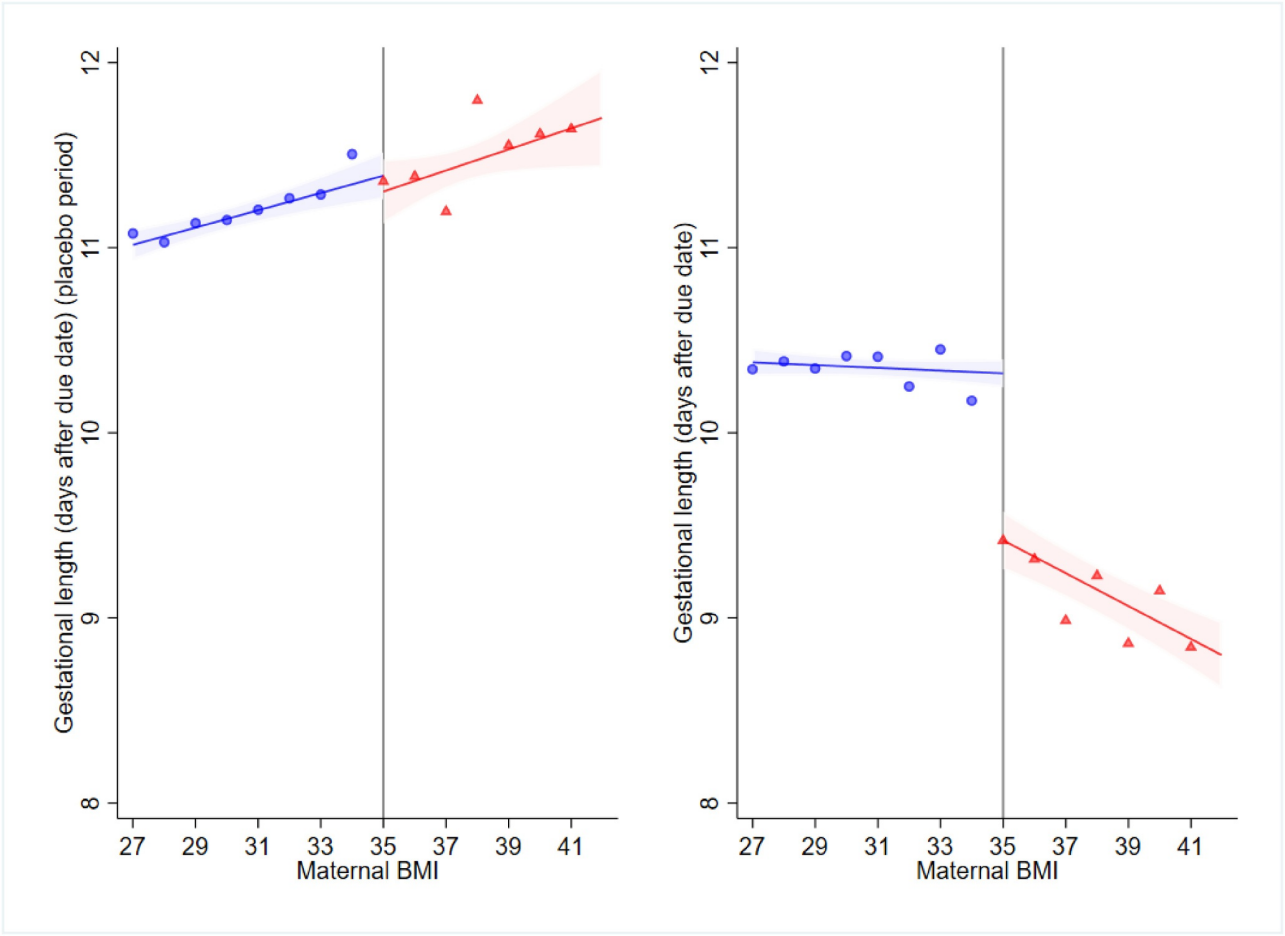
Gestational length (days) in placebo period (2004–2009) and main sample (2011–2017). The bins are the average outcomes for each integer BMI unit. The lines are fitted lines from an OLS regression on all births where the mother gave birth at least 7 days after the estimated due date (EDD), have an age < 40, and no registry of gestational diabetes. Confidence intervals are at the 90% level and built from heteroskedastic robust SEs clustered at the hospital‐year level. The right panel sample is from the period after the early induction guidelines because of BMI were issued (2011–2017), while the left panel sample is from the placebo period (2004–2009).

Our estimates are broadly consistent with previous work but differ in magnitude in intuitive ways. The 8 p.p. (ITT) increase in induction at any time is close to the 10 p.p. (ITT) increase found by Gregersen (2024), while randomized controlled trials (RCTs) typically report larger effects (around 50 p.p. or more), likely reflecting higher compliance in experimental settings (Alkmark et al. 2020; Gelisen et al. 2005; Place et al. 2023). Interestingly, our compliance‐adjusted estimate of around 55 p.p. aligns closely with those findings. Likewise, our compliance‐adjusted estimate on gestational length (≈ 4 days) aligns closely with RCT estimates (≈ 3 days).

### 5.2 Immediate Health and Birth Outcomes

Table 2 presents our main results on immediate health and birth outcomes.^25^ Figure 5 shows the reduced form graphs for cases where we find a statistically significant impact.

**TABLE 2:** Results on immediate health and birth outcomes.

	Sharp	Fuzzy	Obs	Mean control group	RW *p*‐value (fuzzy spec.)
Epidural	0.059^**^	0.335^**^	17317	0.48	0.0594
	(0.028)	(0.138)			
Emergency C‐section	0.000	−0.005	17317	0.06	> 0.999
	(0.012)	(0.057)			
Vacuum extraction	−0.001	0.002	17317	0.08	> 0.999
	(0.015)	(0.070)			
Birth weight (grams)	−45.511^*^	−272.538^**^	17317	3845.75	0.0594
	(23.442)	(111.823)			
Birthweight ≥ 4.5 kg	−0.024^*^	−0.149^**^	17317	0.08	0.1386
	(0.015)	(0.070)			
Shoulder dystocia	−0.006^*^	−0.035^**^	17317	< 0.01	0.1386
	(0.003)	(0.017)			
Lacerations	−0.048^**^	−0.277^**^	17317	0.30	0.0594
	(0.024)	(0.111)			
Lacerations (3rd degree and higher)	0.000	−0.003	17317	0.03	> 0.999
	(0.009)	(0.043)			
Hemorrhage	−0.066^**^	−0.338^***^	12299	0.57	0.0099
	(0.027)	(0.110)			
Complications index	−0.003	−0.017	17317	< 0.01	0.9208
	(0.004)	(0.019)			
Uterus rupture	−0.001	−0.007	17317	< 0.01	0.9802
	(0.003)	(0.017)			
Share sent in NICU	0.004	0.022	17317	0.06	0.9802
	(0.014)	(0.065)			
Apgar < 7	0.002	0.014	17317	< 0.01	0.9802
	(0.005)	(0.023)			
Hospital nights	0.082	0.437	17317	2.21	0.9208
	(0.108)	(0.515)			
Covariates	Yes	Yes			

*Note:* Standard errors are robust and clustered at the hospital‐year level. This table displays RDD estimates which are obtained using our favorite specification with polynomial order of one and epanechnikov kernel. The bandwidth is manually set to 7 for reasons explained in Subsection D.3. Coefficient plots showing the robustness of significant results to varying bandwidths, polynomial order, and kernel can be found in Supporting Information S1: Appendix Figure D10. Supporting Information S1: Appendix Table D6 reports the estimates across various specifications. “Mean in the control group” refers to the average outcome for births on the left‐side of the bandwidth (Maternal BMI 28–35). The set of controls is described in Sub‐Section C.1. For the hemorrhage outcome, the number of observations is lower than for the other outcomes because we drop births before 2013 to account for a change in the definition of hemorrhage in 2012. Last column reports the Romano‐Wolf *p*‐value for the fuzzy RD specification, computed with all outcomes in the table.

^*^ *p* < 0.10.

^**^ *p* < 0.05.

^***^ *p* < 0.01.

**FIGURE 5:**
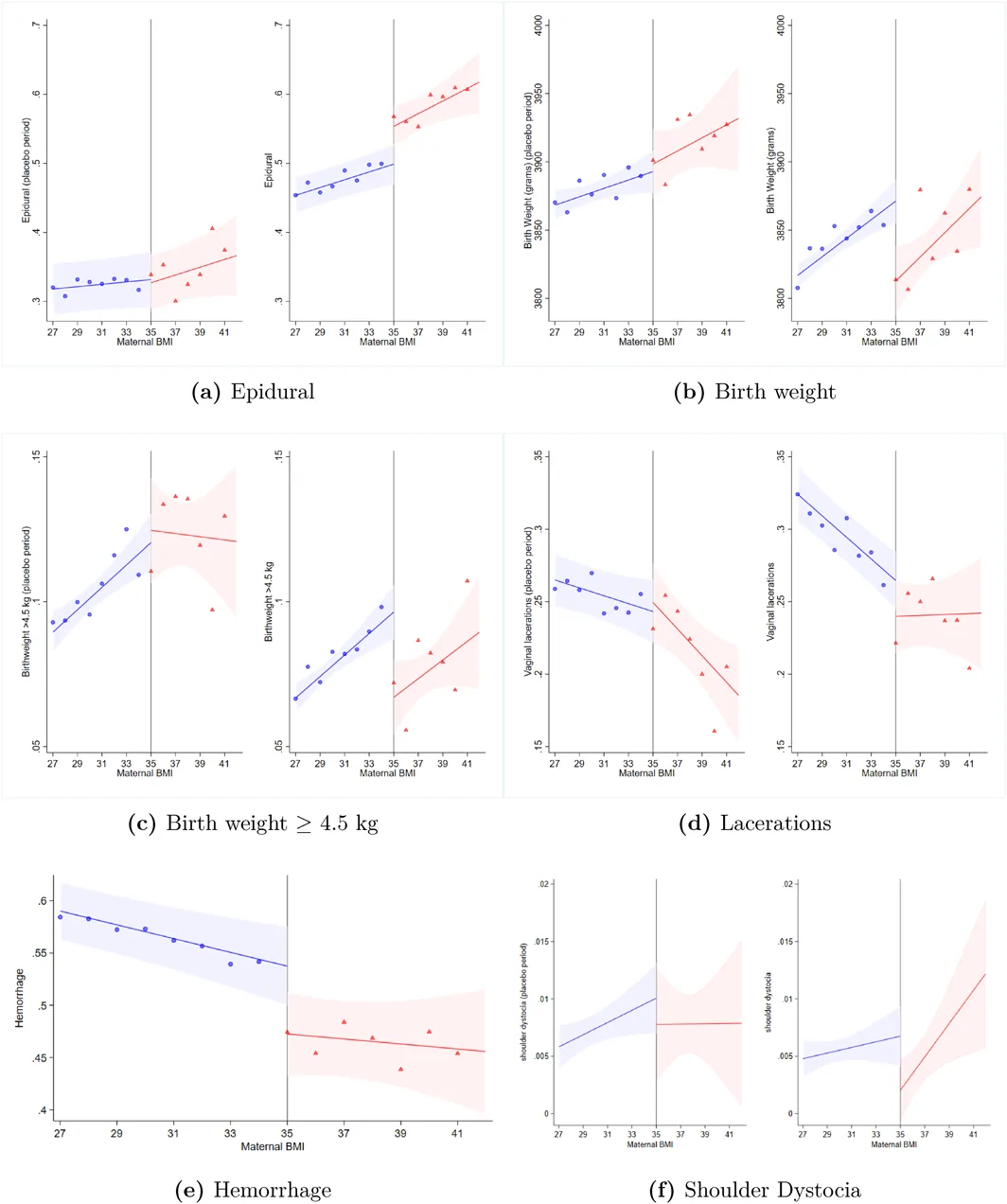
Discontinuity in certain immediate health outcomes. Bins are average outcomes for each integer BMI unit and are only plotted provided a minimum number of obs. Lines are fitted lines from an OLS regression on all births where the mother gave birth at least 7 days after the estimated due date (EDD), have an age < 40, and no registry of gestational diabetes. Confidence intervals are at the 90% level and built from heteroskedastic robust SEs clustered at the hospital‐year level The right panel sample is from the period after the early induction guidelines because of BMI were issued (2011–2017), while the left panel sample is from the placebo period (2004–2009). For hemorrhage, placebo period is not shown because outcome definition changed in 2012, and results are estimated only for the period 2013–2017.

We find no effect of early induction on delivery mode: the impacts on vacuum extraction and emergency C‐section are small and statistically insignificant. Having a BMI ≥ 35 increases epidural use by around 6 p.p. However, part of this effect may stem from separate guidelines on epidural use, as discussed in Section 4.2. We address this issue in Section 6.

Next, we examine the impact of early induction on birth weight. We find that being above the BMI threshold results in an average birth weight reduction of 45 g and a 2.4 p.p. reduced risk of macrosomia, defined here as a birth weight exceeding 4.5 kg. Although the Romano‐Wolf adjusted *p*‐value exceeds the conventional 10% threshold (*p* = 0.13), the discontinuity is clearly visible in Figure 5. We therefore interpret the non‐rejection of the null as likely reflecting limited statistical power (see Section D.3). The discontinuity in birth weight is clearly visible graphically and absent in the placebo period, as shown on (Figure 5, panel b). Our Fuzzy RD estimates imply that women induced early due to a BMI slightly above 35 experience a 15 p.p. reduction in macrosomia risk and an average reduction in birth weight of 272 g. For these women, the birth weight effects are large.

Interestingly, we also detect reductions in several complications potentially influenced by a birth weight ≥ 4.5 kg^26^ including lacerations (broad definition), hemorrhage (broad definition) and shoulder dystocia, a childbirth emergency where the baby's shoulder gets stuck behind the mother's pelvic bone after the head is delivered. While our findings on lacerations and hemorrhage may reflect rather mild incidences, shoulder dystocia is a severe outcome and significant in our analysis. Women induced early due to a BMI just above 35 experience a reduction in shoulder dystocia risk of 3.5 p.p., which is substantial relative to the baseline mean below 1%. However, the corresponding plot is noisy and the result is sensitive to multiple hypothesis testing (the Romano Wolf *p*‐value equals 0.13). Since the outcome is very rare, this may simply be a power issue, but it still makes this result more suggestive.

Another potential complication of having a high birth weight (or shoulder dystocia) is uterus rupture. However, the risk of uterus rupture may also increase due to the induction process too, leading to an ambiguous predicted effect. Overall, we find no statistically significant impact but observe a consistently negative coefficient.

Shoulder dystocia can cause low fetal oxygen levels, which we cannot measure directly but may manifest as a low 5‐min Apgar score. However, the predicted effect on having an Apgar score < 7 at 5 minutes is ambiguous, as it could also be influenced by the induction process itself. Similarly, we find no effect on the risk of having an Apgar < 7 at 5 minutes, but the coefficient is small and positive.

Turning to more severe health outcomes, we find no significant reduction in our measure of severe complications (a dummy capturing either stillbirth, infant mortality or Apgar score < 5 at 5 minutes), yet the coefficients are negative. The primary motivation for advancing the recommended time for routine induction was to reduce the risk of stillbirth; however, this outcome is too rare to analyze independently. Caution is needed with outcomes so rare, as it is challenging to draw statistically sound conclusions on those. While exercising caution, our results on the severe complication index may tentatively suggest a reduced mortality risk. Yet, no significant effects are detected on mothers' hospital nights or NICU admissions.

#### 5.2.1 Comparison With the Existing Literature

The absence of effects on delivery mode, admission to the NICU and the risk of having a low 5‐min Apgar score is consistent with most prior studies (detailed in Supporting Information S1: Appendix A). The increase in epidural use is larger than in previous studies (Alkmark et al. 2020), sometimes even concluded to have no impact (Gregersen 2024; Place et al. 2023). Yet, it may reflect overlapping guidelines, as discussed earlier.

Our estimated reduction in macrosomia risk of (2.4 p.p.) closely matches ITT estimates from RCTs summarized in Alkmark et al. (2020) (−2.78 p.p.), but is considerably larger than the estimate reported by Gregersen (2024) (−0.29 p.p). For birth weight, our ITT estimate (−45 g) lies at the lower range of existing RCTs estimates (−59 to −125 g) (Place et al. 2023; Alkmark et al. 2020; Gelisen et al. 2005), while again exceeding the estimate found by Gregersen (2024) (−10 g).

We also detect reductions in hemorrhage, lacerations, and shoulder dystocia. For low risk populations, the evidence on these outcomes is more mixed: many studies find no impact, while a few RCTs find improvements.^27^ Our findings are in line with a meta‐analysis of induction for suspected macrosomia: they report that induction before the EDD reduces average birth weight by around 178 g, as well as the risk of shoulder dystocia and fracture (Boulvain et al. 2016). This supports the interpretation that avoiding excessive fetal growth may be a key mechanism behind the improvement we observe.

Finally, although most studies lack statistical power to detect effects on mortality and thus should be interpreted with caution, the numbers typically suggest reductions (see Supporting Information S1: Appendix A for details). This pattern is consistent with our results.

### 5.3 Short‐Term Health (Mental Health, GP and Nurse Visits, Social Outcomes)

In this section, we examine whether the immediate benefits extend beyond birth. Table 3 summarizes our results, while Figures 6, 7 present the reduced form results graphically for significant outcomes. All significant regression results remain robust at least at the 10% level when adjusting for multiple hypothesis testing using Romano‐Wolff *p*‐values.

**TABLE 3:** Short‐term outcomes.

	Sharp	Fuzzy	Obs	Mean control group	RW *p*‐value (fuzzy spec.)
EPDS > 11	−0.048^*^	−0.220^*^	5233	0.062	0.1188
	(0.025)	(0.112)			
EPDS score (0–30)	−0.758^*^	−3.514^**^	5233	4.957	0.1188
	(0.395)	(1.787)			
Nurses visit (total)	−0.392^*^	−2.351^**^	11025	6.258	0.0891
	(0.210)	(1.136)			
Universal nurses visit	−0.422^***^	−2.534^***^	11025	4.786	0.0099
	(0.132)	(0.756)			
Nurses, additional visits	−0.029	−0.149	11025	1.494	0.9901
	(0.186)	(0.973)			
Child hosp. contacts (first year)	0.004	0.024	17317	0.697	0.9901
	(0.025)	(0.120)			
Maternal hosp. contacts (first year)	−0.007	−0.045	17317	0.560	0.9901
	(0.029)	(0.139)			
GP contacts (child)	−0.208	−1.518	17317	17.764	0.9406
	(0.502)	(2.421)			
Share employed (mother, 1 year post birth)	0.029	0.190	11577	0.840	0.4950
	(0.023)	(0.141)			
Share employed (mother, 2 years post birth)	0.021	0.113	11577	0.849	0.9406
	(0.020)	(0.126)			
Prob. birth 2 years after	−0.011	−0.077	12072	0.034	0.4950
	(0.012)	(0.072)			
Covariates	Yes	Yes			

*Note:* Standard errors are robust and clustered at the hospital‐year level. This table displays RDD estimates which are obtained using our favorite specification with polynomial order of one and an epanechnikov kernel. The bandwidth is manually set to 7 for reasons explained in Sub‐section D.3. “Mean in the control group” refers to the average outcome for births on the left‐side of the bandwidth (Maternal BMI 28–35). The set of controls is described in Sub‐section C.1. Coefficient plots showing the robustness of significant results to varying bandwidths, polynomial order, and kernel can be found in Supporting Information S1: Appendix Figure D11. Supporting Information S1: Appendix Table D7 reports the estimates across various specifications. The number of observations varies across outcomes because of data availability and because for social outcomes variables 2 years after birth, the years 2016 and 2017 were dropped. Last column reports the Romano‐Wolf *p*‐value for the fuzzy RD specification, computed with all outcomes in the table.

^*^ *p* < 0.10.

^**^ *p* < 0.05.

^***^ *p* < 0.01.

**FIGURE 6:**
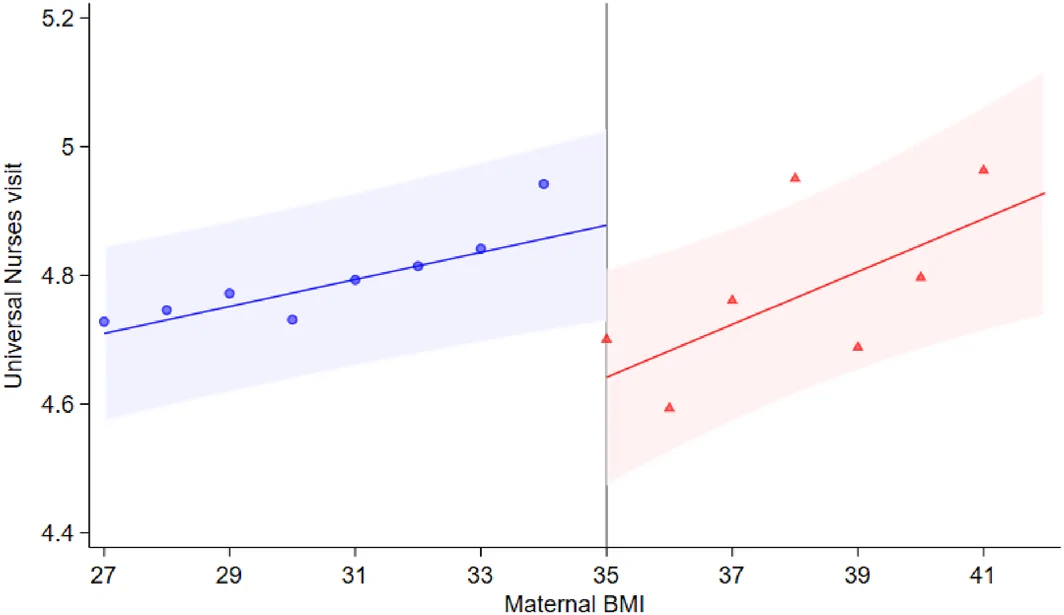
Discontinuity in the number of universal nurse visits (first year after birth). The bins are the average outcomes for each integer BMI unit. The lines are fitted lines from an OLS regression on all births between 2011 and 2017 where the mother gave birth at least 7 days after the estimated due date (EDD), have an age < 40, and no registry of gestational diabetes as well as being a part of the Nurse visit sample. Confidence intervals are at the 90% level and built from heteroskedastic robust SEs clustered at the hospital‐year level. No placebo period is shown because the data from the NHV is not available before 2011.

**FIGURE 7:**
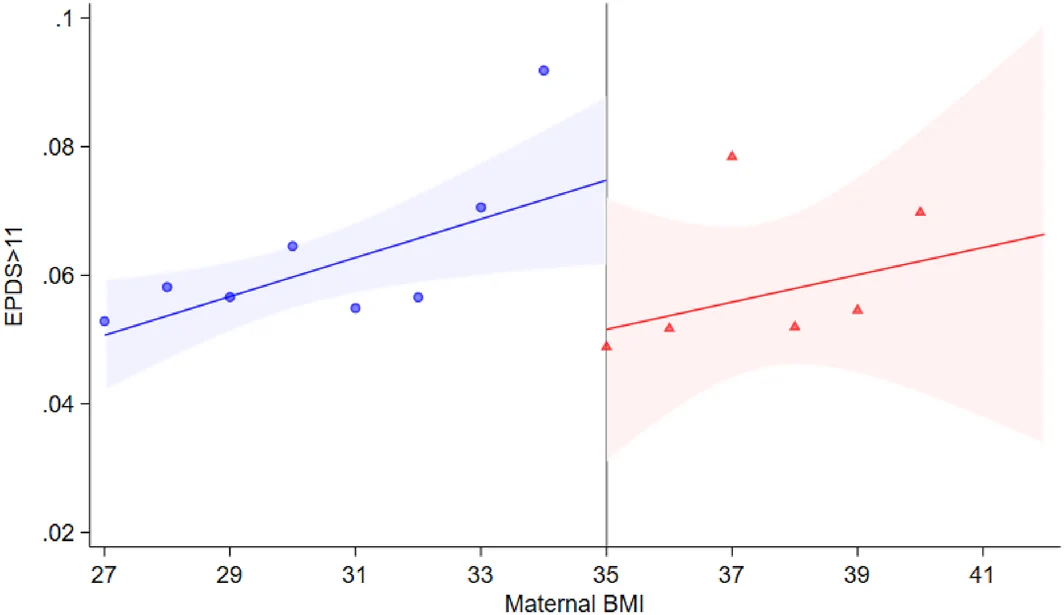
Discontinuity in depression risks (EPDS score > 11). The bins are the average outcomes for each integer BMI unit. The lines are fitted lines from an OLS regression on all births between 2011 and 2017 where the mother gave birth at least 7 days after the estimated due date (EDD), have an age < 40, and no registry of gestational diabetes as well as information on EPDS score. Confidence intervals are at the 90% level and built from heteroskedastic robust SEs clustered at the hospital‐year level. No placebo period is shown because the data from the NHV is not available before 2011. Supporting Information S1: Appendix Figure D4 presents the same figure using the continuous EPDS score as the outcome, although this approach may be less psychometrically appropriate.

We find evidence of reduced healthcare usage during the first year of life following early induction. Specifically, the number of nurse visits (both total and universal) decreased by around two visits (fuzzy estimate) during the first year. While the result on *total* nurse visits is only marginally significant in the regression and less clearly visible in the graphical analysis (see Supporting Information S1: Appendix Figure D3), the effect on *universal* visits is highly statistically significant, stable across specifications (see Appendix coefficient plot D.11(d)), and clearly visible in Figure 6. Additional nurse visits‐those scheduled when extra care is needed—show small and statistically insignificant estimates, but negative coefficients.

Our results on remaining health care usage speak against an interpretation that the reduced (universal) nurse visits reflects a substitution towards more intensive care. GP contacts during the first year show statistically insignificant results, but a negative coefficient (fuzzy estimate: −1.5 GP contacts), with the graphical evidence showing a suggestive drop too (see Supporting Information S1: Appendix Figure D5). For both indicators of having contact with the hospital, we find small and statistically insignificant results. Overall, our findings suggest a genuine reduction in health care usage during the first year of life, rather than substitution toward more intensive services. We do not interpret this reduction as reflecting disengagement from care or reduced access, but rather as a lower need for follow‐up care, consistent with the evidence of improved immediate health in Section 5.2.

The impact on nurse visits only may reflect milder health issues or concerns relative to contacts with GPs and hospitals. In many cases, nurses are typically contacted prior to the GP or hospitals (i.e., to schedule additional visits because of concerns regarding the child's development, problems with breastfeeding etc.). By contrast, GPs need to be contacted in more severe cases requiring prescription medication or specialist referrals. Mothers are generally only able to contact hospitals directly close to birth without any referral. Although the Danish nurse home visiting program is universal, the realized number of (universal) visits depend on coordination between nurses and mothers, as well as on nurses' assessment of family needs and mothers' availability and or preferences. For example, if the nurse assesses that the mother and child are doing well, and the mother perceives a given visit redundant, the nurse may consider that an additional visit is not strictly necessary and may instead prioritize visits for families with greater needs.

Turning to maternal mental health, we find that early induction leads to improvements. The likelihood of having a high EPDS score (> 11) falls by around 5 p.p. (ITT), and the overall EPDS score reduces by approximately 0.8 points corresponding to roughly 20% of a standard deviation (ITT). Mothers induced early due to having a BMI just above the threshold show a substantial reduction relative to the baseline: a 22 p.p. reduced risk of having a high EPDS score and an average reduction of around 3.5 points in the EPDS score—see the results of the fuzzy specifications. The discontinuity is visually evident (see Figure 7), although the effect is not robust to multiple testing (RW pvalue = 0.188), making it suggestive.

Improved maternal mental health may help explain the reduction in nurse visits: mothers with improved mental health may require less support from nurses. If mothers experience better mental health and fewer concerns, both mothers and nurses may find fewer follow‐up visits necessary. Consistent with this interpretation, the effect on the number of both total and universal nurse visits is particularly strong in the subsample with EPDS data (see Supporting Information S1: Appendix Table D4), where the effect on additional nurse visits also becomes negative.

Finally, we find no evidence on any effect on social outcomes such as labor supply and fertility up to 2 years after birth.

#### 5.3.1 Comparison With Existing Literature

Our findings on reduced healthcare usage are consistent with the interpretation that improved maternal and infant health lowers demand for postnatal care, although direct evidence on this margin is limited in the literature. We interpret the fact that we only see a significant impact on nurse visits as consistent with milder improvements in physical health or driven by improved maternal mental health.

The suggested improvement in maternal mental health contrasts with observational evidence suggesting negative associations between induction and postpartum well‐being (see Supporting Information S1: Appendix A). However, evidence from European settings points to heterogeneous preferences over induction timing. While Heimstad, Romundstad, et al. (2007); Heimstad, Skogvoll, et al. (2007) find that many women preferred induction rather than continued monitoring beyond 41 weeks in an RCT, Keulen et al. (2021) document substantial variation in preferences, with a sizable share favoring expectant management. In a small Danish qualitative study based on interviews from 2018, all women had apriori hoped for spontaneous labor instead of induction (Lou et al. 2021).

One potential explanation is the mothers' birth experience, which is associated with postpartum mental health (Waldenström et al. 2004; Simpson and Catling 2016; Thomson and Downe 2010). While qualitative evidence suggests that induction may reduce perceived control during labor (Lou et al. 2019), our findings indicate that improvement in immediate health outcomes may offset such effects and, on average, improve maternal well‐being.

Finally, our null results on fertility and labor supply are consistent with Gregersen (2024), who also finds no effects of earlier routine induction on fertility. This contrasts with evidence showing that avoidable C‐sections can reduce fertility and affect labor supply (Halla et al. 2020), suggesting that the mechanism we study operates differently.

## 6 Disentangling Early Induction BMI‐Based Guidelines Effects From That of Other BMI‐Based Guidelines

The goal of this section is to disentangle the impact of the early induction guidelines from that of other guidelines potentially affecting mothers with a BMI just above 35.

In general, overweight mothers are advised to lose weight prior to becoming pregnant and to reduce their weight gain during pregnancy (see Supporting Information S1: Appendix B for detailed information on overweight pregnancies in Denmark). Thus, on the one hand, healthcare professionals may not substantially change behavior towards mothers with a BMI just above the 35 threshold which speaks in favor of our continuity assumption. Nevertheless, some hospitals schedule a talk with an obstetrician during pregnancy and additional checks focusing on estimating the child's size when a mother's BMI is above the exact 35 threshold. Moreover, epidural may be used prophylactically for women above this threshold too. Thus, we need to rule out that any differences in health outcomes are explained by these other types of health policies.^28^

The additional checks during pregnancy may impose a threat to the continuity assumption if they lead to the discovery of underlying health problems necessitating a birth prior to 7 days after the EDD. In that case, women with BMI of at least 35 included in our main sample would be positively selected relative to women just below the cutoff. Increased epidural prophylactic is not a concern for the continuity assumption, but it may make it impossible to disentangle potential health effects stemming from increased epidural use from health effects stemming from the early induction policy thereby invalidating the exclusion restriction.

In order to address these concerns, we exploit the fact that these other health policies effective at the 35 threshold are in place for all pregnancies and not just the ones who pass 7 days after the EDD. We construct a placebo sample similar to our main sample except for birth timing: we now include all pregnancies giving birth prior to 7 days after the EDD (including those before the EDD). If other policies at the BMI cutoff would lead to a selection out of our main sample, we would expect to see a higher density of observations in this placebo sample to the right of the threshold. Supporting Information S1: Appendix Figures D8 and D9 show no such pattern.

We could still worry that additional checks at the cutoff lead to a differential treatment during pregnancy which in turn impacts health outcomes without affecting the birth timing. This would mean that early induction is not the only mechanism behind our results. To address this concern, we repeat our main analysis on variables which were significant in the sharp specification for our placebo sample. The idea is that if any of our results are not driven by the early induction policy, but instead different treatment during pregnancy, our outcomes should be affected significantly in the same direction for this alternative sample.

If our results are driven by positive selection, then, in this placebo sample, we would instead expect mothers above the threshold to have worse health outcomes. We additionally study differences in elective C‐section rates to further address one potential source of possible positive selection of women with a BMI above the threshold into our sample: talking with an obstetrician during pregnancy could lead to a higher risk of elective C‐sections because of the discovery of underlying health issues. An elective C‐section typically takes place prior to the EDD and such women would thus not be a part of our main sample.

Table 4 below shows the reduced form (sharp) estimates for all outcomes which we found were significantly impacted by earlier routine induction for high‐BMI women, together with results on elective C‐section. While the coefficient on elective C‐section is positive, it is small and highly statistically insignificant. Thus, we do not find evidence of a positive selection at the exact 35 threshold in our main sample driven by talking with an obstetrician during pregnancy (and then leading to a higher risk of having an elective C‐section prior to 7 days after the EDD).

**TABLE 4:** Placebo checks.

	Sharp	Sharp	Obs (placebo/main sample)
Elective CS	0.010	—	45764/17317
	(0.010)	—	
Macrosomia	0.005	−0.024^*^	45764/17317
	(0.006)	(0.015)	
Birth weight (grams)	−1.759	−45.511^*^	45764/17317
	(20.546)	(23.442)	
Epidural	0.019	0.059^**^	45764/17317
	(0.019)	(0.028)	
Gestational length (Days)	−0.304	−0.678^***^	45764/17317
	(0.515)	(0.149)	
Shoulder dystocia	−0.000	−0.006^*^	45764/17317
	(0.002)	(0.003)	
Hemorrhage	0.002	−0.066^**^	32510/12299
	(0.017)	(0.024)	
Lacerations	−0.018	−0.048^**^	45764/17317
	(0.015)	(0.024)	
Nurses visit (total)	0.066	−0.392^*^	28743/11025
	(0.154)	(0.210)	
Universal nurses visit	0.010	−0.422^***^	28743/11025
	(0.094)	(0.132)	
EPDS > 11	−0.030^*^	−0.048^*^	12577/5233
	(0.016)	(0.025)	
EPDS score	−0.022	−0.758^*^	12577/5233
	(0.250)	(0.395)	
Days after EDD	< 7	≥7	
Sample	(Placebo)	(Main sample)	
Covariates	Yes	Yes	

*Note:* This table shows effects for those affected by the early induction guidelines (main sample) versus those not (placebo). The effects displayed are sharp RDD estimates which are obtained using our favorite specification with polynomial order of one and an epanechnikov kernel. The bandwidth is manually set to 7 for reasons explained in Sub‐section D.3. The set of controls is similar to that described Sub‐section C.1, except that for the placebo sample preeclampsia, and insufficient growth (recorded before the EDD) are excluded from the specification to avoid bad controls issues. The number of observations varies across outcomes because of data availability. The elective C‐section coefficient is not shown in the main sample as it is only used as an additional placebo check (if there is no selection into our main sample at the cutoff, we would not expect any differences in the likelihood of having an elective C‐section prior to passing 7 days after the EDD). Standard errors are robust and clustered at the hospital‐year level.

^*^ *p* < 0.10.

^**^ *p* < 0.05.

^***^ *p* < 0.01.

Reassuringly, none of the affected outcomes from our main analysis is statistically affected at the 5% level in the sample of mothers who give birth earlier than 7 days after the EDD suggesting that our results are not driven by other health policies taking place at the 35 threshold, not even using epidural prophylactic because of BMI. Moreover, the magnitudes of the estimated coefficients for epidural, gestational length (past the EDD), birth weight, shoulder dystocia, EPDS score and nurse visits (both total and universal) are all much smaller than the ones we found in our main result section. Somewhat worryingly, the impact of induction on the share of mothers with EPDS > 11 is significant at the *p* < 0.1 level in both the placebo and main samples. Still, the continuous EPDS score is not significant in the placebo sample and significant at the 10% level in the main sample. Reassuringly, for both EPDS measures, the effect sizes are bigger in the main sample than in the placebo sample. For the continuous score, the main‐sample estimate is roughly thirty‐five times the placebo counterpart, reinforcing confidence that the main‐sample findings reflect a genuine treatment effect rather than residual bias.

Finally, to further study potential selection into the sample through increased discoveries of pregnancy complications, we add Supporting Information S1: Appendix Table D8 which examines outcomes more directly related to conditions that could justify earlier delivery. Specifically, we study discontinuities at the threshold in preeclampsia, gestational diabetes, earlier induction, and insufficient fetal growth.^29^ For this exercise, we use a broader sample than in our main specification. In particular, while our baseline sample excludes mothers with gestational diabetes diagnosed before the EDD, they must be included here in order to test whether the probability of a gestational diabetes diagnosis itself changes discontinuously at the cutoff.

Overall, the results provide little evidence of systematic differences in pregnancy complications or early intervention at BMI 35. We do not detect discontinuities in preeclampsia, gestational diabetes, or earlier induction, insufficient growth at the 5% level.^30^ Taken together, these results do not suggest a systematic mechanism whereby women just above the threshold are more intensively monitored, diagnosed with complications, and delivered earlier in a way that would threaten the interpretation of the design. If such a mechanism were important, we would expect to observe clear and consistent discontinuities across the broader set of pregnancy complications considered.

## 7 Conclusion

The number of pre‐pregnancy overweight and obese women is rising in many countries. While pre‐pregnancy BMI is correlated with adverse birth outcomes for mothers and children, little causal evidence exists on which health policies based on pre‐pregnancy BMI are beneficial. We study the effect of Danish obstetric guidelines recommending early routine induction for mothers with a pre‐pregnancy BMI of at least 35 (7 days after their expected due date instead of 10–13 days after) using a regression discontinuity design. Our institutional setting implies that we only consider high‐BMI women with no other major comorbidity. This is an interesting setting to study the impact of a policy based solely on pre‐pregnancy BMI.

Our findings are threefold. First, early induction for high‐BMI women improves immediate and first‐year health outcomes. It reduces the share of newborns with birth weight of at least 4.5 kg. Consistently, the intervention reduces the occurrence of certain neonatal complications which can be triggered by a too‐high birth weight: hemorrhage, vaginal lacerations, and shoulder dystocia (suggestive). The main argument favoring early induction is a reduced mortality risk. In line with the medical literature, we find no significant reduction in our measure of severe complications (a dummy capturing either stillbirth, infant mortality or Apgar score < 5 at 5 minutes), although the coefficients are negative. Caution is however needed with outcomes so rare.

Our second main result is evidence that the benefits of early induction for high‐BMI mothers may persist beyond birth. Specifically, we find suggestive evidence that healthcare usage is lower during the first year of life for early induced mothers, and that the intervention reduces maternal postpartum depression risks. However, our third and final set of results shows no impact on mothers' labor supply and/or fertility responses the first 2 years after birth.

The overall beneficial health effects contrast with the more mixed evidence found for lower‐risk pregnancies. Thus, our study provides causal evidence of beneficial effects of providing early induction for mothers with a BMI close to 35. Although we are unable to conduct a formal cost‐benefit analysis, the observed health improvements suggest that a targeted induction policy for high‐BMI women may be more cost‐effective than a universal approach.^31^ Nevertheless, we leave a formal cost‐benefit analysis for future research. The fact that we find beneficial health effects for high‐BMI mothers is an important policy result, given the growing number of obese mothers‐to‐be and the broader debate surrounding the medicalization of childbirth.

## Conflicts of Interest

The authors declare no conflicts of interest.

## Supporting information


Supporting Information S1

## Data Availability

This study uses administrative registers from Statistics Denmark and municipal health data approved by the Danish Patient Safety Authority (approval 3‐3013‐2507/1). Analyses were conducted on secure servers hosted by Statistics Denmark. Due to legal and institutional restrictions, these data cannot be made publicly available. However, access may be granted to other researchers through application to Statistics Denmark and relevant municipal authorities. We are happy to provide guidance on the application process upon request. We therefore request an exemption from the data availability requirement.

## Endnotes

^1^
One in every fourth birth at term was induced in developed countries in 2011 (WHO 2011).

^2^
See Gregersen (2024) for a review on the discussion on early induction in general.

^3^
Globally, Chen et al. (2018) estimate that in 2014, 38.9 million pregnancies involved overweight mothers and 14.6 million involved obese mothers. While our results cannot be directly extrapolated to broader categories of overweight pregnancies, the rising global prevalence of maternal obesity nevertheless underscores the broader relevance of studying such targeted induction policies.

^4^
Our control group is defined as births on the left‐side of the bandwidth of our main specification (Maternal BMI 28–35).

^5^
For instance, in 2011, in the beginning of our sample period, there were only 245 stillbirths, against 58,999 live births, according to public data from statistics Denmark.

^6^
Gestational length is rather precisely estimated for most women in Denmark during our sample period. It is typically determined using crown‐rump length measurement during the first‐trimester ultrasound scan taking place when assessing the risk for Downs syndrome (Zizzo et al. 2017; DSOG 2015).

^7^
Between 2011 and 2017 in Denmark, 63% of mothers with a BMI greater than or equal to 35 had no other severe risk factor and could therefore have been directly affected by an early‐induction policy.

^8^
Depending on how ready her body is (how ripe the woman's cervix is), different means of inducing labor may be implemented, and in many cases, induced labor will result in a combination of different means. When the woman's cervix is not ripe, the standard induction process typically involves taking a drug for up to 3 days (until labor kicks in or until the body is ripe enough to directly ripe the amniotic sack). If the drug is unsuccessful, the woman will typically have another appointment at the hospital to determine the best action forward (postponing induction, trying another method of induction or planning a C‐section). Sometimes it is possible to rupture the amniotic sack right away. Further down the process, contraction‐stimulating medicine may be necessary if the contractions do not kick in or are not strong enough.

^9^
In principle, the mother could also develop a complication which would have led her to be induced earlier than 10–13 days after the EDD.

^10^
These mild cases of GDM are recommended induction at 7 days after the EDD. Similarly women of at least 40 years of age were recommended induction at 7 days after the EDD. We drop both groups of pregnancies since their recommended induction status does not differ across the BMI cutoff. Guidelines for women with a prior C‐section are less uniform. They are recommended to give birth earlier than 10 days after the EDD. If allowed to be induced (instead of receiving a planned C‐section) their induction procedure differs because of their elevated risk of uterus rupture (DSOG 2011). We control for having had a prior C‐section in our main specification.

^11^
Notice however that it is difficult to correctly predict the size of a large baby, especially for overweight and obese women (DSOG 2007, 2018). See Supporting Information S1: Appendix B for details on antenatal care particularly relevant for high‐BMI mothers.

^12^
While it is standard to be medically induced in many circumstances, we do not capture pure mechanical inductions. Specifically, we will not include inductions of women with a previous C‐section or other major uterus surgery as they are advised only to be mechanically induced because of their increased risk of uterus rupture (DSOG 2014). In 2014, most hospital use the drug misoprostol for labor induction (Hedegaard et al. 2014). Yet, if the woman's body is already ripe, labor may be induced by rupturing the amniotic sack.

^13^
See Supporting Information S1: Appendix B for a detailed description of high‐BMI pregnancies in Denmark and the associated risks—including increased risk of macrosomia (birth weight ≥4.5 kg).

^14^
Results are robust to excluding obstetric high vaginal laceration (O714).

^15^
The Apgar score < 4 at 5 minutes outcome is more common in the medical literature, but to avoid having too few observations to comply with the rules and regulations of Statistics Denmark, we use the cutoff below 5. Using the below 4 cutoff does not change our result.

^16^
This subset is not selected specifically for the present paper. It closely corresponds to the municipalities with EPDS data used in (Bhalotra et al. 2026). Supporting Information S1: Appendix Table B1 in Bhalotra et al. (2026) compares mothers who were screened with the EPDS, and therefore come from the included municipalities, with mothers who were not screened, who come mostly from omitted municipalities. It shows that the two groups are fairly similar in terms of observable characteristics.

^17^
GDM is identified through ICD‐10 code: O24.4 in the LPR.

^18^
Main results are also robust to excluding mothers with prior C‐section who recommended to have given birth before reaching 10 days after the EDD.

^19^
We exclude the year of 2010 to factor out any potential announcement effects. While the early induction guidelines were implemented in 2011, the first draft of the guidelines was published already in 2009 and Zizzo et al. (2017) report that a few hospitals already had an early induction policy for high‐BMI women in 2010. Results are robust to including 2010 in the placebo‐period in any case.

^20^
Large language models (ChatGPT, copilot) were used for proofreading and language editing.

^21^
Constructing our weekday fixed effects based on the expected due date (EDD) instead of the day of birth does not affect our results (available from the author).

^22^
Note that the number of hospitals is too low for clustering on the hospital level to be appropriate. In 2014, only 14 hospitals had a maternity unit (see e.g., Supporting Information S1: Appendix 2 in the following guidelines: DSOG (2015)).

^23^
As our main sample includes pregnancies passing the EDD with 7 days only and multiple pregnancies are recommended given birth before the EDD, the number of women in our sample with a multiple pregnancy is very small. When splitting further on BMI, the number of observations in some cells is too small to comply with the disclosure control rules imposed by our data provider, and we therefore do not include the multiple birth indicator in Table 1.

^24^
This absence of large imbalances in observables is consistent with the fact that our RD estimates remain stable, though sometimes slightly less precise, when the control set is added or removed, as shown in Supporting Information S1: Appendix Tables D6 and D7.

^25^
Although counts for individual immediate outcomes may differ by only a few observations because of missing data, we report a constant sample size to comply with Statistics Denmark's rule against disclosing results for very small populations.

^26^
See Stotland et al. (2004) for more information on the risk factors associated with a birth weight ≥ 4.5 kg (macrosomia).

^27^
Gregersen (2024) find no impact on lacerations (broad definition). Most RCTs report no impact on hemorrhages (Alkmark et al. 2020; Heimstad, Romundstad, et al. 2007), but Place et al. (2023) report a reduction. Wennerholm et al. (2019) find no impact on shoulder dystocia, but Gelisen et al. (2005) report a reduced risk.

^28^
Having a high BMI may restrict access to fertility treatment through the public health care system, but the exact BMI threshold varies across hospitals (between 29 and 40) and across age—and different risk groups too. Thus, the threshold for access to fertility treatment do not coincide with our BMI threshold but exists both above and below the 35 cutoff.

^29^
We also include elective cesarean section as in the earlier analysis, to check if our null result is also found in this slightly broader sample.

^30^
We do find some evidence of a positive discontinuity in insufficient fetal growth around the cut‐off, but the estimate is only marginally significant (*p* < 0.10.)

^31^
All else equal, induced birth is more expensive than awaiting spontaneous labor. In a back‐of‐the‐envelope calculation, Gregersen (2024) reports that the average difference in charges between an uncomplicated induced birth and an uncomplicated spontaneous birth was DKK 3611 ($509.97) in 2022. Our results point to health improvements that would be expected to generate cost savings. Unfortunately, however, we do not have credible cost estimates for the main health improvements documented in this paper.

## References

[hec70143-bib-0001] Alkmark, M. , J. K. Keulen , J. C. Kortekaas , et al. 2020. “Induction of Labour at 41 Weeks or Expectant Management Until 42 Weeks: A Systematic Review and an Individual Participant Data Meta‐Analysis of Randomised Trials.” PLoS Medicine 17, no. 12: e1003436. 10.1371/journal.pmed.1003436. PMC7723286 33290410

[hec70143-bib-0002] Almond, D. , J. J. Doyle Jr , A. E. Kowalski , and H. Williams . 2010. “Estimating Marginal Returns to Medical Care: Evidence From At‐Risk Newborns.” Quarterly Journal of Economics 125, no. 2: 591–634. 10.1162/qjec.2010.125.2.591. PMC2903901 20634927

[hec70143-bib-0003] Baranov, V. , S. Bhalotra , P. Biroli , and J. Maselko . 2020. “Maternal Depression, Women’s Empowerment, and Parental Investment: Evidence From a Randomized Controlled Trial.” American Economic Review 110, no. 3: 824–859. 10.1257/aer.20180511.

[hec70143-bib-0004] Behrman, J. R. , and M. R. Rosenzweig . 2004. “Returns to Birthweight.” Review of Economics and Statistics 86, no. 2: 586–601. 10.1162/003465304323031139.

[hec70143-bib-0005] Bhalotra, S. , N. M. Daysal , L. Freget , et al. 2026. “Postpartum Depression and the Motherhood Penalty.”.

[hec70143-bib-0006] Black, S. E. , P. J. Devereux , and K. G. Salvanes . 2007. “From the Cradle to the Labor Market? The Effect of Birth Weight on Adult Outcomes.” Quarterly Journal of Economics 122, no. 1: 409–439. 10.1162/qjec.122.1.409.

[hec70143-bib-0007] Borra, C. , L. González , and A. Sevilla . 2019. “The Impact of Scheduling Birth Early on Infant Health.” Journal of the European Economic Association 17, no. 1: 30–78. 10.1093/jeea/jvx060.

[hec70143-bib-0008] Boulvain, M. , O. Irion , and J. G. Thornton . 2016. “Induction of Labour at or Near Term for Suspected Fetal Macrosomia.” Cochrane Database of Systematic Reviews 5, no. 8. 10.1002/14651858.cd000938.pub2. PMC7032677 27208913

[hec70143-bib-0009] Bryan, M. L. , N. Rice , J. Roberts , and C. Sechel . 2022. “Mental Health and Employment: A Bounding Approach Using Panel Data.” Oxford Bulletin of Economics & Statistics 84, no. 5: 1018–1051. 10.1111/obes.12489.

[hec70143-bib-0010] Buckles, K. , and M. Guldi . 2017. “Worth the Wait? The Effect of Early Term Birth on Maternal and Infant Health.” Journal of Policy Analysis and Management 36, no. 4: 748–772. 10.1002/pam.22014. 28991421

[hec70143-bib-0011] Calonico, S. , M. D. Cattaneo , M. H. Farrell , and R. Titiunik . 2017. “Rdrobust: Software for Regression‐Discontinuity Designs.” STATA Journal 17, no. 2: 372–404. 10.1177/1536867x1701700208.

[hec70143-bib-0012] Card, D. , A. Fenizia , and D. Silver . 2023. “The Health Impacts of Hospital Delivery Practices.” American Economic Journal: Economic Policy 15, no. 2: 42–81. 10.1257/pol.20210034.

[hec70143-bib-0013] Chen, C. , X. Xu , and Y. Yan . 2018. “Estimated Global Overweight and Obesity Burden in Pregnant Women Based on Panel Data Model.” PLoS One 13, no. 8: e0202183. 10.1371/journal.pone.0202183. PMC6084991 30092099

[hec70143-bib-0014] Clarke, D. , J. P. Romano , and M. Wolf . 2020. “The Romano–Wolf Multiple‐Hypothesis Correction in Stata.” STATA Journal 20, no. 4: 812–843. 10.1177/1536867x20976314.

[hec70143-bib-0015] Coates, D. , A. Makris , C. Catling , et al. 2020. “A Systematic Scoping Review of Clinical Indications for Induction of Labour.” PLoS One 15, no. 1: e0228196. 10.1371/journal.pone.0228196. PMC6988952 31995603

[hec70143-bib-0016] Costa‐Ramón, A. M. , A. Rodríguez‐González , M. Serra‐Burriel , and C. Campillo‐Artero . 2018. “It’s About Time: Cesarean Sections and Neonatal Health.” Journal of Health Economics 59: 46–59. 10.1016/j.jhealeco.2018.03.004. 29673899

[hec70143-bib-0017] Cox, J. , and J. Holden . 2003. Perinatal Mental Health: A Guide to the Edinburgh Postnatal Depression Scale (EPDS). Royal College of Psychiatrists.

[hec70143-bib-0018] Daysal, N. M. , M. Trandafir , and R. Van Ewijk . 2015. “Saving Lives at Birth: The Impact of Home Births on Infant Outcomes.” American Economic Journal: Applied Economics 7, no. 3: 28–50. 10.1257/app.20120359.

[hec70143-bib-0019] DSOG . 2007. “Foetus Magnus Suspicio (DO366).” https://web.archive.org/web/20220120230459/http://gynobsguideline.dk/sandbjerg/100411%20foetusmagnussuspicio%20Sandbjerg%202007.pdf.

[hec70143-bib-0020] DSOG . 2011. “Graviditet Efter Termin.” https://www.dsog.dk/obstetrik.

[hec70143-bib-0021] DSOG . 2014. “Igansœttelse: Af Fødsel.” https://web.archive.org/web/20220120225251/http://static.squarespace.com/static/5467abcce4b056d72594db79/546e7748e4b0d969a4f6cf10/546e7746e4b0d969a4f6cc5a/1395263134000/PP‐med‐2014.pdf?format=original.

[hec70143-bib-0022] DSOG . 2015. Monitorering af Gravide Efter Gestationsalder 41+0. https://www.dsog.dk/obstetrik.

[hec70143-bib-0023] DSOG . 2018. “Foetus Magnus Suspicio (DO366).” https://www.dsog.dk/obstetrik.

[hec70143-bib-0024] Figlio, D. , J. Guryan , K. Karbownik , and J. Roth . 2014. “The Effects of Poor Neonatal Health on Children’s Cognitive Development.” American Economic Review 104, no. 12: 3921–3955. 10.1257/aer.104.12.3921. 29533575

[hec70143-bib-0025] Frank, R. G. and E. Meara . 2009. “The Effect of Maternal Depression and Substance Abuse on Child Human Capital Development.” Technical report, National Bureau of Economic Research.

[hec70143-bib-0026] Gans, J. S. , and A. Leigh . 2009. “Born on the First of July: An (Un) Natural Experiment in Birth Timing.” Journal of Public Economics 93, no. 1–2: 246–263. 10.1016/j.jpubeco.2008.07.004.

[hec70143-bib-0027] Gelisen, O. , E. Caliskan , S. Dilbaz , E. Ozdas , B. Dilbaz , and A. Haberal . 2005. “Induction of Labor With Three Different Techniques at 41 Weeks of Gestation or Spontaneous Follow‐Up Until 42 Weeks in Women With Definitely Unfavorable Cervical Scores.” European Journal of Obstetrics & Gynecology and Reproductive Biology 120, no. 2: 164–169. 10.1016/j.ejogrb.2004.08.013. 15925045

[hec70143-bib-0028] Gregersen, M. K. 2024. “Earlier Routine Induction of Labor—Consequences on Mother and Child Morbidity.” Health Economics 33, no. 10: 2399–2418. 10.1002/hec.4877. 38965767

[hec70143-bib-0029] Halla, M. , H. Mayr , G. J. Pruckner , and P. Garcia‐Gomez . 2020. “Cutting Fertility? Effects of Cesarean Deliveries on Subsequent Fertility and Maternal Labor Supply.” Journal of Health Economics 72: 102325. 10.1016/j.jhealeco.2020.102325. 32535109

[hec70143-bib-0030] Hedegaard, M. , Ø. Lidegaard , C. Skovlund , L. Mørch , and M. Hedegaard . 2015. “Perinatal Outcomes Following an Earlier Post‐Term Labour Induction Policy: A Historical Cohort Study.” BJOG: An International Journal of Obstetrics & Gynaecology 122, no. 10: 1377–1385. 10.1111/1471-0528.13299. 25690911

[hec70143-bib-0031] Hedegaard, M. , Ø. Lidegaard , C. W. Skovlund , L. S. Mørch , and M. Hedegaard . 2014. “Reduction in Stillbirths at Term After New Birth Induction Paradigm: Results of a National Intervention.” BMJ Open 4, no. 8: e005785. 10.1136/bmjopen-2014-005785. PMC4139643 25125480

[hec70143-bib-0032] Heimstad, R. , P. R. Romundstad , J. Hyett , L.‐Å. Mattsson , and K. Å. Salvesen . 2007. “Women’s Experiences and Attitudes Towards Expectant Management and Induction of Labor for Post‐Term Pregnancy.” Acta Obstetricia et Gynecologica Scandinavica 86, no. 8: 950–956. 10.1080/00016340701416929. 17653880

[hec70143-bib-0033] Heimstad, R. , E. Skogvoll , L.‐Å. Mattsson , O. J. Johansen , S. H. Eik‐Nes , and K. Å. Salvesen . 2007. “Induction of Labor or Serial Antenatal Fetal Monitoring in Postterm Pregnancy: A Randomized Controlled Trial.” Obstetrics & Gynecology 109, no. 3: 609–617. 10.1097/01.aog.0000255665.77009.94. 17329511

[hec70143-bib-0034] Hirani, J. L.‐J. , H. H. Sievertsen , and M. Wüst . 2020. Missing a Nurse Visit. Technical report: IZA Discussion Papers.

[hec70143-bib-0035] Hoe, T. P. 2022. “Does Hospital Crowding Matter? Evidence From Trauma and Orthopedics in England.” American Economic Journal: Economic Policy 14, no. 2: 231–262. 10.1257/pol.20180672.

[hec70143-bib-0036] Jensen, V. M. , and M. Wüst . 2015. “Can Caesarean Section Improve Child and Maternal Health? The Case of Breech Babies.” Journal of Health Economics 39: 289–302. 10.1016/j.jhealeco.2014.07.004. 25179865

[hec70143-bib-0037] Jürges, H. 2017. “Financial Incentives, Timing of Births, and Infant Health: A Closer Look into the Delivery Room.” European Journal of Health Economics 18, no. 2: 195–208. 10.1007/s10198-016-0766-5. 26868529

[hec70143-bib-0038] Keulen, J. , P. Nieuwkerk , J. Kortekaas , et al. 2021. “What Women Want and Why. Women’s Preferences for Induction of Labour or Expectant Management in Late‐Term Pregnancy.” Women and Birth 34, no. 3: 250–256. 10.1016/j.wombi.2020.03.010. 32444268

[hec70143-bib-0039] Kreiner, C. T. , and H. H. Sievertsen . 2020. “Neonatal Health of Parents and Cognitive Development of Children.” Journal of Health Economics 69: 102247. 10.1016/j.jhealeco.2019.102247. 31837486

[hec70143-bib-0040] Lou, S. , K. Carstensen , L. Hvidman , et al. 2021. “‘I Guess Baby Was Just Too Comfy in There.’: A Qualitative Study of Women’s Experiences of Elective Late‐Term Induction of Labour.” Women and Birth 34, no. 3: 242–249. 10.1016/j.wombi.2020.03.012. 32404274

[hec70143-bib-0041] Lou, S. , L. Hvidman , N. Uldbjerg , et al. 2019. “(09). Women’s Experiences of Postterm Induction of Labor: A Systematic Review of Qualitative Studies.” Birth 46. 10.1111/birt.12412. 30561053

[hec70143-bib-0042] Maibom, J. , H. H. Sievertsen , M. Simonsen , and M. Wüst . 2021. “Maternity Ward Crowding, Procedure Use, and Child Health.” Journal of Health Economics 75: 102399. 10.1016/j.jhealeco.2020.102399. 33340811

[hec70143-bib-0043] Maimburg, R. D. 2020. “Induction of Labour in Earlier Gestational Weeks‐Reflections on Evidence and Clinical Practice.” Sexual & Reproductive Healthcare 23: 100487. 10.1016/j.srhc.2020.100487. 31982365

[hec70143-bib-0044] Mühlrad, H. 2022. “Cesarean Sections for High‐Risk Births: Health, Fertility, and Labor Market Outcomes.” Scandinavian Journal of Economics 124, no. 4: 1056–1086. 10.1111/sjoe.12474.

[hec70143-bib-0045] Neugart, M. , and H. Ohlsson . 2013. “Economic Incentives and the Timing of Births: Evidence From the German Parental Benefit Reform of 2007.” Journal of Population Economics 26, no. 1: 87–108. 10.1007/s00148-012-0420-1.

[hec70143-bib-0046] Place, K. , L. Rahkonen , A. Tekay , et al. 2023. “Labor Induction at 41+ 0 Gestational Weeks or Expectant Management for the Nulliparous Woman: The Finnish Randomized Controlled Multicenter Trial.” Acta Obstetricia et Gynecologica Scandinavica 103, no. 3: 505–511. 10.1111/aogs.14755. PMC10867371 38112629

[hec70143-bib-0047] Rydahl, E. , E. Declercq , M. Juhl , and R. D. Maimburg . 2019. “Routine Induction in Late‐Term Pregnancies: Follow‐Up of a Danish Induction of Labour Paradigm.” BMJ Open 9, no. 12: e032815. 10.1136/bmjopen-2019-032815. PMC6937045 31848171

[hec70143-bib-0048] Rydahl, E. , L. Eriksen , and M. Juhl . 2019. “Effects of Induction of Labor Prior to Post‐Term in Low‐Risk Pregnancies: A Systematic Review.” JBI Database of Systematic Reviews and Implementation Reports 17, no. 2: 170–208. 10.11124/jbisrir-2017-003587. PMC6382053 30299344

[hec70143-bib-0049] Schulkind, L. , and T. M. Shapiro . 2014. “What a Difference a Day Makes: Quantifying the Effects of Birth Timing Manipulation on Infant Health.” Journal of Health Economics 33: 139–158. 10.1016/j.jhealeco.2013.11.003. 24334004

[hec70143-bib-0050] Sievertsen, H. H. , and M. Wüst . 2017. “Discharge on the Day of Birth, Parental Response and Health and Schooling Outcomes.” Journal of Health Economics 55: 121–138. 10.1016/j.jhealeco.2017.06.012. 28743535

[hec70143-bib-0051] Simpson, M. , and C. Catling . 2016. “Understanding Psychological Traumatic Birth Experiences: A Literature Review.” Women and Birth 29, no. 3: 203–207. 10.1016/j.wombi.2015.10.009. 26563636

[hec70143-bib-0052] Smith‐Nielsen, J. , S. Matthey , T. Lange , and M. S. Væver . 2018. “Validation of the Edinburgh Postnatal Depression Scale Against Both dsm‐5 and icd‐10 Diagnostic Criteria for Depression.” BMC Psychiatry 18, no. 1: 1–12. 10.1186/s12888-018-1965-7. PMC6302501 30572867

[hec70143-bib-0053] Spillane, E. 2020. “Induction of Labour the Debate.” British Journal of Midwifery 28, no. 3: 142–143. 10.12968/bjom.2020.28.3.142.

[hec70143-bib-0054] Stotland, N. , A. Caughey , E. Breed , and G. Escobar . 2004. “Risk Factors and Obstetric Complications Associated With Macrosomia.” International Journal of Gynecology & Obstetrics 87, no. 3: 220–226. 10.1016/j.ijgo.2004.08.010. 15548393

[hec70143-bib-0055] Thomson, G. M. , and S. Downe . 2010. “Changing the Future to Change the Past: Women’s Experiences of a Positive Birth Following a Traumatic Birth Experience.” Journal of Reproductive and Infant Psychology 28, no. 1: 102–112. 10.1080/02646830903295000.

[hec70143-bib-0056] Tonei, V. 2019. “Mother’s Mental Health After Childbirth: Does the Delivery Method Matter?” Journal of Health Economics 63: 182–196. 10.1016/j.jhealeco.2018.11.006. 30594609

[hec70143-bib-0057] von Hinke, S. , N. Rice , and E. Tominey . 2022. “Mental Health Around Pregnancy and Child Development From Early Childhood to Adolescence.” Labour Economics 78: 102245. 10.1016/j.labeco.2022.102245.

[hec70143-bib-0058] Waldenström, U. , I. Hildingsson , C. Rubertsson , and I. Rådestad . 2004. “A Negative Birth Experience: Prevalence and Risk Factors in a National Sample.” Birth 31, no. 1: 17–27. 10.1111/j.0730-7659.2004.0270.x. 15015989

[hec70143-bib-0059] Wennerholm, U.‐B. , S. Saltvedt , A. Wessberg , et al. 2019. “Induction of Labour at 41 Weeks Versus Expectant Management and Induction of Labour at 42 Weeks (SWEdish Post‐Term Induction Study, SWEPIS): Multicentre, Open Label, Randomised, Superiority Trial.” BMJ 367. 10.1136/bmj.l6131. PMC6939660 31748223

[hec70143-bib-0060] WHO . 2011. WHO Recommendations for Induction of Labour. World Health Organization. 23586118

[hec70143-bib-0061] Zizzo, A. R. , I. Kirkegaard , A. Pinborg , and N. Ulbjerg . 2017. “Decline in Stillbirths and Perinatal Mortality After Implementation of a More Aggressive Induction Policy in Post‐Date Pregnancies: A Nationwide Register Study.” Acta Obstetricia et Gynecologica Scandinavica 96, no. 7: 862–867. 10.1111/aogs.13113. 28186614

