# Supplementary material for: Health and Economic Impacts of an Early Labor Induction Policy for High‐BMI Mothers
Source: Health Econ. 2026 Aug 5;35(12):1835–54. doi: 10.1002/hec.70143 (PMC13724286; doi:10.1002/hec.70143)
Supplement: Supplementary file 1 [file HEC-35-1835-s001.pdf]

Appendix - Health and Economic Impacts of an Early Labor Induction Policy for High-BMI Mothers

Version: August 3, 2026.

A Literature

A.1 Existing literature on early routine induction

When possible, we compare our results to studies with a credible causal design - especially studies reporting ITT estimates which are more directly comparable to the parameter we can causally estimate. While there is a lack of causal studies on whether having a high pre-pregnancy BMI justifies even earlier routine induction (Coates et al., 2020), we can compare our results to studies evaluating early induction for a general population of low-risk pregnancies. It is well-known that the risk of stillbirth increases with gestational length, especially after the EDD[1]. Generally, the discussion of early induction revolves around whether the benefits of induction earlier than 14 days after the EDD outweighs the costs (see Gregersen (2024) for details).

For the general population of low-risk pregnancies, there exist four RCTs evaluating routine induction at 7-9 days after the EDD instead of at 14 days after (Wennerholm et al., 2019; Keulen et al., 2019; Gelisen et al., 2005), and one comparing routine induction at 7 days after the EDD instead of at 12-15 days after (Place et al., 2024). Except for Place et al. (2024), all report ITT estimates.

Notably, a large randomized control trial in Sweden (SWEPSIS) was shut down early in 2020 because of increased mortality rates in the control group (no early induction) leading to much fewer participants than planned (Wennerholm et al., 2019). Yet, their contribution to the body of evidence makes it unlikely that setting up new RCTs would be ethically justifiable (Kenyon et al., 2019). With six deaths in the control group and no deaths in the early induction group, Wennerholm et al. (2019) cautiously report that early induction may

[1] See e.g. Muglu et al. (2019) for a recent review.

significantly reduce the risk of stillbirth. With the exception of SWEPSIS, none of the other RCTs find any statistically significant reduction of mortality risks, but the numbers always point in the direction of a reduction.

Analyzing combined individual data from Wennerholm et al. (2019) and Keulen et al. (2019), Alkmark et al. (2020) report a total reduction in mortality risks, an overall decreased risk of severe adverse perinatal outcomes, admission to the NICU, meconium-stained amniotic fluid and hypertension, but no impact on the rate of C-section. Birth weight and the risk of macrosomia were reduced too, and the use of pain relief during labor increased. The beneficial effects are driven by first-time mothers. Doing a standard meta-analysis adding the study by Gelisen et al. (2005), Alkmark et al. (2020) further confirm a reduction in their measure of severe adverse perinatal outcomes and no impact on the risk of C-sections, but do not report results for remaining outcomes. Because of early termination of the SWEPSIS study, Alkmark et al. (2020) may also suffer from the risk of overestimating treatment effects (Bassler et al., 2010).

The RCT by Gelisen et al. (2005) includes only 600 individuals, but find a reduced risk of macrosomia, shoulder dystocia, meconium-stained amniotic fluid and meconium aspiration syndrome in the early induction group. Remaining outcomes were similar across treatment and control group. The more recently published RCT by Place et al. (2024) was already running at the time SWEPSIS was terminated. They analyze results for a little less than 400 participants, and find a lower risk of instrumental delivery, having a large bleeding, and a birth weight$\geq$ 4,000 grams. They report no deaths and thus do not analyze mortality.

The slightly older review by Rydahl et al. (2019), includes one of the above RCTs (Gelisen et al., 2005) together with a RCT from Norway comparing induction at 9 days after the EDD with induction at 20 days after the EDD (Heimstad et al., 2007). Additionally, they consider two empirical evaluations of different procedures (either inductions at 8 days after the EDD or alternatively 14 to 15 days after the EDD) (Burgos et al., 2012; Daskalakis et al., 2014) as well as three pure correlational studies. The incidence of mortality is very rare, so even in their meta-study they lack statistical power to analyze it but the numbers point towards a reduction. They overall find a bit more mixed evidence on morbidity.[2] Yet, the empirical evaluations face different problems regarding causal interpretation. Burgos et al. (2012) use a before and after design and are thus not able to separate cohort effects from the procedure of early induction. Daskalakis et al. (2014) evaluate two different procedures applied at

[2]Focusing on the two RCTs and two empirical evaluations, they find that early induction affected morbidity in the following ways: increased risk of the child's PH value<7, C-section because of a failure to progress (but not overall C-Section risk), and precipitate labor, but reduced risk of meconium-stained amniotic fluid and oligohydramnious). (Some of) the included three cohort studies additionally showed increased risk of uterus rupture, overall risk of C-section + C-section after previous vaginal birth, instrumental delivery, labor dystocia, and chorioamnionitis.

the same hospital depending on physicians' preference. Thus, causal interpretation requires random allocation between patients and physicians which is likely not met in an empirical setting. The RCT by Heimstad et al. (2007) provides ITT estimates, and find no severe differences between control and treatment group, but the early induction group is more likely to experience precipitate labor and less likely to have a baby with birth weight<4,000 grams or oligohydramnios.

Several studies evaluate the introduction of new obstetric guidelines in Denmark in 2011. Specifically, they evaluate introducing surveillance at 7 days after the EDD together with routine induction at 10-13 days after the EDD instead of 14 days after for women age<40 and BMI<35 (Hedegaard et al., 2014, 2015; Wolff et al., 2016; Zizzo et al., 2017; Rydahl et al., 2019; Gregersen, 2024). Here we limit attention to the studies which address time trends in some way. When accounting for time trends, none of these studies find any evidence of a statistically significant reduction in mortality due to earlier routine induction.[3] Notice, that these studies evaluate a different treatment and population than what we do.

Wolff et al. (2016) find an increased risk of being admitted to the NICU (borderline significant) and a decreased risk of having a large baby, but no impact on remaining outcomes. Using an interrupted time-series analysis, Rydahl et al. (2019) find a large increase in risk of uterus rupture. At the time of implementation, they also find a smaller increase in epidural use as well as a smaller decrease in the risk of augmentation of labor, but both levels off over time.

Using a flexible difference-in-difference strategy, Gregersen (2024) finds no evidence on any (severe) morbidity effects despite a significant increase in the induction rate of more than 32% for first-time mothers with BMI<35 and age<40. Effectively, she is estimating ITT effects. She is unable to analyze the risk of uterus ruptures because of too few incidences in her sample, suggesting that Rydahl et al. (2019)'s result on uterus rupture may be driven by mothers with a previous C-section. While the main results show a reduced risk of having a large baby and increased use of epidural anesthesia, neither result is robust.

In contrast to most medical papers, Gregersen (2024) additionally follows mothers and children up to three years after birth and find no effect on the child's health care usage, mother's fertility or a proxy for mother's mental health during the first year after birth. A few medical papers show that either induced labor or induced birth are associated with adverse child health in the medium to longer-term (Dahlen et al., 2021; Peters et al., 2018) or find that the risk of being diagnosed with cerebral palsy within 3 years after birth is reduced in years with more liberal labor induction policies (Hedegaard et al., 2015), but they do not use a causal identification strategy.

[3]We refer to the Appendix in Gregersen (2024) for a detailed review.

A part of the discussion regarding earlier routine induction relates to mothers' birth experience which is harder to evaluate. In a RCT, Heimstad et al. (2007) provide ITT estimates and report evidence favoring routine induction at 7 days after the EDD instead of continued monitoring and awaiting spontaneous birth. Not only did the majority of mothers prefer to be induced at 7 days after the EDD, but the majority of mothers who were induced early had a positive experience and would even prefer the same management in a future pregnancy. Mothers exposed to early induction had shorter duration of labor but experienced more intense and frequent contractions too.

However, Keulen et al. (2021) report heterogeneous preferences for induction among women at one week after the EDD. Several studies further find that induction of labor is associated with less satisfaction, a less positive birthing experience, and a higher concern for the newborn's safety (Shetty et al., 2005; Waldenström et al., 2004; Hildingsson et al., 2011). In an online survey, the majority of women with induced labor would prefer not to be induced in the future (Schwarz et al., 2016). Moreover, they felt they lacked both information and support in their decision-making. These points are supported by a review of qualitative studies which points to a need to provide better information on the induction process as well as to help women adjust their birth expectations when inducing labor (Lou et al., 2019). In the Danish context, a small qualitative study showed that all women routinely induced had preferred to give spontaneous labor (Lou et al., 2021). While the majority had a positive birth experience, some would have liked more time and information to make the decision.

B Additional institutional context: High-BMI pregnancies in Denmark

The aim of this section is to describe general guidelines and background information relevant for high-BMI pregnancies in Denmark. Overweight and obese pregnancies are associated with increased maternal and child health risks. While the cause is unknown, metabolic issues and insulin resistance are possible explanations (SSI, 2013). Specifically, DSOG (2017) lists increased risks of pregnancy complications such as hypertension, preeclampsia and gestational diabetes (GDM) along with unfavorable outcomes such as miscarriage, very preterm birth ($\leq$28 weeks), and even perinatal death. Moreover, children born from overweight mothers face increased risks of congenital malformations and a higher likelihood of ending up being overweight in the long term. Not only do birth interventions such as labor induction, instrumental delivery, and C-section happen more frequently, but the risks associated with C-section such as infections, a large bleeding and anesthesia complications are higher too.

The risk of having a large baby (macrosomia) increases with maternal BMI, which carries both overlapping and separate risks [4]. Detecting large babies is linked with uncertainty - especially for overweight women (DSOG, 2007b, 2018). Guidelines effective during our sample periods favor a planned C-section when the estimated birthweight $\geq$ 4.8 kg (4.5 kg in case of any type of diabetes (DM)).

Throughout the study period, the risks associated with being overweight and obese are recognized and addressed in general guidelines (DSOG, 2003; SSI, 2009, 2013). The potential benefits of giving birth at a larger hospital with access to specialized care are already highlighted by the first DSOG task force focusing on overweight in 2003 (DSOG, 2003) and advised by some gynecologists in 2012 (Vinter et al., 2012) and certain hospitals in 2011 (Jungensen, 2011). However, this did not become a part of the national guidelines until 2017 (DSOG, 2017).

During our sample period, overweight pregnancies are offered counseling regarding reducing weight gain during pregnancy as well as encouraged to engage in at least 30 minutes of daily physical activity (SSI, 2009). While weight loss during pregnancy is not recommended, ensuring a healthy lifestyle during pregnancy and losing weight prior to becoming pregnant were advised. Pregnancies with a BMI$\geq$35 might receive additional checks and surveillance including additional ultrasound scans to help estimate the size of the fetus as well as the fetus's position in the womb[5] (SSI, 2009). Yet, the determination of these factors becomes more challenging and less precise for overweight and obese women (SSI, 2013). Moreover, pregnancies with a BMI$\geq$35 may be referred to a consultation with an obstetrician during pregnancy. This consultation may involve discussing potential birth complications and the possibility of using epidural anesthesia prophylactically (SSI, 2009). Several guidelines related to the risks and consequences of being overweight or dealing with suspected macrosomia emphasize that an overall evaluation of individual risks and preferences should be considered when determining the preferred birth mode and best plan of action for each pregnancy (DSOG, 2018, 2017). In particular, prior pregnancy and birth history should be taken into account when deciding the best course of action.

Throughout the study period, a BMI $\geq$ 27 meant that the woman would undergo at least one glucose test to detect possible cases of GDM. If diagnosed with GDM, special guidelines for surveillance as well as recommended time and mode of birth will be implemented (DSOG, 2007a). Similarly, special guidelines are effective for pregnancies with pre-existing

[4] For the child, DSOG (2017) list risks such as shoulder dystocia, plexus brachialis lesions, asphyxia, and stillbirth. Maternal risk includes a large bleeding (>500 ml), a severe laceration (degree 3 and 4) and the risk of an emergency C-section. Average hospitalization (for both mother and child) are longer too.

[5] Procedures may vary across hospitals. In 2012, using an ultrasound scan at weeks 35-38 to obtain a weight estimate of the child for overweight pregnancies was standard procedure at some hospitals (Vinter et al., 2012).

DM (DSOG, 2010). In general, guidelines recommend induction no later than at the EDD/7 days after the EDD when insulin treatment is required/not required. Yet, if the weight estimate is above 4 kg/4.5 kg even early induction/a planned C-section is recommended.

A final health policy relevant to our study is restricted access to fertility treatment based on BMI. Most fertility clinics restrict treatment to women with BMI below a certain threshold (sometimes at 35, but it varies between 29 and 40 across clinics and age-groups).

C Additional Data Information

C.1 Controls: pre-birth parental background variables and other indications for induction before the EDD

We use the Land Patient Register (LPR) to recover measures of certain pregnancy complications including prior C-section (ICD-10:O82), insufficient fetal growth (0365), prior large baby (birth weight$\geq$4.5 kg), having a multiple birth, first time-mother, or preeclampsia (O11.9). To avoid bad control issues, we only consider conditions registered prior to the EDD.

Aside from the LPR, the set of background variables we use to lead balances of observable tests and adjust the RD estimates are also extracted from administrative register data from Statistics Denmark. This data includes parental background characteristics such as a dummy if either parent graduated from college, monthly joint parental income in 2024 US dollars, maternal age, a dummy which equals one if either parent was born in Denmark, a dummy for whether parents cohabit, as well dummies for missing information on any of the control variables.

From the MFR (birth register), we also extract measures of the hospital, of the day of the week of birth, and of the month of birth to build fixed-effects. Hospital fixed-effects are especially important to account for differences in practices across hospitals.

C.2 Descriptives in our main sample

To understand how risk profiles vary across the BMI cutoff, Table C.1 shows descriptive statistics for our main sample of pregnancies going at least 7 days past the EDD, both in total (column 1), and split by mothers with a BMI < 35 (column 2), and those with BMI ≥ 35 (column 3).

Mothers with a BMI ≥ 35 exhibit an increased likelihood of experiencing certain complications and adverse birth outcomes such as macrosomia (birth weight$\geq$4.5 kg), admission to the NICU, and longer hospital stays. They are also more likely to have a high risk of postpartum depression (EPDS score > 11) and to belong to a lower socioeconomic status, as evidenced by lower parental income, education level, and employment status. However, certain birth outcomes are also better for this group of mothers as the risk of hemorrhage, lacerations, and shoulder dystocia are all lower.

These descriptive differences are hard to interpret. At least three different forces are at play. First, ceteris paribus, higher maternal BMI is widely acknowledged as a risk factor associated with worse outcomes (Coates et al., 2020) (see Appendix B for details). Secondly, since we focus on births taking place at least 7 days after the EDD, our population is positively

selected. Indeed, severe complications or risk-factors indicate induction or a planned C-section prior to the EDD. Thirdly, mothers above the BMI cutoff are 20 p.p. more likely to be ever induced than those below. This intervention can improve or deteriorate neonatal and maternal health or may be a result of unobserved underlying health differences. Hence, absent an identification strategy, it is impossible to explain the observable differences in neonatal health.

Tab. C.1 Variable means, births taking place at least 7 days after the EDD (2011-2017)

	All births	BMI<35	BMI>=35	t-test
Any parent college	0.559	0.569	0.405	0.000
Parental income, monthly (dkk)	6446.790	6501.080	5522.698	0.000
Any parent Danish	0.868	0.867	0.880	0.005
Parents are cohabiting	0.827	0.828	0.803	0.000
First-time mother	0.510	0.512	0.480	0.000
Maternal age	30.554	30.590	29.940	0.000
Preeclampsia	0.008	0.007	0.011	0.006
Insufficient growth	0.017	0.017	0.014	0.023
Prior C-section	0.067	0.066	0.088	0.000
Prior birth weight $\geq$ 4.5kg	0.013	0.012	0.022	0.000
Induced labor at any gestational age	0.393	0.380	0.610	0.000
Induced 7 and 9 days after EDD	0.067	0.053	0.307	0.000
Epidural	0.442	0.437	0.525	0.000
Elective C-Section	0.007	0.007	0.010	0.013
Emergency C-section	0.049	0.049	0.058	0.005
Vacuum extraction	0.099	0.101	0.072	0.000
Gestational age (days)	290.211	290.254	289.467	0.000
Birth weight	3770.038	3767.181	3818.702	0.000
Apgar <7	0.007	0.007	<0.01	0.038
Birthweight>4.5 kg	0.057	0.056	0.073	0.000
Share sent in NICU	0.056	0.055	0.068	0.000
Hospital Nights	2.163	2.150	2.398	0.000
Shoulder dystocia	0.007	0.007	<0.01	0.744
Lacerations	0.330	0.335	0.253	0.000
Lacerations 3rd degree higher	0.034	0.034	0.026	0.000
Hemorrhage	0.517	0.523	0.417	0.000
Complication index	0.007	0.007	<0.01	0.233
EPDS>11	0.056	0.056	0.060	0.494
EPDS Score	4.838	4.821	5.105	0.003
Nurses visits	4.585	4.589	4.510	0.010
GP Visit (1st year)	16.943	16.873	18.123	0.000
Mother employed 2 years post birth	0.870	0.874	0.801	0.000
Prob.another child two years post birth	0.025	0.025	0.023	0.340
Observations	97604	92188	5416	

Notes: The following table displays the mean values of various variables for all births in Denmark that reached at least 7 days after the EDD. It also includes the mean values for mothers with a BMI < 35, those with a BMI ≥ 35, and the p-value from a two-tailed t-test comparing the two subgroups.

D Additional analyses

D.1 Treatment & First Stage (robustness)

Tab. D.1 First stage: ITT effect on early labor induction (7-9 days after the EDD) across samples

	(1)	(2)	(3)
Sharp	0.148***	0.147***	0.197***
	(0.025)	(0.032)	(0.046)
Covariates	Yes	Yes	Yes
(Effective) Observations	17317	10994	5233
Mean Control Group	0.080	0.082	0.085
Sample	All births	Nurses visits	EPDS Score

Standard errors in parentheses

* $p < 0.10$, ** $p < 0.05$, *** $p < 0.01$

Notes: Standard errors are robust and clustered at the hospital-year level. * $p < 0.10$, ** $p < 0.05$, *** $p < 0.01$.This table shows RD estimates from a sharp RDD with being induced 7-9 days after the EDD as the outcome, the mother's BMI as the running variable, and 35 as the cutoff. The bandwidth is manually set to 7 for reasons explained in subsection D.3. "Mean in the control group" refers to the average outcome for births on the left-side of the bandwidth (Maternal BMI 28-35). The set of controls is described in subsection C.1. In the sample restriction row, "All births" refer to all births in Denmark between 2011 and 2017 that occurred at 7 days after the EDD or later, whereas "Nurse visits" and "EPDS score" refer to the respective subsets of this sample for which nurse visits from the NHV and mental health data are available.

Tab. D.2 Ever induced

	Sharp	Sharp	Sharp	Fuzzy	Fuzzy	Fuzzy
Ever induced	0.083***	0.059*	0.041	0.558***	0.418***	0.218
	(0.025)	(0.031)	(0.042)	(0.124)	(0.158)	(0.164)
Obs	17317	10994	5233	17317	10994	5233
Mean Control Group	0.459	0.462	0.472	0.459	0.462	0.472
Sample	All births	Nurses visits	EPDS Score	All births	Nurses visits	EPDS Score

Notes: Standard errors are robust and clustered at the hospital-year level. * $p < 0.10$, ** $p < 0.05$, *** $p < 0.01$.This table shows RD estimates with labor induction at any time as the outcome, the mother's BMI as the running variable, and 35 as the cutoff. The bandwidth is manually set to 7 for reasons explained in subsection D.3. "Mean in the control group" refers to the average outcome for births on the left-side of the bandwidth (Maternal BMI 28-35). The set of controls is described in subsection C.1. In the sample restriction row, "All births" refer to all births in Denmark between 2011 and 2017 that occurred at 7 days after the EDD or later, whereas "Nurse visits" and "EPDS score" refer to the respective subsets of this sample for which nurse visits from the NHV and mental health data are available.

Tab. D.3 Gestational length (days past the EDD)

	Sharp	Sharp	Sharp	Fuzzy	Fuzzy	Fuzzy
Gest. Days	-0.678***	-0.654***	-1.096***	-4.304***	-4.287***	-5.287***
	(0.149)	(0.177)	(0.253)	(0.689)	(0.919)	(1.102)
Obs	17317	10994	5233	17317	10994	5233
Mean Control Group	10.363	10.361	10.333	10.363	10.361	10.333
Sample	All births	Nurses visits	EPDS Score	All births	Nurses visits	EPDS Score

Notes: Standard errors are robust and clustered at the hospital-year level. * $p < 0.10$, ** $p < 0.05$, *** $p < 0.01$. This table shows RD estimates with gestational length in days (past the EDD) as the outcome, the mother's BMI as the running variable, and 35 as the cutoff. The bandwidth is manually set to 7 for reasons explained in subsection D.3. "Mean in the control group" refers to the average outcome for births on the left-side of the bandwidth (Maternal BMI 28-35). The set of controls is described in subsection C.1. In the sample restriction row, "All births" refer to all births in Denmark between 2011 and 2017 that occurred at 7 days after the EDD or later, whereas "Nurse visits" and "EPDS score" refer to the respective subsets of this sample for which nurse visits from the NHV and mental health data are available.

Fig. D.1 Effects on early induction and gestational length across different bandwidth, polynomial order, and kernel choices

(a) Induced at 7-9 after the EDD

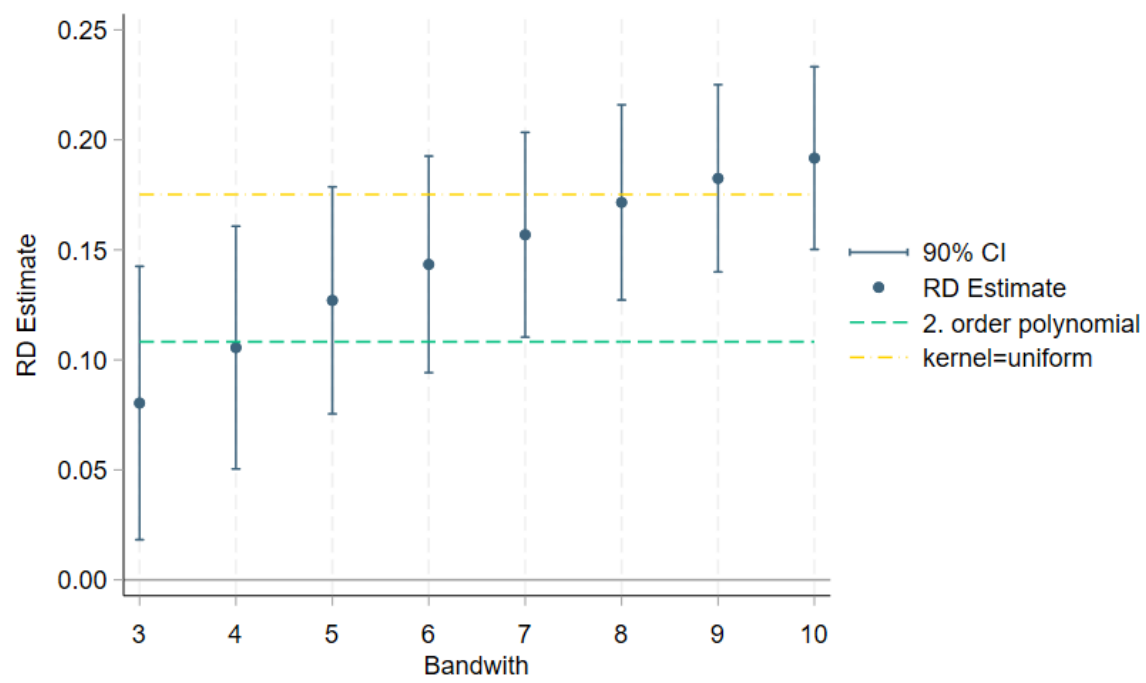

(b) Gestational length (days after the EDD)

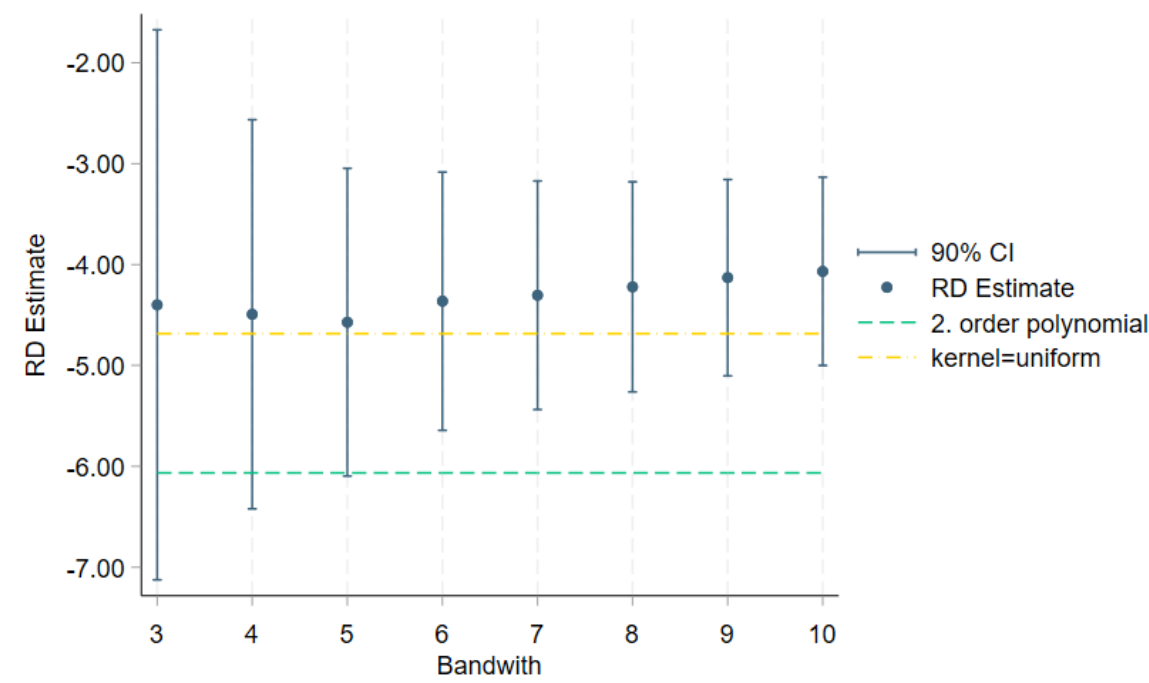

Notes: The two figures are coefficient plots showing estimates on early labor induction (left panel) and gestational length measured in days after the EDD (right panel). All estimates on the left panel are from the sharp regression discontinuity design (RDD) as specified in Equation 1 (as it is the first stage), and all estimates on the right panel are from a fuzzy RDD as specified in Equation 2. Point estimates with higher polynomial order and different kernel are computed with a bandwidth of seven, as in our main specification. Standard errors are robust and clustered at the hospital-year level. Sample is the main one (all births in Denmark between 2011 and 2017 that occurred at 7 days after the estimated due date (EDD) or later).

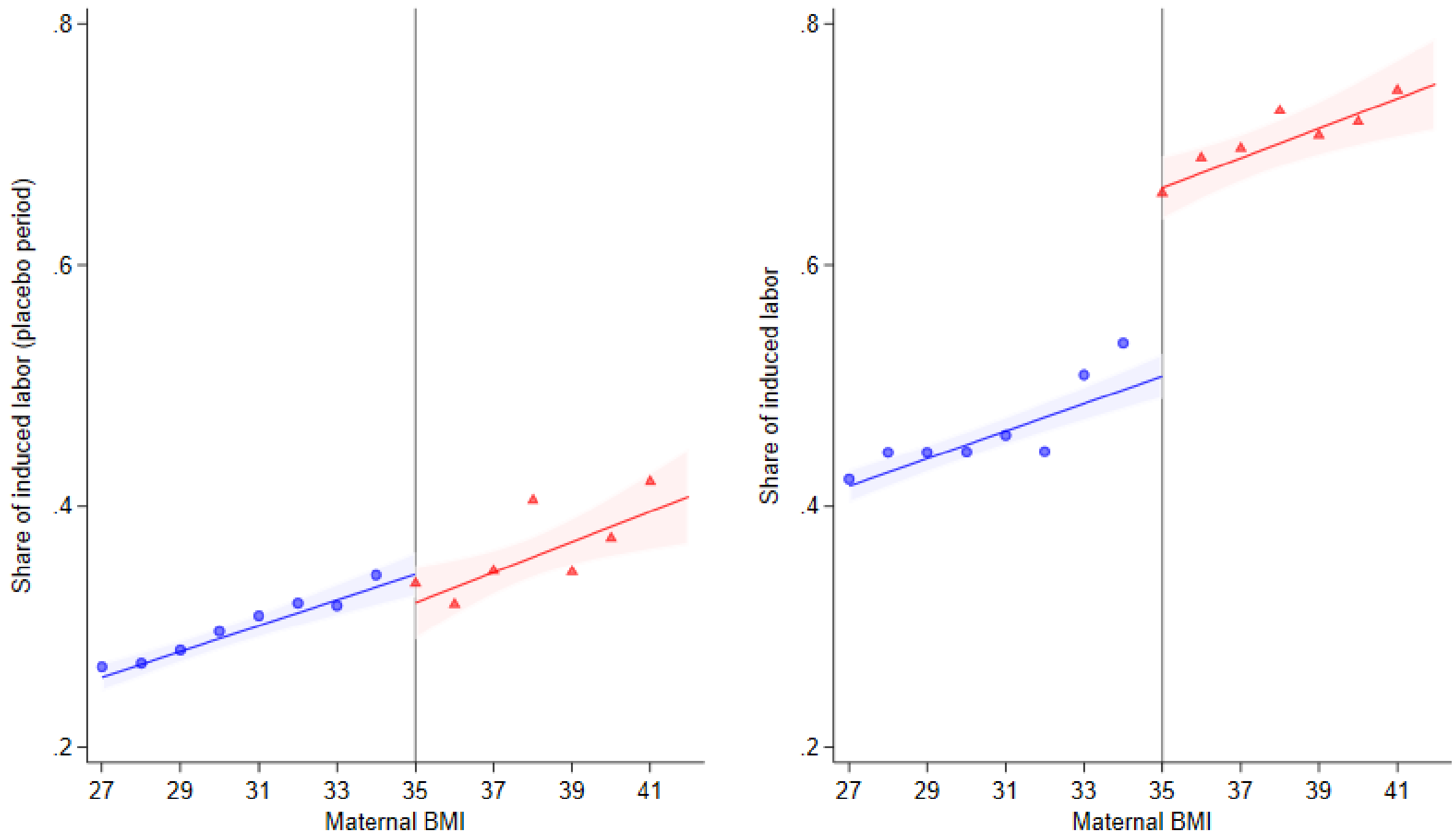

Fig. D.2 Ever being induced in placebo period (2004-2009) and main sample (2011-2017)

Notes: The bins are the average outcomes for each integer BMI unit. The lines are fitted lines from an OLS regression on all births where the mother gave birth at least 7 days after the estimated due date (EDD), have an age<40, and no registry of gestational diabetes. Confidence intervals are at the 90 percent level and built from heteroskedastic robust SEs clustered at the hospital-year level. The right panel sample is from the period after the early induction BMI-based guidelines were issued (2011-2017), while the left panel sample is from the placebo period (2004-2009).

D.2 Effect on Healthcare usage across samples

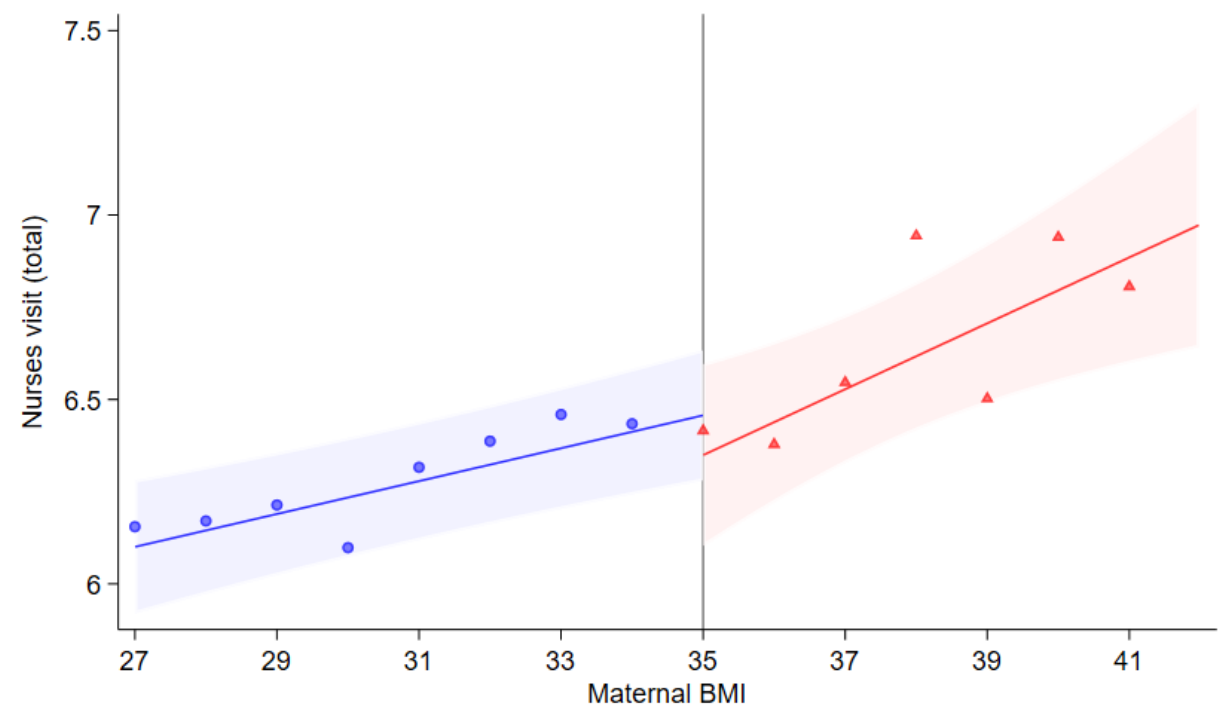

Fig. D.3 Discontinuity in the number of total nurse visits (first year after birth)

Notes: The bins are the average outcomes for each integer BMI unit. The lines are fitted lines from an OLS regression on all births between 2011 and 2017 where the mother gave birth at least 7 days after the estimated due date (EDD), have an age<40, and no registry of gestational diabetes as well as being a part of the Nurse visit sample with a total of n=11,025. Confidence intervals are at the 90 percent level and built from heteroskedastic robust SEs clustered at the hospital-year level. No placebo period is shown because the data from the NHV is not available before 2011.

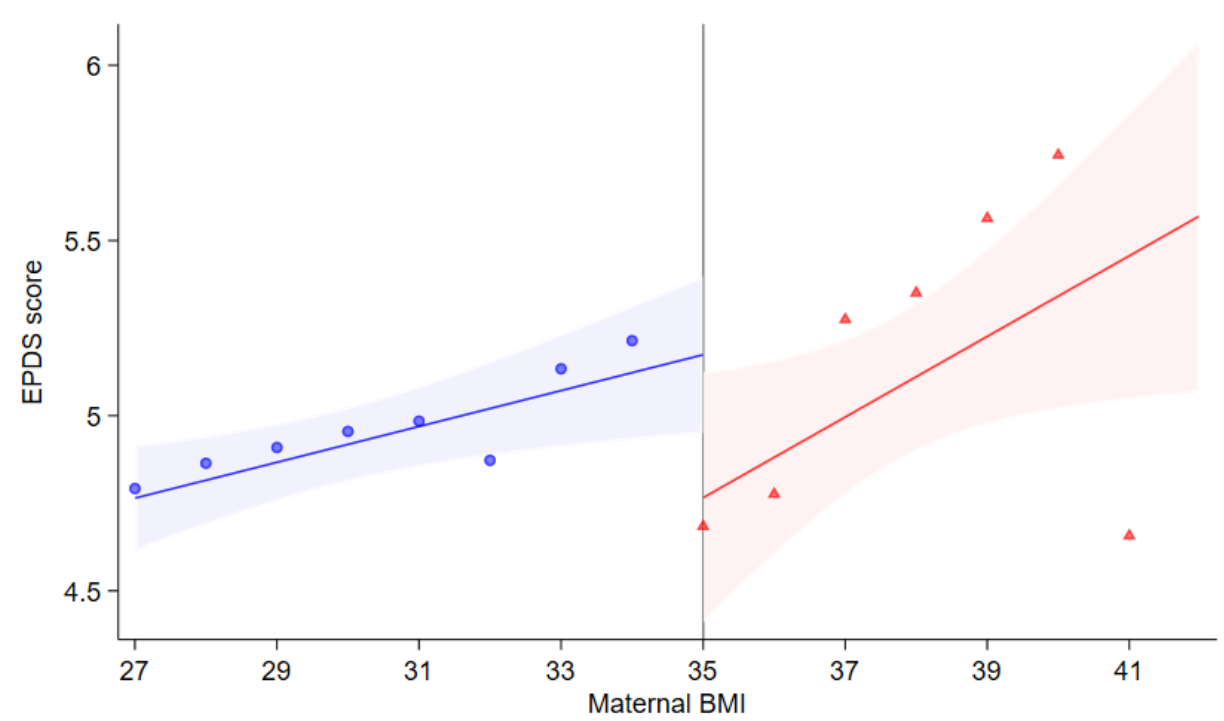

Fig. D.4 Discontinuity in maternal mental health (EPDS score)
Notes: The bins are the average outcomes for each integer BMI unit. The lines are fitted lines from an OLS regression on all births between 2011 and 2017 where the mother gave birth at least 7 days after the estimated due date (EDD), have an age<40, and no registry of gestational diabetes as well as information on EPDS score with a total of n=5233. Confidence intervals are at the 90 percent level and built from heteroskedastic robust SEs clustered at the hospital-year level. No placebo period is shown because the data from the NHV is not available before 2011.

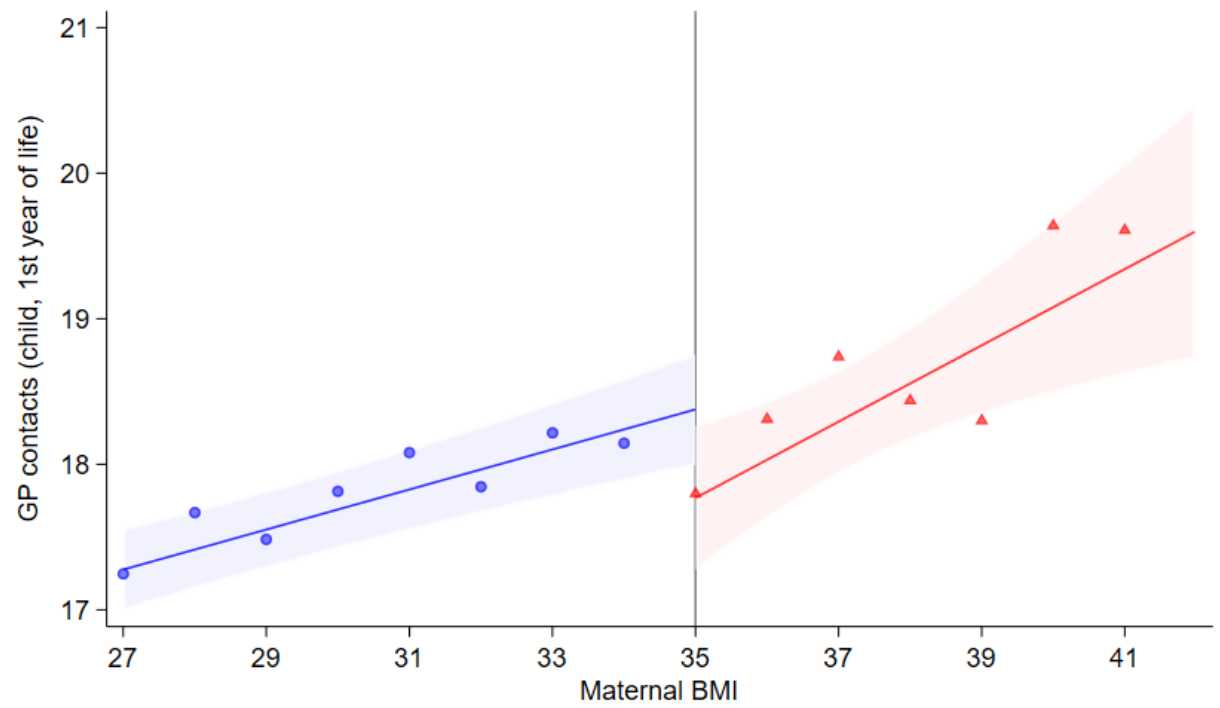

Fig. D.5 Discontinuity in the number of GP Visits (1st year of life)
Notes: The bins are the average outcomes for each integer BMI unit. The lines are fitted lines from an OLS regression on all births between 2011 and 2017 where the mother gave birth at least 7 days after the estimated due date (EDD), have an age<40, and no registry of gestational diabetes. Confidence intervals are at the 90 percent level and built from heteroskedastic robust SEs clustered at the hospital-year level.

Tab. D.4 Impact on healthcare usage (Nurse visits and EPDS samples)

	Fuzzy	Obs	Fuzzy	Obs
Nurses visits (total)	-2.351**	11025	-3.494***	5233
	(1.136)		(1.265)	
Univ. Nurses visits	-2.534***	11025	-2.128***	5233
	(0.756)		(0.559)	
Add. Nurses visits	-0.149	11025	-1.695	5233
	(0.973)		(1.262)	
Covariates	Yes		Yes	
Sample	Nurses Visit		EPDS Score	

Notes: Standard errors are robust and clustered at the hospital-year level. * $p < 0.10$, ** $p < 0.05$, *** $p < 0.01$. This table displays RDD estimates which are obtained using our favorite specification with polynomial order of one and an epanechnikov kernel. The bandwidth is manually set to 7 for reasons explained in subsection D.3. The set of controls is described in subsection C.1. In the sample restriction row, "All births" refer to all births in Denmark between 2011 and 2017 that occurred at 7 days after the EDD or later, whereas "Nurse visits" and "EPDS score" refer to the respective subsets of this sample for which nurse visits from the NHV and mental health data are available.

D.3 Power Analysis and picking the appropriate bandwidth

Since the RDD is a data-demanding design (Stommes et al., 2023; Goldberger, 2008) and the group of mothers with a pre-pregnancy BMI≥35 is rather small, we here address the power issue in detail. In Figure D.6 below, we show the share of all ongoing pregnancies with a BMI above the 35 threshold to highlight our power issue. Specifically, out of all pregnancies passing the EDD, only a relatively low share has a BMI of at least 35. Once again, remember that mothers with many types of comorbidity are induced or receive a planned CS prior to the EDD.

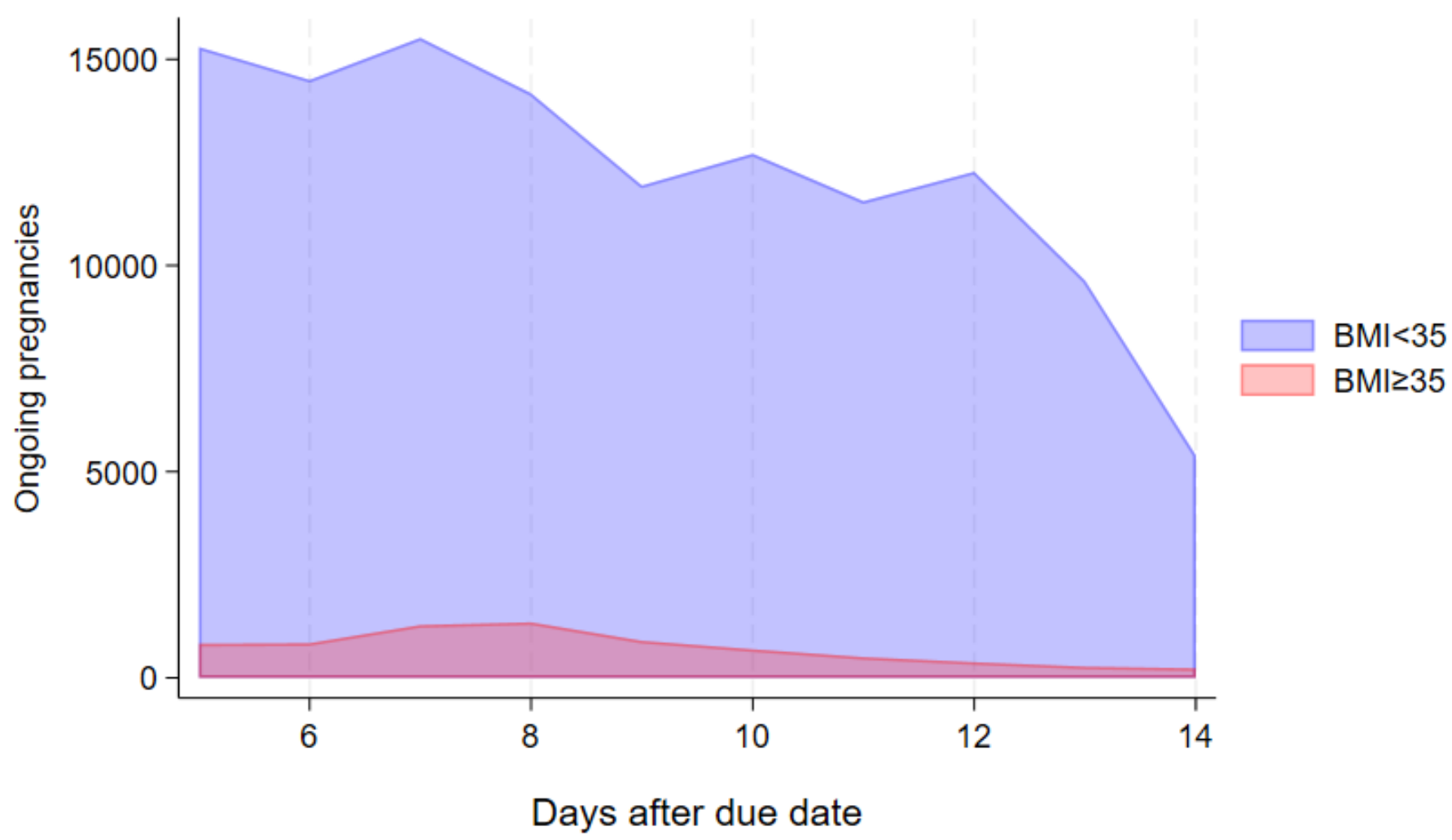

Fig. D.6 Total ongoing births by days after the estimated due date (EDD) and BMI category

Notes: The figure shows the total ongoing births by days after the estimated due date (EDD) and BMI category (BMI<35 or BMI $\geq$ 35). The sample includes all births in Denmark between 2011 and 2017 with a gestational length of more than four days past the EDD.

In Table D.5, we standardize our outcomes to compute the probability to detect a strong effect (Standardized Mean Difference (SMD) $\geq$ 0.8) when alpha equals 0.1 for all of the outcomes in our main analysis. The first column displays power results with Calonico et al. (2017)'s optimal data-driven bandwidth algorithm. It is between 3 or 6 depending on the outcomes. Strikingly, the probability to detect a strong effect is below the 80 percent cutoff for all of our outcomes but hospital nights. This is despite two generous hypotheses regarding the alpha and the size of the effect. Opting for an optimal data-driven bandwidth can only lead to underpowered analyses in our setting. Hence, in the second column of the power table, we enlarge the bandwidth and set it to seven for every outcome. This allows analyses for almost all of our outcomes to be reasonably powered. However, increasing the polynomial

order by one renders all tests underpowered again. These power results motivate two central choices in the rest of our analysis.

Tab. D.5 Power analysis: probability to detect an effect of 0.8 SD

	Data-driven bandwidth	Bandwidth=7 (p=1)	Bandwidth=7 (p=2)
Epidural	0.299	0.951	0.656
Apgar <7	0.460	0.989	0.737
Share sent in NICU	0.395	0.984	0.718
Birthweight $\geq$ 4.5 kg	0.411	0.977	0.663
Hospital Nights	0.826	0.998	0.850
Uterus Rupture	0.461	0.986	0.730
Birth Weight (grams)	0.672	0.998	0.815
Lacerations	0.508	0.992	0.759
Hemorrhage	0.556	0.977	0.698
Shoulder dystocia	0.730	1.000	0.971
Emergency C-section	0.637	0.992	0.732
Lacerations	0.508	0.992	0.759
Lacerations(3rd degree and higher)	0.484	0.999	0.831
Vacuum extraction	0.524	0.998	0.850
Complications index	0.541	0.999	0.874

Notes: The table shows the probability to detect an effect of 0.8 standard deviation for various outcomes and varying specification choices. Sample is the main one containing all births in Denmark between 2011 and 2017 that occurred at 7 days after the EDD or later.

First, in our central specifications, we set the bandwidth to seven rather than using a data-driven bandwidth. It is necessary for our analyses to be sufficiently powered. Naturally, since our manual bandwidth is larger than the optimal bandwidth, this choice increases the risk of bias. However, the various balancing tests (see Table 1) and placebo tests (see sections 5 and 6) we conducted speak against major bias and in favor of the continuity hypothesis. Moreover, when the outcomes are available before the guidelines, we also check there is no discontinuity before the introduction to the guidelines. Together, these robustness checks suggest the probability our results are confounded is minimal, despite us picking a bandwidth slightly higher than the optimal one. Moreover, using coefficient plots (Appendices D.5 and D.6), we make sure that the coefficients are stable across specifications when the bandwidth is increased or decreased.

Second, we set the polynomial order to one in our central specifications. This is again for power reasons. Again, to ensure more complex functional forms of the function linking the running variable to the outcomes are not mistaken for discontinuities, we also check that the coefficients are stable when increasing the polynomial order using coefficient plots. In those coefficient plots, however, we never set the polynomial order above 2, since Gelman and Imbens (2019) showed higher order polynomials implied noisy estimates and poor coverage of confidence intervals.

D.4 Bunching analysis

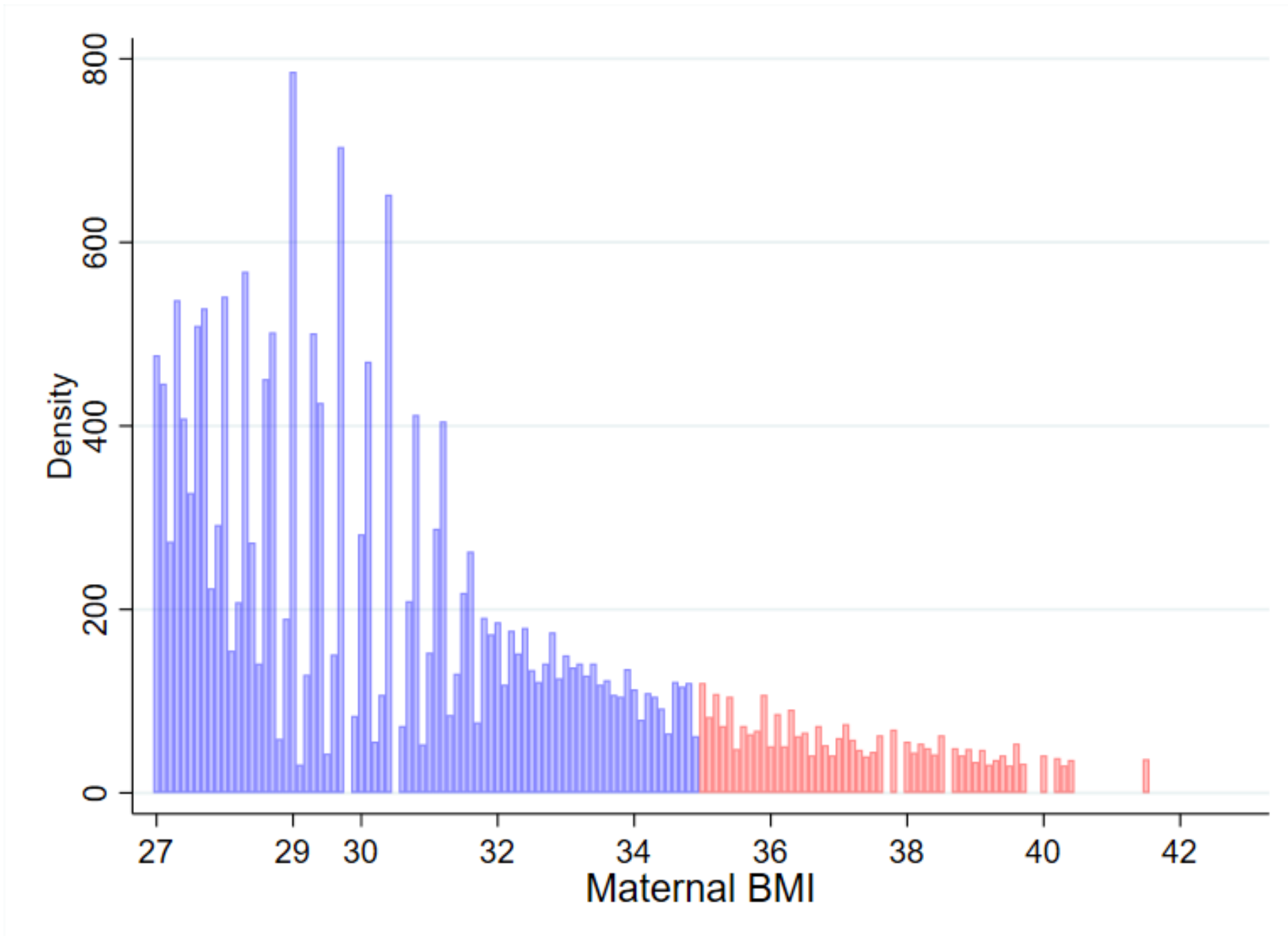

Fig. D.7 Density of births with a given maternal BMI
Notes: The Figure shows the density of births around the 35 maternal BMI threshold. Blue bars indicate that the maternal BMI is below the threshold, red bars that the maternal BMI is above. Sample is the main one containing all births in Denmark between 2011 and 2017 that occurred at 7 days after the EDD or later. While our maternal BMI data is available up to three digits, we collapsed data at the first digit to abide with our provider rules. All bins with less than 30 obs are deleted. The p-value of the McCrary density test is 0.4554.

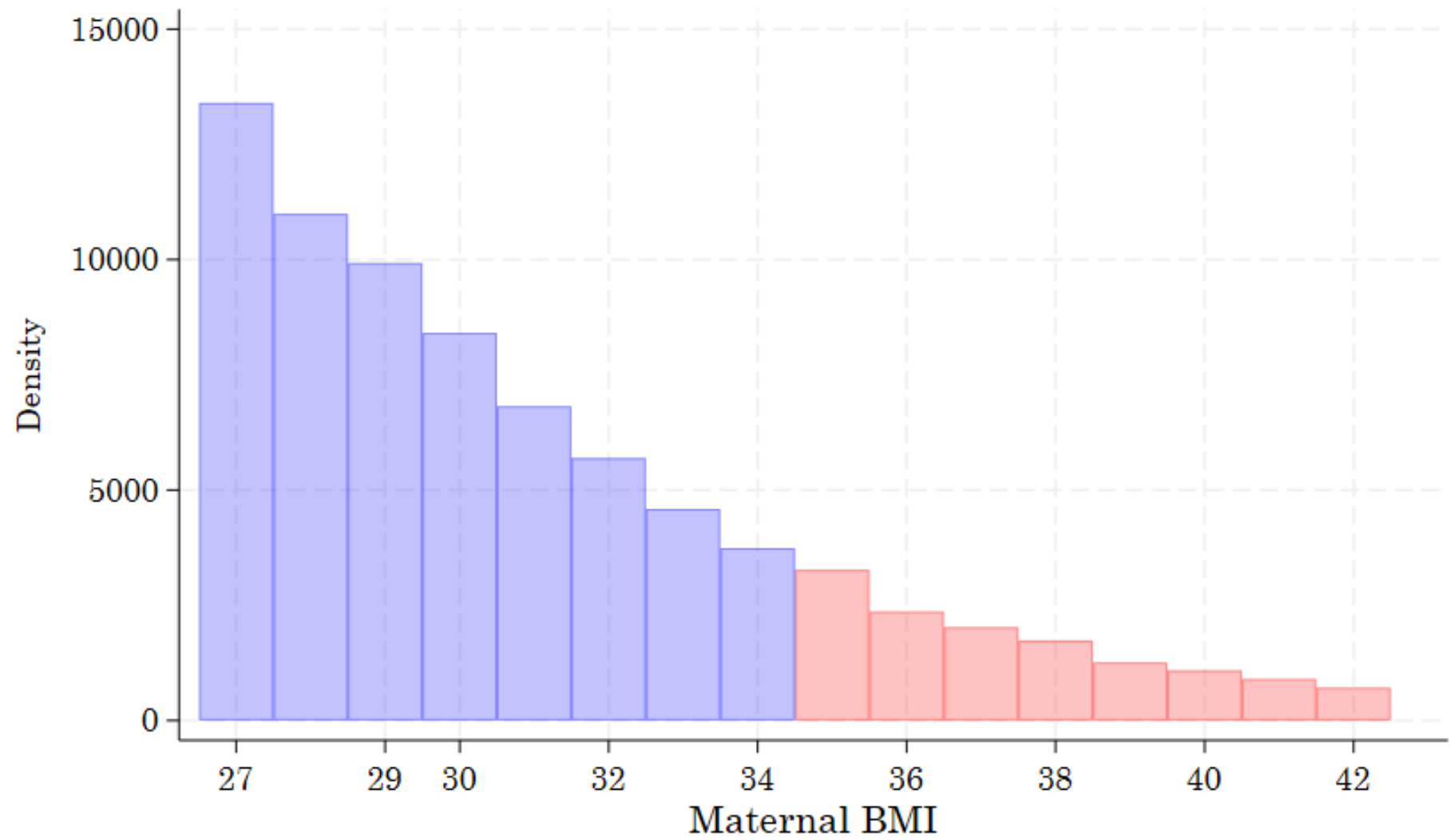

Fig. D.8 Density of births with a given maternal BMI (for births before seven days after the EDD)
Notes: The Figure shows the density of births around the 35 maternal BMI threshold in the alternative sample of mothers who give birth before 7 days after EDD, and are thus unaffected by the guidelines. Blue bars indicate that the maternal BMI is below the threshold, red bars that the maternal BMI is above. Sample is the main one containing all births in Denmark between 2011 and 2017 that occurred prior to 7 days after the EDD. All bins with less than 30 obs are deleted. The p-value of the McCrary density test is 0.1387.

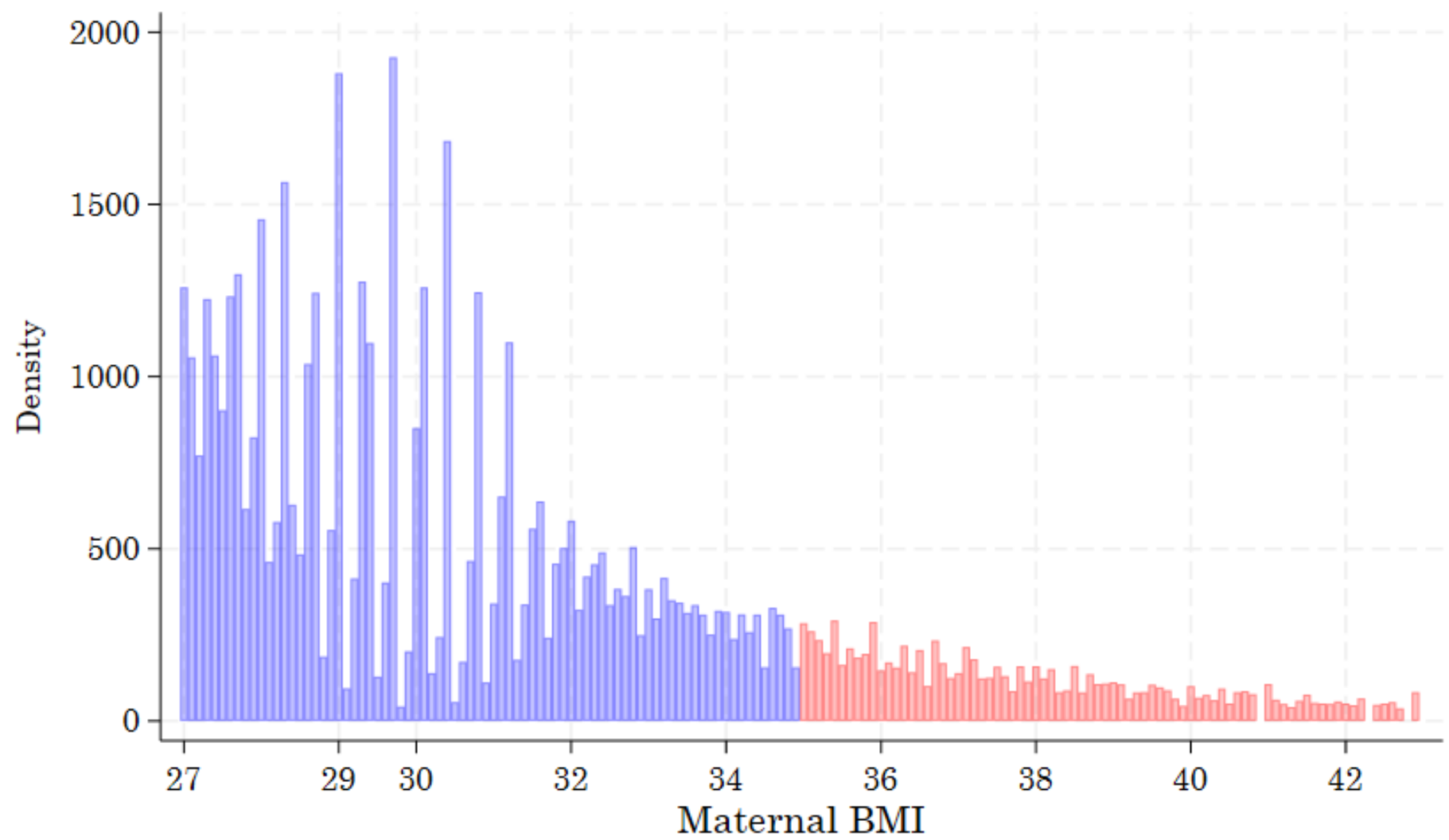

Fig. D.9 Density of births with a given maternal BMI (for births before seven days after the EDD)
Notes: The Figure shows the density of births around the 35 maternal BMI threshold in the alternative sample of mothers who give birth before 7 days after EDD, and are thus unaffected by the guidelines. Blue bars indicate that the maternal BMI is below the threshold, red bars that the maternal BMI is above. Sample is the main one containing all births in Denmark between 2011 and 2017 that occurred prior to 7 days after the EDD. While our maternal BMI data is available up to three digits, we collapsed data at the first digit to abide with our provider rules. All bins with less than 30 obs are deleted. The p-value of the McCrary density test is 0.1387.

D.5 Coefficient plots for immediate outcomes

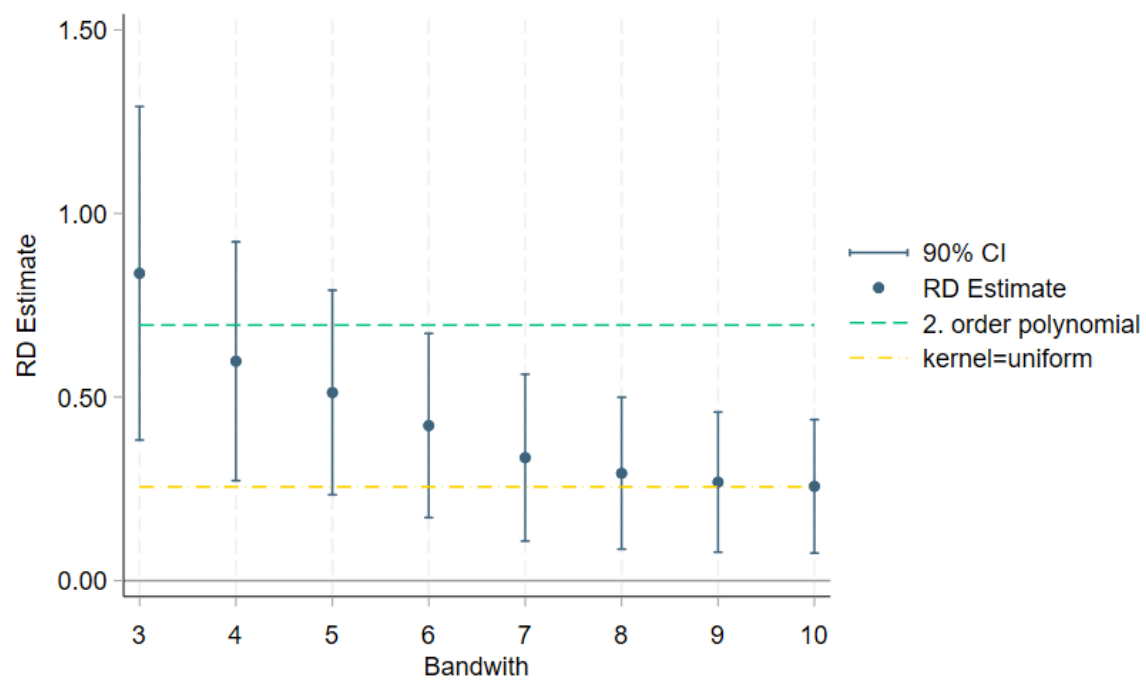

(a) Epidural

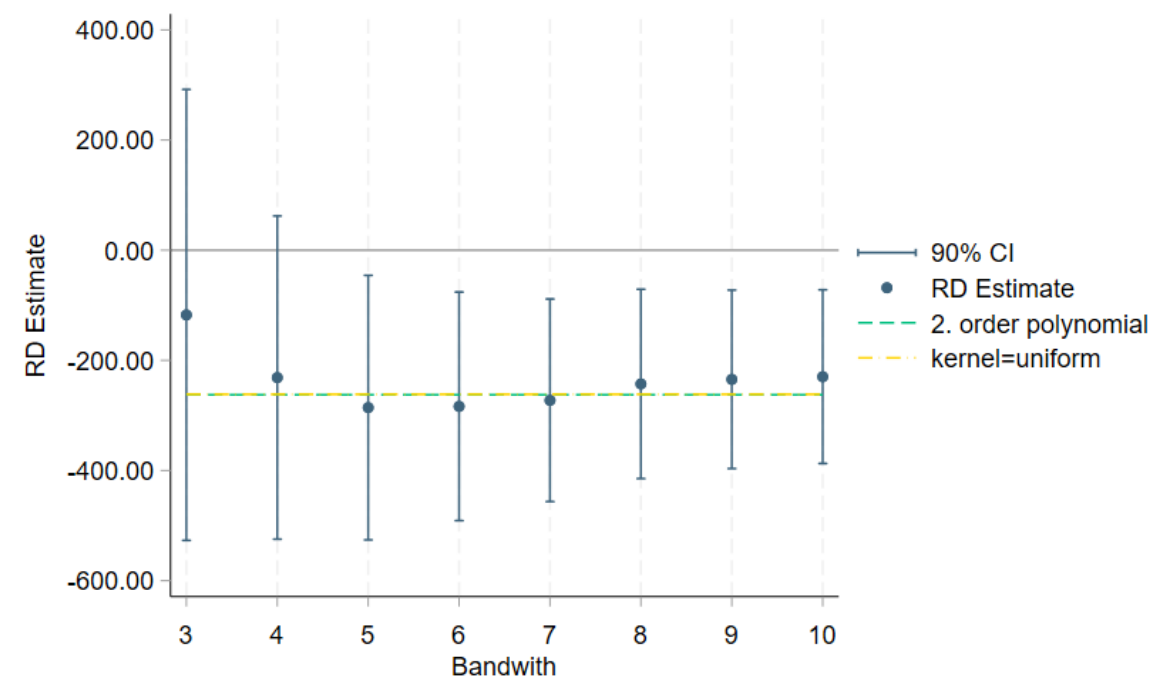

(b) Birth weight (grams)

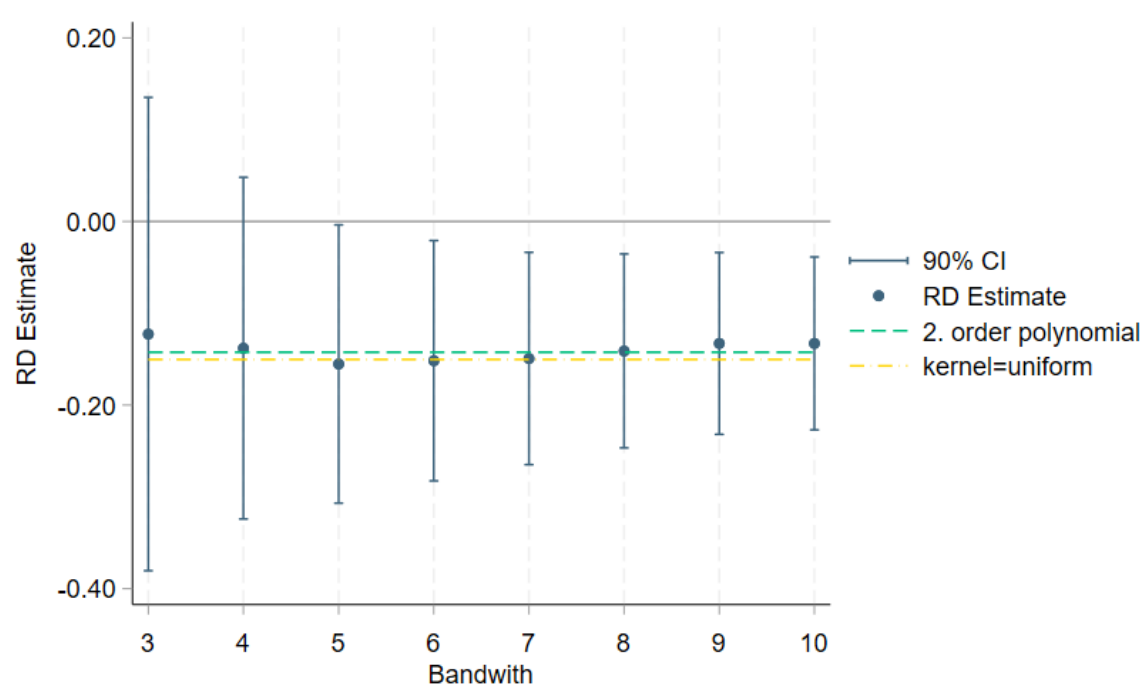

(c) Birth weight≥4.5 kg

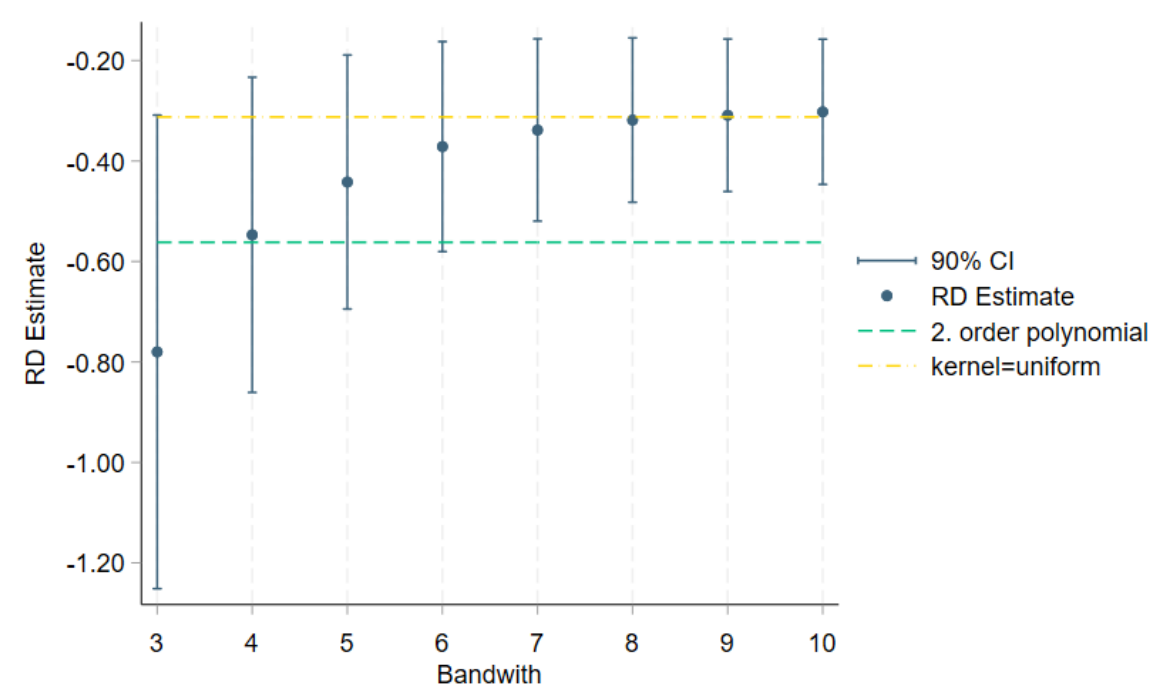

(d) Hemorrhage

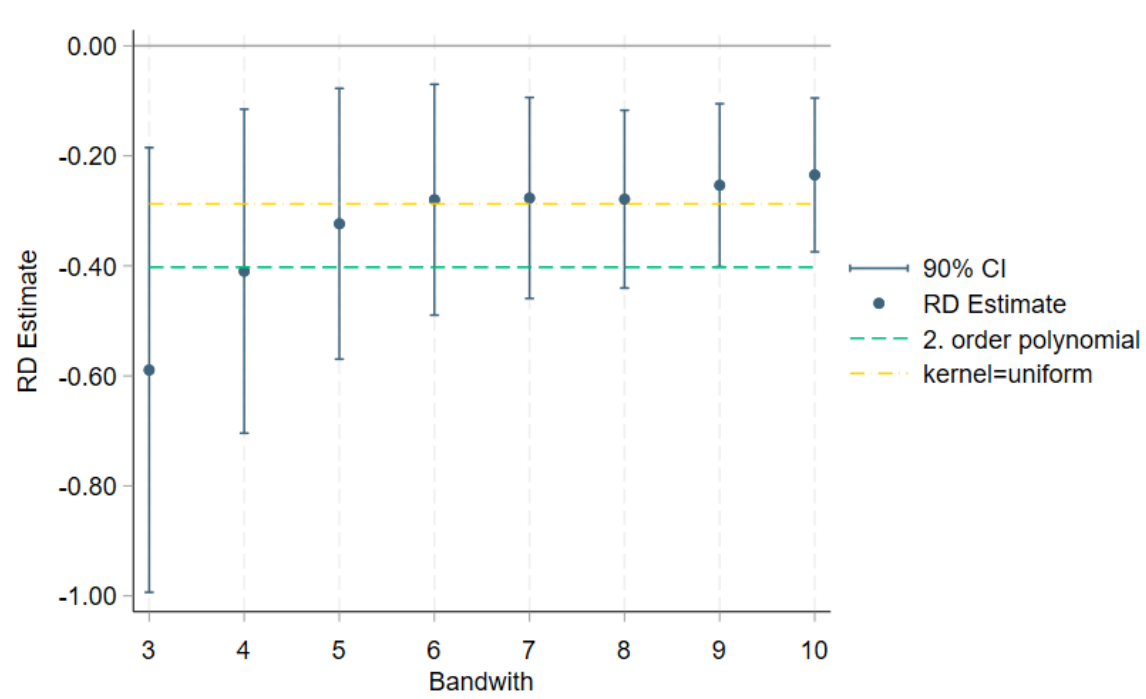

(e) Lacerations

Fig. D.10 Coefficient plots for immediate health outcomes

Notes: The figures are coefficient plots showing estimates from a fuzzy regression discontinuity design (RDD) as based on Equation 2 on various outcomes using varying specifications. Point estimates with higher polynomial order and different kernel are computed with a bandwidth of seven, as in our main specification. Standard errors are robust and clustered at the hospital-year level. Sample is the main one (all births in Denmark between 2011 and 2017 that occurred at 7 days after the estimated due date (EDD) or later).

D.6 Coefficient plots for short-run outcomes

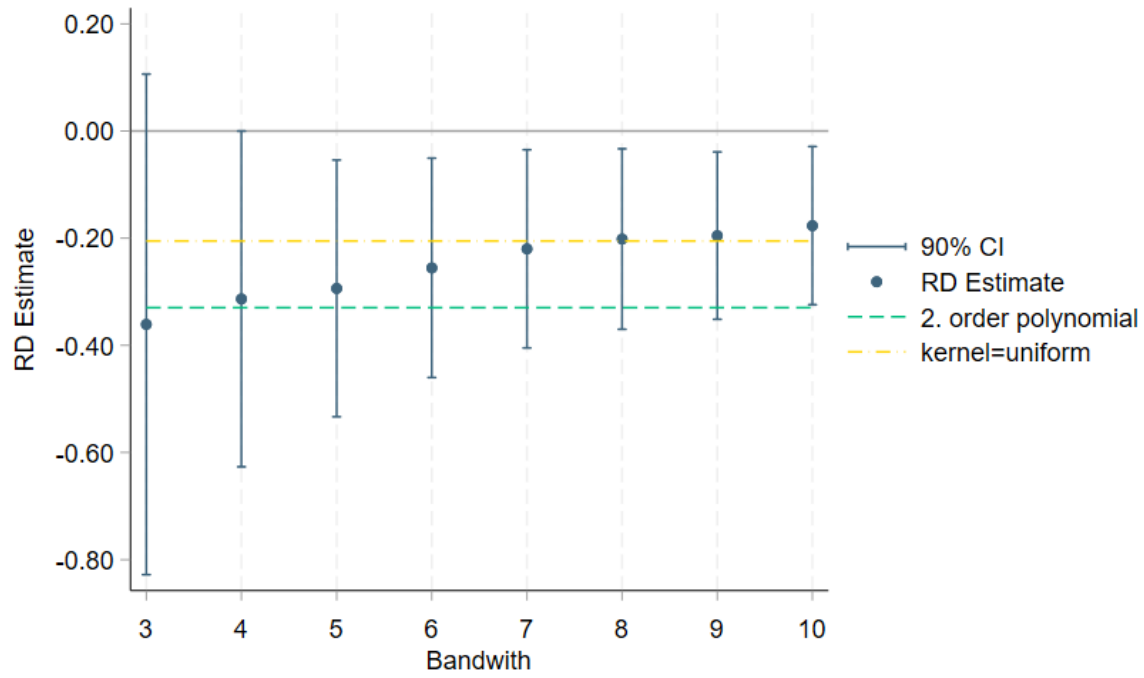

(a) EPDS>11

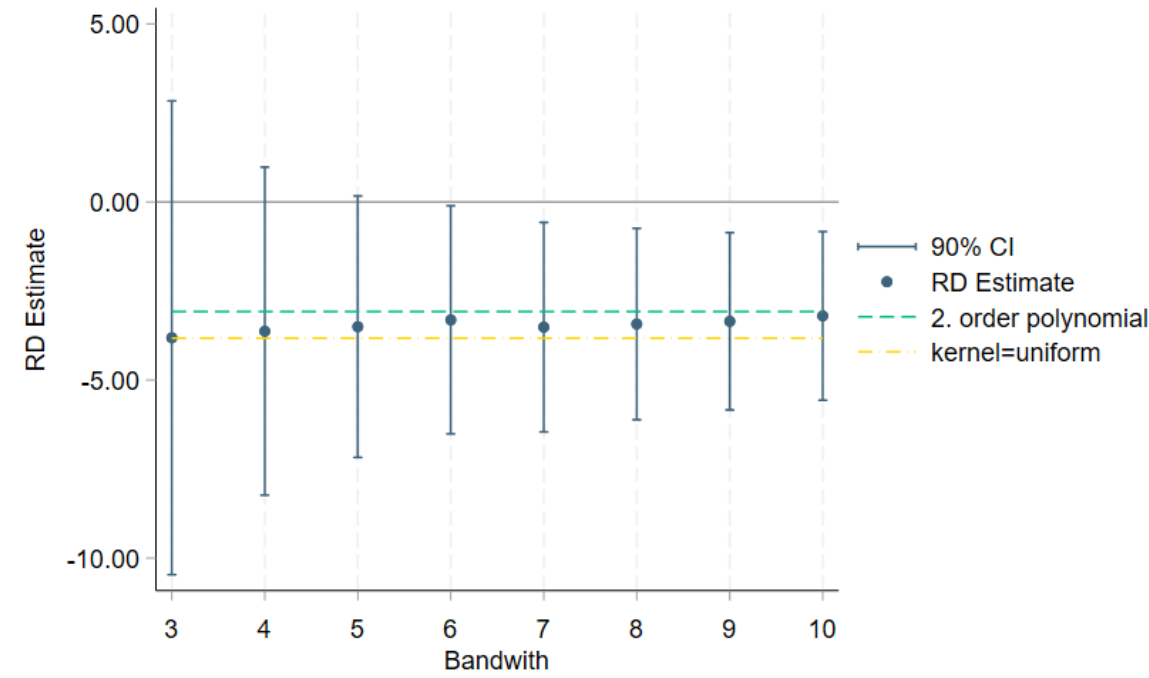

(b) EPDS score

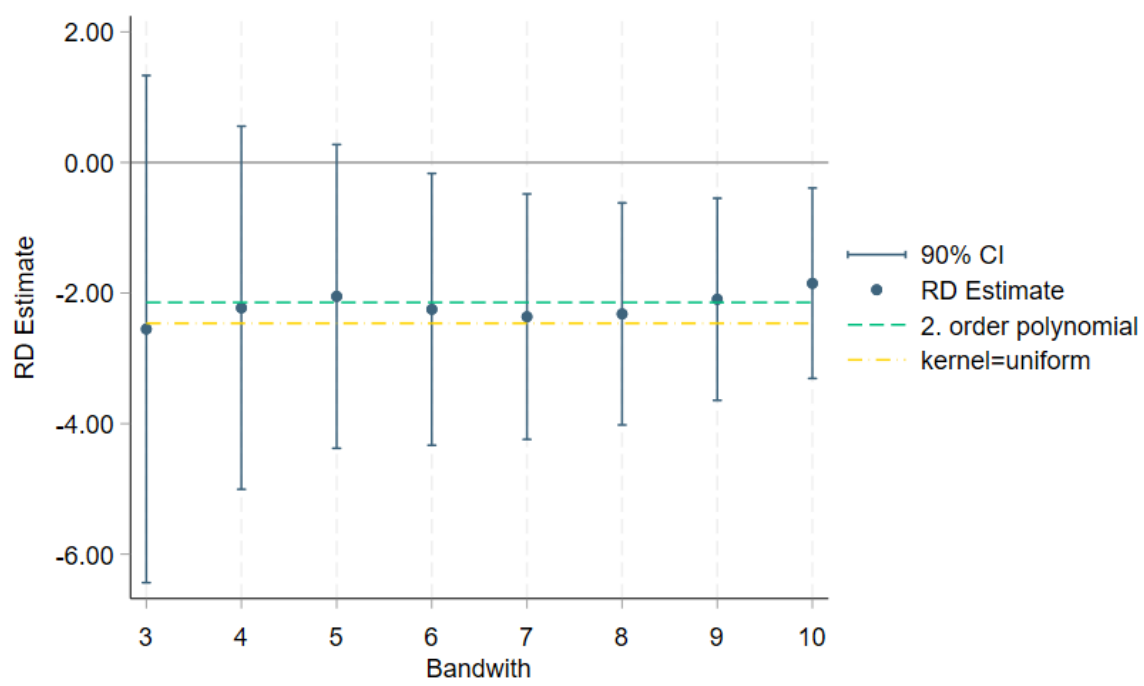

(c) Nurse visits (Total)

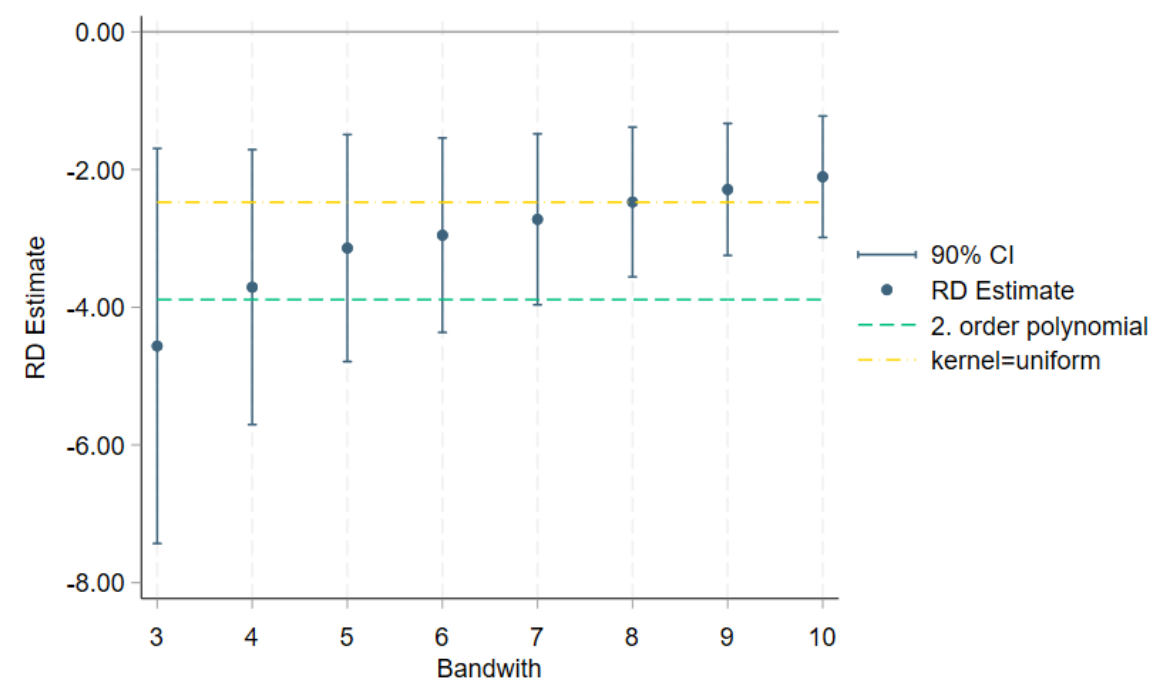

(d) Universal Nurse visits

Fig. D.11 Coefficient plots for various neonatal and first-year health outcomes

Notes: The figures are coefficient plots showing estimates from a fuzzy regression discontinuity design (RDD) as based on Equation 2 on various outcomes using varying specifications. Point estimates with higher polynomial order and different kernel are computed with a bandwidth of seven, as in our main specification. Standard errors are robust and clustered at the hospital-year level. Sample is the main one (all births in Denmark between 2011 and 2017 that occurred at 7 days after the estimated due date (EDD) or later) with information on the specific outcome considered.

D.7 Additional specifications

Tab. D.6 Immediate outcomes

	Fuzzy	Fuzzy	Fuzzy	Fuzzy	Fuzzy
Epidural	0.384**	0.424***	0.291*	0.335**	0.335***
	(0.168)	(0.143)	(0.163)	(0.138)	(0.117)
Emergency C-section	0.025	0.009	0.011	-0.005	-0.005
	(0.059)	(0.056)	(0.059)	(0.057)	(0.062)
Vacuum extraction	0.033	0.028	0.002	0.002	0.002
	(0.074)	(0.069)	(0.075)	(0.070)	(0.070)
Birth Weight (grams)	-326.950***	-300.113***	-300.301**	-272.538**	-272.538**
	(117.862)	(109.826)	(120.479)	(111.823)	(127.069)
Birthweight >4.5 kg	-0.158**	-0.152**	-0.153**	-0.149**	-0.149**
	(0.071)	(0.069)	(0.072)	(0.070)	(0.073)
Shoulder dystocia	-0.037**	-0.036**	-0.036**	-0.035**	-0.035**
	(0.017)	(0.016)	(0.017)	(0.017)	(0.015)
Lacerations	-0.277**	-0.292***	-0.259**	-0.277**	-0.277**
	(0.129)	(0.109)	(0.132)	(0.111)	(0.109)
Lacerations (3rd degree and higher)	0.009	0.006	0.002	-0.003	-0.003
	(0.043)	(0.042)	(0.044)	(0.043)	(0.044)
Hemorrhage	-0.303**	-0.366***	-0.273*	-0.338***	-0.338***
	(0.153)	(0.113)	(0.155)	(0.110)	(0.120)
Complications index	-0.017	-0.018	-0.015	-0.017	-0.017
	(0.020)	(0.019)	(0.019)	(0.019)	(0.020)
Uterus Rupture	-0.009	-0.009	-0.007	-0.007	-0.007
	(0.017)	(0.017)	(0.017)	(0.017)	(0.018)
Share sent in NICU	0.032	0.039	0.015	0.022	0.022
	(0.068)	(0.065)	(0.068)	(0.065)	(0.065)
Apgar <7	0.015	0.015	0.014	0.014	0.014
	(0.023)	(0.023)	(0.024)	(0.023)	(0.024)
Hospital Nights	0.756	0.857	0.324	0.437	0.437
	(0.554)	(0.542)	(0.540)	(0.515)	(0.528)
Controls	None	Fixed effects only	Medical controls only	Full controls	Full controls
Robust SEs (no clustering)	No	No	No	No	Yes

Notes: Standard errors are robust and clustered at the hospital-year level in all columns but the last one. * $p < 0.10$, ** $p < 0.05$, *** $p < 0.01$. This table displays RDD estimates which are obtained using our favorite specification with polynomial order of one and an epanechnikov kernel. The bandwidth is manually set to 7 for reasons explained in subsection D.3. The full set of controls is described in subsection C.1. The fixed effects included in the second columns are hospital fixed effects, year of birth fixed effects, month-of-birth fixed effects, and day of the week of birth fixed-effects. The medical controls are having a multiple birth, previously having given birth to a large child, previously having had a C-section, first-time mother, insufficient fetal growth, and pre-eclampsia.

Tab. D.7 Short-run outcomes

	Fuzzy	Fuzzy	Fuzzy	Fuzzy	Fuzzy
EPDS>11	-0.220*	-0.217*	-0.225*	-0.220*	-0.220**
	(0.116)	(0.113)	(0.116)	(0.112)	(0.106)
EPDS score	-3.313*	-3.226*	-3.630*	-3.514**	-3.514**
	(1.953)	(1.785)	(1.966)	(1.787)	(1.704)
Nurses visit (total)	-1.796	-1.926*	-2.248*	-2.362**	-2.362**
	(1.178)	(1.121)	(1.192)	(1.143)	(1.107)
Universal Nurses visit	-2.136**	-2.305***	-2.390***	-2.723***	-2.723***
	(0.891)	(0.750)	(0.899)	(0.755)	(0.676)
Nurses, additional visits	0.013	0.056	-0.192	0.057	0.057
	(0.982)	(0.965)	(0.991)	(0.996)	(0.944)
GP Visits (child)	-1.432	-1.097	-1.867	-1.518	-1.518
	(2.419)	(2.402)	(2.451)	(2.421)	(2.329)
Child: Any hosp. admission in first year	0.016	0.037	0.003	0.024	0.024
	(0.136)	(0.119)	(0.136)	(0.120)	(0.116)
Mom: Any hosp. admission in first year	0.007	0.003	-0.037	-0.045	-0.041
	(0.156)	(0.139)	(0.158)	(0.139)	(0.124)
Share Employed (Mother, 1 year post birth)	0.136	0.112	0.144	0.190	0.190
	(0.164)	(0.159)	(0.170)	(0.141)	(0.139)
Share Employed (Mother, 2 years post birth)	0.052	0.040	0.056	0.113	0.113
	(0.146)	(0.136)	(0.151)	(0.126)	(0.141)
Prob. birth two years after	-0.069	-0.059	-0.088	-0.077	-0.077
	(0.073)	(0.070)	(0.075)	(0.072)	(0.068)
Controls	None	Fixed effects only	Medical controls only	Full controls	Full controls
Robust SEs (without clustering)	No	No	No	No	Yes

Notes: Standard errors are robust and clustered at the hospital-year level in all columns but the last one. * $p < 0.10$, ** $p < 0.05$, *** $p < 0.01$. This table displays RDD estimates which are obtained using our favorite specification with polynomial order of one and an epanechnikov kernel. The bandwidth is manually set to 7 for reasons explained in subsection D.3. The full set of controls is described in subsection C.1. The fixed effects included in the second columns are hospital fixed effects, year of birth fixed effects, month-of-birth fixed effects, and day of the week of birth fixed-effects. The medical controls are having a multiple birth, previously having given birth to a large child, previously having had a C-section, first-time mother, insufficient fetal growth, and pre-eclampsia.

D.8 Placebo test: selection into the sample

Tab. D.8 Placebo checks

	Sharp	Obs
Elective CS	0.003	51427
	(0.009)	
Preeclampsia	0.003	51427
	(0.009)	
Insufficient Growth	0.014*	51427
	(0.007)	
Ever Induced	0.018	51427
	(0.015)	
Gestational diabetes	0.007	51427
	(0.011)	
Days after EDD	<7	
Sample	Main sample + gest. diabetes before EDD included	
Covariates	Yes	

Notes: This table shows effects for those not affected by the early induction guidelines (placebo). The effects displayed are sharp RDD estimates which are obtained using our favorite specification with polynomial order of one and an epanechnikov kernel. The bandwidth is manually set to 7 for reasons explained in Sub-section D.3. The set of controls is similar to that described Sub-section C.1, with the exception of preeclampsia, gestational diabetes, and insufficient growth recorded before the EDD that are excluded to circumvent bad controls issues. Standard errors are robust and clustered at the hospital-year level. * $p < 0.10$, ** $p < 0.05$, *** $p < 0.01$.

References

Alkmark, M., J. K. Keulen, J. C. Kortekaas, C. Bergh, J. Van Dillen, R. G. Duijnhoven, H. Hagberg, B. W. Mol, M. Molin, J. A. van der Post, et al. (2020). Induction of labour at 41 weeks or expectant management until 42 weeks: A systematic review and an individual participant data meta-analysis of randomised trials. *PLoS medicine 17*(12), e1003436.

Bassler, D., M. Briel, V. M. Montori, M. Lane, P. Glasziou, Q. Zhou, D. Heels-Ansdell, S. D. Walter, G. H. Guyatt, S.-. S. Group, et al. (2010). Stopping randomized trials early for benefit and estimation of treatment effects: Systematic review and meta-regression analysis. *Jama 303*(12), 1180–1187.

Burgos, J., L. Rodríguez, B. Otero, P. Cobos, C. Osuna, M. d. M. Centeno, J. C. Melchor, L. Fernández-Llebrez, and T. Martínez-Astorquiza (2012). Induction at 41 weeks increases the risk of caesarean section in a hospital with a low rate of caesarean sections. *The Journal of Maternal-Fetal & Neonatal Medicine 25*(9), 1716–1718.

Calonico, S., M. D. Cattaneo, M. H. Farrell, and R. Titiunik (2017). rdrobust: Software for regression-discontinuity designs. *The Stata Journal 17*(2), 372–404.

Coates, D., A. Makris, C. Catling, A. Henry, V. Scarf, N. Watts, D. Fox, P. Thirukumar, V. Wong, H. Russell, et al. (2020). A systematic scoping review of clinical indications for induction of labour. *PLoS One 15*(1), e0228196.

Dahlen, H. G., C. Thornton, S. Downe, A. De Jonge, A. Seijmonsbergen-Schermers, S. Tracy, M. Tracy, A. Bisits, and L. Peters (2021). Intrapartum interventions and outcomes for women and children following induction of labour at term in uncomplicated pregnancies: A 16-year population-based linked data study. *BMJ open 11*(6), e047040.

Daskalakis, G., D. Zacharakis, M. Simou, P. Pappa, S. Detorakis, S. Mesogitis, and A. Antsaklis (2014). Induction of labor versus expectant management for pregnancies beyond 41 weeks. *The Journal of Maternal-Fetal & Neonatal Medicine 27*(2), 173–176.

DSOG (2003). Den overvægtige gravide. Available: https://web.archive.org/web/20050121020122fw_/http://www.dsog.dk/files/overvaegtige%20gravide.htm (accessed 2 June 2024).

DSOG (2007a). Behandling af gestationel diabetes mellitus. Available: https://web.archive.org/web/20210216164230/http://gynobsguideline.dk/sandbjerg/Behandling%20af%20gestationel%20diabetes%20mellitus.pdf (accessed 23 April 2025).

DSOG (2007b). Foetus Magnus Suspicio (DO366). Available: https://web.archive.org/web/20220120230459/http://gynobsguideline.dk/sandbjerg/100411%20foetusmagnussuspicio%20Sandbjerg%202007.pdf (accessed 23 April 2025).

DSOG (2010). Kliniske retningslinier for diabetesbehandling ved graviditet hos kvinder med kendt diabetes diabetes (Type 1 og Type 2) før graviditeten. Available: https://web.archive.org/web/20190418072636/http://endocrinology.dk/kliniske%20retningslinier%20-%20diabetes%20og%20graviditet.pdf (accessed 23 April 2025).

DSOG (2017). Den overvægtige gravide . Available: https://www.dsog.dk/obstetrik (accessed 19 April 2024).

DSOG (2018). Foetus Magnus Suspicio (DO366). Available: https://www.dsog.dk/obstetrik (accessed 23 April 2025).

Gelisen, O., E. Caliskan, S. Dilbaz, E. Ozdas, B. Dilbaz, and A. Haberal (2005). Induction of labor with three different techniques at 41 weeks of gestation or spontaneous follow-up until 42 weeks in women with definitely unfavorable cervical scores. *European Journal of Obstetrics & Gynecology and Reproductive Biology 120*(2), 164–169.

Gelman, A. and G. Imbens (2019). Why high-order polynomials should not be used in regression discontinuity designs. *Journal of Business & Economic Statistics 37*(3), 447–456.

Goldberger, A. S. (2008). Selection bias in evaluating treatment effects: Some formal illustrations. In *Modelling and Evaluating Treatment Effects in Econometrics*. Emerald Group Publishing Limited.

Gregersen, M. K. (2024). Earlier routine induction of labor—consequences on mother and child morbidity. *Health Economics 33*(10), 2399–2418.

Hedegaard, M., Ø. Lidegaard, C. Skovlund, L. Mørch, and M. Hedegaard (2015). Perinatal outcomes following an earlier post-term labour induction policy: A historical cohort study. *BJOG: An International Journal of Obstetrics & Gynaecology 122*(10), 1377–1385.

Hedegaard, M., Ø. Lidegaard, C. W. Skovlund, L. S. Mørch, and M. Hedegaard (2014). Reduction in stillbirths at term after new birth induction paradigm: Results of a national intervention. *BMJ open 4*(8), e005785.

Heimstad, R., P. R. Romundstad, J. Hyett, L.-Å. Mattsson, and K. Å. Salvesen (2007). Women's experiences and attitudes towards expectant management and induction of labor for post-term pregnancy. *Acta obstetricia ET gynecologica scandinavica 86*(8), 950–956.

Heimstad, R., E. Skogvoll, L.-Å. Mattsson, O. J. Johansen, S. H. Eik-Nes, and K. Å. Salvesen (2007). Induction of labor or serial antenatal fetal monitoring in postterm pregnancy: a randomized controlled trial. *Obstetrics & Gynecology 109*(3), 609–617.

Hildingsson, I., A. Karlström, and A. Nystedt (2011). Women's experiences of induction of labour–findings from a swedish regional study. *Australian and New Zealand Journal of Obstetrics and Gynaecology 51*(2), 151–157.

Jungensen, D. R. (2011). Visitation ved højt BMI. *Jordmoderforeningen* (05). https://jordemoderforeningen.dk/tidsskriftsartikel/visitation-ved-hojt-bmi/.

Kenyon, S., L. Middleton, M. Skrybant, and T. Johnston (2019). When to induce late term pregnancies. *BMJ 367*.

Keulen, J., P. Nieuwkerk, J. Kortekaas, J. van Dillen, B. Mol, J. van der Post, and E. de Miranda (2021). What women want and why. women's preferences for induction of labour or expectant management in late-term pregnancy. *Women and Birth 34*(3), 250–256.

Keulen, J. K., A. Bruinsma, J. C. Kortekaas, J. Van Dillen, P. M. Bossuyt, M. A. Oudijk, R. G. Duijnhoven, A. H. Van Kaam, F. P. Vandenbussche, J. A. Van Der Post, et al. (2019). Induction of labour at 41 weeks versus expectant management until 42 weeks (index): multicentre, randomised non-inferiority trial. *BMJ 364*.

Lou, S., K. Carstensen, L. Hvidman, T. F. Jensen, L. Neumann, J.-G. Habben, and N. Uldbjerg (2021). "i guess baby was just too comfy in there. . . ": A qualitative study of women's experiences of elective late-term induction of labour. *Women and Birth 34*(3), 242–249.

Lou, S., L. Hvidman, N. Uldbjerg, L. Neumann, T. Jensen, J. Haben, and K. Carstensen (2019, 09). Women's experiences of postterm induction of labor: A systematic review of qualitative studies. *Birth 46*.

Muglu, J., H. Rather, D. Arroyo-Manzano, S. Bhattacharya, I. Balchin, A. Khalil, B. Thilaganathan, K. S. Khan, J. Zamora, and S. Thangaratinam (2019). Risks of stillbirth and neonatal death with advancing gestation at term: A systematic review and meta-analysis of cohort studies of 15 million pregnancies. *PLoS medicine 16*(7), e1002838.

Peters, L. L., C. Thornton, A. De Jonge, A. Khashan, M. Tracy, S. Downe, E. I. Feijen-de Jong, and H. G. Dahlen (2018). The effect of medical and operative birth interventions on child health outcomes in the first 28 days and up to 5 years of age: A linked data population-based cohort study. *Birth 45*(4), 347–357.

Place, K., L. Rahkonen, A. Tekay, K. Väyrynen, M.-R. Orden, M. Vääräsmäki, J. Uotila, K. Tihtonen, K. Rinne, K. Mäkikallio, et al. (2024). Labor induction at 41+ 0 gestational weeks or expectant management for the nulliparous woman: The finnish randomized controlled multicenter trial. *Acta Obstetricia et Gynecologica Scandinavica 103*(3), 505–511.

Rydahl, E., E. Declercq, M. Juhl, and R. D. Maimburg (2019). Routine induction in late-term pregnancies: follow-up of a danish induction of labour paradigm. *BMJ open 9*(12), e032815.

Rydahl, E., L. Eriksen, and M. Juhl (2019). Effects of induction of labor prior to post-term in low-risk pregnancies: A systematic review. *JBI Database of Systematic Reviews and Implementation Reports 17*(2), 170.

Schwarz, C., M. M. Gross, P. Heusser, and B. Berger (2016). Women's perceptions of induction of labour outcomes: Results of an online-survey in germany. *Midwifery 35*, 3–10.

Shetty, A., R. Burt, P. Rice, and A. Templeton (2005). Women's perceptions, expectations and satisfaction with induced labour—a questionnaire-based study. *European Journal of Obstetrics & Gynecology and Reproductive Biology 123*(1), 56–61.

SSI (2009). *Anbefalinger for svangreomsorgen.* The Danish Health Authorities (Sundhedsstyrelsen) Copenhagen, Denmark.

SSI (2013). *Anbefalinger for svangreomsorgen.* The Danish Health Authorities (Sundhedsstyrelsen) Copenhagen, Denmark.

Stommes, D., P. Aronow, and F. Sävje (2023). On the reliability of published findings using the regression discontinuity design in political science. *Research & Politics 10*(2), 20531680231166457.

Vinter, C. A., M. H. Tanvig, P. Damm, K. V. Naver, K. R. Andreasen, L. L. T. Andersen, S. Liest, S. Lunde, D. M. Jensen, and K. M. Renault (2012). Overvægtige gravide og komplikationer i relation til graviditet og fødsel. *Ugeskrift for Læger 174*(16), 1079–82.

Waldenström, U., I. Hildingsson, C. Rubertsson, and I. Rådestad (2004). A negative birth experience: prevalence and risk factors in a national sample. *Birth 31*(1), 17–27.

Wennerholm, U.-B., S. Saltvedt, A. Wessberg, M. Alkmark, C. Bergh, S. B. Wendel, H. Fadl, M. Jonsson, L. Ladfors, V. Sengpiel, et al. (2019). Induction of labour at 41 weeks versus expectant management and induction of labour at 42 weeks (SWEdish Post-term Induction Study, SWEPIS): multicentre, open label, randomised, superiority trial. *BMJ 367*.

Wolff, S. L., I. Lorentzen, A. P. Kaltoft, H. Schmidt, M. M. Jeppesen, and R. D. Maimburg (2016). Has perinatal outcome improved after introduction of a guideline in favour of routine induction and increased surveillance prior to 42 weeks of gestation?: A cross-sectional population-based registry study. *Sexual & Reproductive Healthcare 10*, 19–24.

Zizzo, A. R., I. Kirkegaard, A. Pinborg, and N. Ulbjerg (2017). Decline in stillbirths and perinatal mortality after implementation of a more aggressive induction policy in post-date pregnancies: A nationwide register study. *Acta Obstetricia et Gynecologica Scandinavica 96*(7), 862–867.
